# Automated three-component synthesis of a library of γ-lactams

**DOI:** 10.3762/bjoc.8.206

**Published:** 2012-10-19

**Authors:** Erik Fenster, David Hill, Oliver Reiser, Jeffrey Aubé

**Affiliations:** 1 Center of Excellence in Chemical Methodologies and Library Development, the University of Kansas, 2034 Becker Drive, Lawrence Kansas, 66047, USA; 2 Institut für Organische Chemie, Universität Regensburg, Universitätsstrasse 31, 93053, Regensburg, Germany

**Keywords:** γ-lactam, maleimide, organocatalysis, parallel synthesis, reductive amination

## Abstract

A three-component method for the synthesis of γ-lactams from commercially available maleimides, aldehydes, and amines was adapted to parallel library synthesis. Improvements to the chemistry over previous efforts include the optimization of the method to a one-pot process, the management of by-products and excess reagents, the development of an automated parallel sequence, and the adaption of the method to permit the preparation of enantiomerically enriched products. These efforts culminated in the preparation of a library of 169 γ-lactams.

## Introduction

In recent years, the rapid access to structurally diverse and complex small molecules has grown in importance within the context of high-throughput screening of biologically relevant targets. The need for such compounds, both as pharmacological probes and as starting points for drug-discovery campaigns, has primarily fuelled this interest, while enabling technologies, such as diversity-oriented synthesis (DOS), have improved access to small-molecule libraries [1–5].

**Scheme 1:**
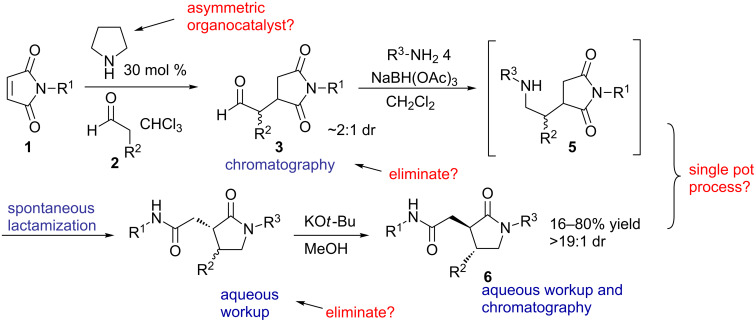
Three-step sequence for the preparation of γ-lactams from maleimides, aldehydes and amines. Potential improvements are indicated in red.

Compounds containing a γ-lactam moiety have been significant in the treatment of epilepsy [6–7], HIV [8–9], neurodegenerative disease and depression [10–11]. Having identified the lactam ring as a target of opportunity in chemical-screening efforts, we previously reported a method to prepare γ-lactams from readily available maleimides, aldehydes and amines [12]. The method involved a three-step stepwise sequence involving an organocatalyzed Michael addition, a reductive amination/intramolecular lactamization, and an epimerization step (Scheme 1). It culminated in the preparation of a 43-member library. Although this method permitted relatively easy access to highly substituted racemic γ-lactams, it required manual manipulations of the intermediate products in each step in the form of workups and purifications. Accordingly, we wished to develop a streamlined approach that would provide access to larger numbers and quantities of diverse γ-lactams, preferentially in enantiomerically pure form. Ideally, this would take the form of a single-pot process utilizing automation. We now report improvements to this process, which (1) generates enantiomerically enriched compounds, (2) eliminates the need for intermediate purifications, (3) simplifies the method to a three-step one-pot sequence, and (4) allows for the magnification of the library scale through the use of automation.

## Results and Discussion

We began by probing the potential for the above-mentioned improvements to our previously reported method by (a) exploring the possibility for an asymmetric organocatalyzed reaction in the Michael addition step, (b) combining the individual three steps, and (c) automating the process to produce a demonstrative 256 member γ-lactam library.

### Asymmetric organocatalyzed Michael addition

**Scheme 2:**
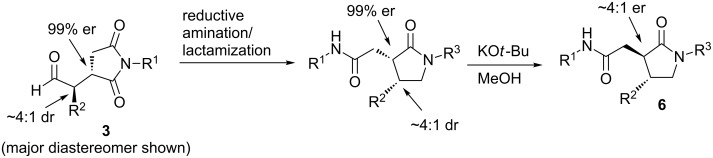
The transfer of the diastereoselective ratio of **3** to the enantioselectivity of the overall process in the synthesis of γ-lactams **6**.

The success of pyrrolidine as the organocatalyst for the Michael addition of enolizable aldehydes to maleimides in the original method suggested the possibility of producing chiral γ-lactams through the use of a chiral proline-like organocatalysts. In fact, Córdova and co-workers have previously established a protocol for such a process [13]. Based on this initial protocol, we explored the use of various chiral amines in the conjugate addition reaction of propionaldehyde (**2{*1*}**) to *N*-phenylmaleimide (**1{*1*}**) (Table 1). Although the achiral pyrrolidine was found to be an efficient catalyst in the reaction [12], this led to a ~1:1 mixture of racemic succinimide diastereomers **3{*1,1*}**, even at lowered temperatures (Table 1, entries 1 and 2). While a number of chiral amines failed to produce any significant amounts of the desired succinimide product (Table 1, entries 3–9), we were pleased to find that the use of protected diphenylprolinol catalyst **I** at room temperature produced **3{*1,1*}** in high yields and with high enantioselectivity, albeit with low diastereoselectivity. Such a low diastereoselectivity was of no concern in the originally reported procedure since the final potassium *tert*-butoxide promoted epimerization step produced a racemic mixture of a single diastereomer. In the case of an asymmetric synthesis, however, the initial diasteromeric ratio would be reflected in the enantiomeric ratio of the final products when the downstream epimerization step is taken into account; this is illustrated for the limiting case of high facial selectivity with respect to the maleimide component in Scheme 2. Consequently, higher diastereoselectivity was required for the synthesis of enantioenriched γ-lactams. Indeed, **3{*1,1*}** could be produced with moderate diastereoselectivity when the reaction was performed at 0 °C over a prolonged length of time (Table 1, entry 11) [14]. The use other solvents (THF, acetonitrile) failed to either produce any product (Table 1, entry 12) or lead to higher diastereoselectivity (Table 1, entry 13). We considered that lowering reaction temperatures in chloroform may lead to increased diastereoselectivities, but concluded that the lengthy reaction times necessitated under these conditions would have made the overall process inefficient. Although future work will explore additional catalysts and reaction conditions to improve this diastereoselectivity, we elected at this point to explore the additional steps to produce γ-lactams with the modest enantiomeric enrichment in hand.

**Table 1:** Asymmetric organocatalytic addition of propionaldehyde (**2{*1*}**) to *N*-phenylmaleimide (**1{*1*}**).

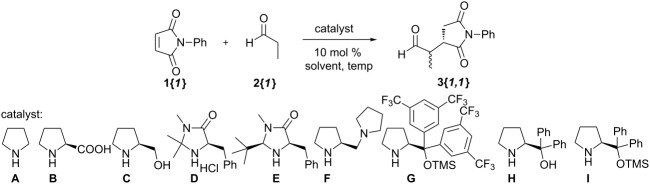							

Entry	Catalyst	Solvent	Temp (°C)	Time (h)	Yield (%)	dr	ee (%)^a^

1	**A**^b^	CHCl_3_	61	2	74	1:1.1	—
2	**A**	CHCl_3_	rt	16	72	1:1.2	—
3	**B**	CHCl_3_	61	12	trace	—	—
4	**C**	CHCl_3_	61	12	NR	—	—
5	**D**	CHCl_3_	61	12	NR	—	—
6	**E**	CHCl_3_	61	12	NR	—	—
7	**F**	CHCl_3_	rt	24	30	1:2.0	60
8	**G**	CHCl_3_	61	12	NR	—	—
9	**H**	CHCl_3_	61	12	trace	—	—
10	**I**	CHCl_3_	rt	12	88	1:2.0	96
11	**I**	CHCl_3_	0	36	90	1:4.2	99
12	**I**	THF	61	12	NR	—	—
13	**I**	CH_3_CN	0	36	72	1:2.5	90

^a^Determined by chiral phase HPLC analysis. ^b^30 mol % of the catalyst was used.

### Combination of three discrete steps into a single-pot process

**Scheme 3:**
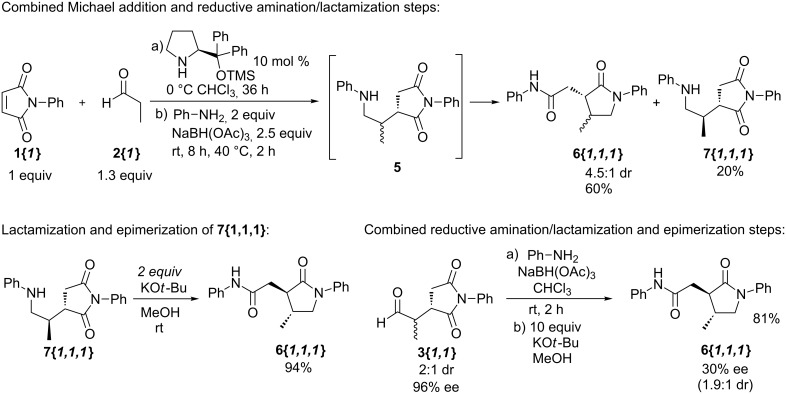
Combination of the Michael addition step with the reductive amination/lactamization step and of the reductive amination/lactamization step with the epimerization step.

We next turned our attention to combining the initial Michael addition step with the remaining reductive amination/lactamization and epimerization steps. In the original procedure, the reductive amination/lactamization reaction was performed in methylene chloride [12]. However, in order to combine this step with the previous Michael addition step, we thought to perform the reaction in chloroform (Scheme 3). In fact, when the Michael addition reaction between propionaldehyde (**2{*1*}**) and *N*-phenylmaleimide (**1{*1*}**) was performed in chloroform at 0 °C (using the protected diphenylprolinol catalyst **I**), followed by addition of aniline (**4{*1*}**) and sodium triacetoxyborohydride after 36 h, the desired γ-lactam **6{*1,1,1*}** was obtained in 60% yield (as a 4.5:1 mixture of diastereomers). However, 20% of the succinimide product **7{*1,1,1*}** was also isolated as a single diastereomer, resulting from incomplete transamidation.

Although the reductive amination occurred readily at room temperature, the lactamization appeared to occur more slowly than we had anticipated. In investigating individually the reductive amination/lactamization step using **3{*1,1*}** with aniline (**4{*1*}**) (Table 2), we found that the reaction required heating at 40 °C for at least 8 h for complete lactamization to be achieved (Table 2, entry 3). Furthermore, the isolation of succinimide **7{*1,1,1*}** as a single diastereomer at reduced temperatures (Table 2, entry 1) or reaction times (Table 2, entry 2) seemed to indicate that the lactamization in forming the *syn*-diastereomer of **6{*1,1,1*}** occurs at a slower rate than that for the *anti*-diastereomer.

**Table 2:** Reductive amination/lactamization of **3{*1*}** with aniline (**4{*1*}**).

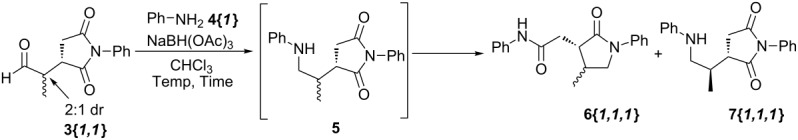					

Entry	Temp (°C)	Time (h)	**6{*1,1,1*}**		**7{*1,1,1*}**
			Yield (%)	dr	Yield (%)

1	rt	8	45	1.1:1	40
2	40	2	60	1.6:1	22
3	40	8	76	2.0:1	—

However, we found that subjecting succinimide **7{*1,1,1*}** by itself to potassium *tert*-butoxide in methanol promoted the lactamization at room temperature in addition to the final epimerization to produce **6{*1,1,1*}** as a single diastereomer (Scheme 3). Consequently, it became clear that complete lactamization in the previous step would not necessarily be required if the reductive amination/lactamization and epimerization steps were combined. Thus, subjecting **3{*1,1,1*}** to the reductive amination conditions for 2 h at room temperature, followed by addition of potassium *tert*-butoxide (after a solvent change from CHCl_3_ to MeOH) provided the γ-lactam **6{*1,1,1*}** in 81% (Scheme 3) [15].

**Scheme 4:**
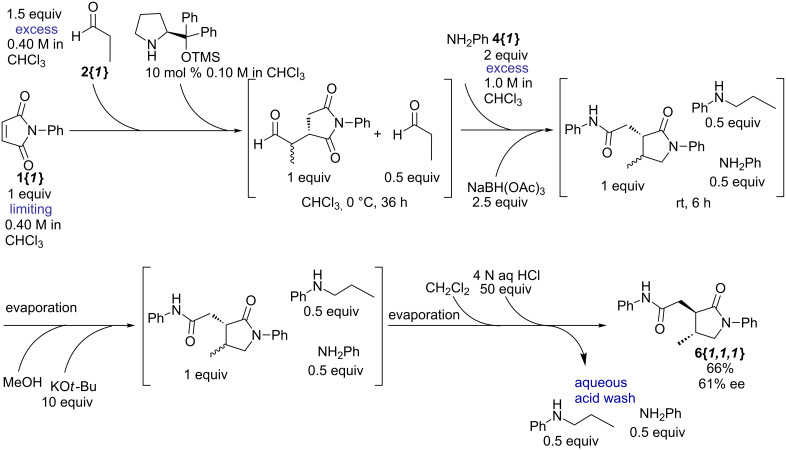
Combination of the Michael addition, the reductive amination/lactamization, and the epimerization step in a single-pot process.

Having demonstrated the ability to combine the first two steps as well as the last two steps, our attention was turned to combine all three steps in a single-pot process (Scheme 4). At this stage, we also investigated the possibility for removal of excess reagents and byproducts in order to render the overall process more efficient for parallel synthesis. If *N*-phenylmaleimide (**1{*1*}**) was to be used as a limiting reagent and propionaldehyde (**2{*1*}**) and aniline (**4{*1*}**) were used (with **4{*1*}** in excess relative to **2{*1*}**), then at the end of the one-pot process we should be left with the desired γ-lactam **6{*1,1,1*}** and with only amines (aniline and *N*-propylaniline) as excess byproducts, which could be removed with a simple aqueous acid wash along with the remaining catalyst, borate salts, and potassium *tert*-butoxide. Thus, *N*-phenylmaleimide (**1{*1*}**) (1 equiv) was stirred with an excess of propionaldehyde (**2{*1*}**) (1.5 equiv) in the presence of the silylated diphenylprolinol catalyst (10 mol %) for 36 h at 0 °C, after which an excess of aniline (2 equiv) and sodium triacetoxyborohydride (2.5 equiv) were added to the reaction mixture. After being stirred for 6 h at room temperature, the chloroform solvent was removed in vacuo, replaced with methanol, and potassium *tert*-butoxide (10 equiv) was added. After 12 h at room temperature, the methanol solvent was evaporated in vacuo, the resulting residue was redisolved in methylene chloride, and the solution was washed with 4 N HCl. Following simple filtration through a plug of silica gel, the γ-lactam **6{*1,1,1*}** was obtained in 66% yield (with 80% es; % es = % major enantiomer, corresponding to 60% ee). Overall, the above-mentioned one-pot sequence, in which both workups and purifications are minimized, proved serviceable in the desired parallel-synthesis sequence.

### Parallel library synthesis

The combined sequence was next attempted as a rehearsal 2 × 3 × 3 validation library by using a Chemspeed Accelerator SLT-100 synthesizer (Table 3). In general, substrates which varied in their steric and electronic nature were chosen (alkyl and aryl groups). By using the three-step single-pot sequence described above on the Chemspeed platform (0.400 mmol scale), the resulting 18 crude γ-lactams **6** were subjected to preparative HPLC purification. In general, yields in the range of 35–58% were obtained, with the exception of those reactions that used phenylacetaldehyde **2{*2*}** (yields in the range of 4–12%). Although in our previous work aryl acetaldehydes were found to produce decent yields with pyrrolidine [12], similar results were not obtained when the protected diphenylprolinol catalyst **I** was used. Except for the experiment in Table 3, entry 4, chemical purities >85% were observed for all of the isolated products. Although most of the reactions proceeded with high diastereoselectivities following the epimerization step (>19:1), this was not the case for all library products prepared during the production run described in the following section, as evident from small impurities (ca. ≤10%) observable in the ^13^C NMR spectra of the final products (Supporting Information File 1). Such products were deemed suitable for screening purposes. Finally, enantiomeric purity measurements were obtained for six selected γ-lactam products, which ranged between 62 and 84% es.

**Table 3:** Chemspeed rehearsal 2 × 3 × 3 library of γ-lactams **6**.

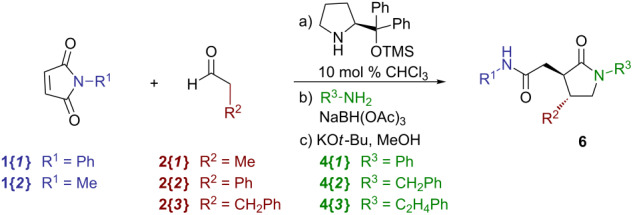							

Entry	Maleimide	Aldehyde	Amine	Product	Yield (%)^a^	Purity (%)^b^	es (%)^c,d^

1	**1{*1*}**	**2{*1*}**	**4{*1*}**	**6{*1,1,1*}**	45	100	62
2	**1{*1*}**	**2{*1*}**	**4{*2*}**	**6{*1,1,2*}**	50	100	n.d.
3	**1{*1*}**	**2{*1*}**	**4{*3*}**	**6{*1,1,3*}**	59	100	66
4	**1{*1*}**	**2{*2*}**	**4{*1*}**	**6{*1,2,1*}**	10	83.1	n.d.
5	**1{*1*}**	**2{*2*}**	**4{*2*}**	**6{*1,2,2*}**	10	100	n.d.
6	**1{*1*}**	**2{*2*}**	**4{*3*}**	**6{*1,2,3*}**	12	100	n.d.
7	**1{*1*}**	**2{*3*}**	**4{*1*}**	**6{*1,3,1*}**	58	100	75
8	**1{*1*}**	**2{*3*}**	**4{*2*}**	**6{*1,3,2*}**	52	96.6	84
9	**1{*1*}**	**2{*3*}**	**4{*3*}**	**6{*1,3,3*}**	58	93.7	n.d.
10	**1{*2*}**	**2{*1*}**	**4{*1*}**	**6{*2,1,1*}**	50	85.0	n.d.
11	**1{*2*}**	**2{*1*}**	**4{*2*}**	**6{*2,1,2*}**	52	85.4	n.d.
12	**1{*2*}**	**2{*1*}**	**4{*3*}**	**6{*2,1,3*}**	43	95.7	71
13	**1{*2*}**	**2{*2*}**	**4{*1*}**	**6{*2,2,1*}**	5	90.6	n.d.
14	**1{*2*}**	**2{*2*}**	**4{*2*}**	**6{*2,2,2*}**	4	100	n.d.
15	**1{*2*}**	**2{*2*}**	**4{*3*}**	**6{*2,2,3*}**	8	91.7	n.d.
16	**1{*2*}**	**2{*3*}**	**4{*1*}**	**6{*2,3,1*}**	35	100	n.d.
17	**1{*2*}**	**2{*3*}**	**4{*2*}**	**6{*2,3,2*}**	39	100	n.d.
18	**1{*2*}**	**2{*3*}**	**4{*3*}**	**6{*2,3,3*}**	42	100	74

^a^Purified by an automated preparative reverse phase HPLC (detected by mass spectroscopy). ^b^Purity was determined by HPLC with peak area (UV) at 214 nm. ^c^30 mol % of the catalyst was used. ^d^Determined by chiral phase HPLC analysis.

**Scheme 5:**
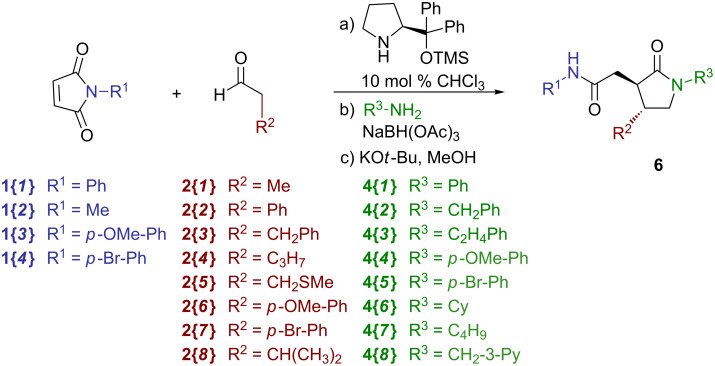
Chemspeed 4 × 8 × 8 library of γ-lactams **6**.

Overall, the results of the validation efforts demonstrated both the success of the one-pot automated method, as well as the scope of substrates suitable for a larger library set. With these considerations, we planned for a 4 × 8 × 8 = 256 member library of γ-lactams **6** on the Chemspeed platform with the conditions evaluated in the rehearsal library. A number of maleimides **1**, aldehydes **2** and amines **3** were chosen (Scheme 5) based on differences in their structure and polarity. Using the validated conditions, the Chemspeed was programmed to run four sets of 4 maleimides × 2 aldehydes × 8 amines (64 compounds each) for a total of 256 attempted syntheses. Upon completion of the four 64-compound runs, the reaction mixtures were purified by preparative HPLC, which afforded 169 compounds that met our goals of quantities >20 mg and purities >90%, in each case starting with 0.400 mmol of maleimides **1** [16]. Further analysis of the results showed that the arylacetaldehydes (**2{*2*}**, **2{*6*}** and **2{*7*}**) produced either lowered or zero amounts of material in the reactions for which these were utilized, with no discrepancy of the substitution on the aryl ring. Additionally, 3-pycolylamine **4{*8*}** yielded little or no material in the reactions for which it was used. With these exceptions, the process, in general, yielded products in the 15–70% range.

## Conclusion

In summary, our previously reported method for the three-component preparation of γ-lactams from commercially available maleimides, aldehydes and amines was improved and adapted to automated parallel library synthesis. Improvements to the chemistry over the previous method include the introduction of asymmetry in the form of an organocatalyzed Michael addition, the removal of the main by-products and excess reagents by using an aqueous acid wash, and the optimization for a one-pot process. These efforts allowed the efficient use of automation for parallel library synthesis, culminating in the preparation of a library of 169 γ-lactams.

## Experimental

### General

All single-vessel reactions were performed under an argon atmosphere in flame-dried glassware. The glass syringes and stainless-steel needles used for handling anhydrous solvents and reagents were oven dried, cooled in a desiccator, and flushed with dry nitrogen prior to use. Plastic syringes were flushed with dry nitrogen before use. Thin-layer chromatography (TLC) was performed on Analtech Uniplate Silica Gel GHLF 250 μm precoated TLC plates. All library syntheses using automated technology were performed by using a Chemspeed Accelerator SLT-100 Automated Synthesizer under an argon atmosphere in vacuum dried (50 °C, 9 mmHg) 13 mL reactor vessels. Parallel evaporations were performed by using a GeneVac EZ-2 plus evaporator. Automated preparative reverse-phase HPLC purification was performed by using a Waters 2767 Mass Directed Fractionation system (2767 sample manager, 2525 Binary Pump, 515 Make-up pump) with a Waters ZQ quadrapole spectrometer and detected by UV (270 nm, Waters Xterra MS C-18 column, 19 × 150 mm, elution with the appropriate gradient of acetonitrile in pH 9.8 buffered aqueous ammonium formate at 18 mL·min^−1^ flow rate). Purity was determined by reverse-phase HPLC with peak area (UV) at 214 nm by using a Waters Alliance 2795 system (Waters Xterra MS C-18 column, 4.6 × 150 mm, elution with a linear gradient of 5% acetonitrile in pH 9.8 buffered aqueous ammonium formate to 100% acetonitrile at 1.0 mL·min^−1^ flow rate). Purity was determined by reverse-phase HPLC with peak area (UV) at 214 nm by using a Waters Alliance 2795 system (Waters Xterra MS C-18 column, 4.6 × 150 mm, elution with a linear gradient of 5% acetonitrile in pH 9.8 buffered aqueous ammonium formate to 100% acetonitrile at 1.0 mL·min^−1^ flow rate). Methylene chloride, acetonitrile, methanol and tetrahydrofuran were purified by using an Innovative Technology Pure-Solv 400 solvent purification system. Chloroform was purified by distillation over calcium hydride. The maleimides **1**, the aldehydes **2**, the amines **3** and the chiral amine organocatalyst (**A**–**I**) were purchased from the Aldrich Chemical Co. and used without further purification. Melting points were performed by using an Optimelt (MPA100) automated melting-point system (Sanford Research Systems) and are uncorrected. Optical rotations of samples were performed by using a Rudolph Research Analytical Autopol IV automatic polarimeter at 589 nm. Infrared (IR) spectra were obtained using a Perkin-Elmer Spectrum 100 FT-IR spectrometer with a UATR application. Chiral HPLC measurements were performed on a Shimadzu SCL-10A system with a SPD-10AV UV–vis detector and hexane/isopropanol as the solvent mobile phase. Proton nuclear magnetic resonance (^1^H NMR) and carbon nuclear magnetic resonance (^13^C NMR) spectra were recorded in deuterochloroform by using a Bruker AV-400 or a Bruker DRX-500. Chemical shifts are reported in parts per million (ppm) and are referenced to the centerline of deuterochloroform (δ 7.24 ppm ^1^H NMR, 77.0 ppm ^13^C NMR). Coupling constants are given in hertz (Hz). Low-resolution mass spectra (MS) and high-resolution mass spectra (HRMS) were recorded on a Waters LCT Premier TOF spectrometer for electrospray ionization (ESI).

#### General procedure for the asymmetric organocatalyzed Michael addition of aldehyde **2{*1*}** to maleimide **1{*1*}** to produce **3{*1*,*1*}** (Table 1)

Propionaldehyde (**2{*1*}**) (31.2 μL, 0.433 mmol, 1.5 equiv) was added to a solution of *N*-phenylmaleimide (**1{*1*}**) (50.0 mg, 0.289 mmol, 1.0 equiv) in chloroform (3 mL) at 0 °C, followed by the appropriate chiral amine (**A**–**I**) (0.0289 mmol, 0.1 equiv) and the reaction mixture was stirred at the appropriate temperature for the appropriate length of time listed in Table 1. Water was added upon completion of the reaction and the resulting mixture was extracted with methylene chloride (3 × 5 mL). The methylene chloride extracts were combined, dried over sodium sulfate and concentrated in vacuo to yield a yellow liquid. The crude liquid was purified by column chromatography (2/3 ethyl acetate–hexane) to yield the succinimide **3{*1,1*}** as a clear colorless liquid composed of a mixture of two diastereomers, which were separated by further chromatographic purification.

**(*R*)-2-((*S*)-2,5-Dioxo-1-phenylpyrrolidin-3-yl)propanal (3{*1*,*1*}) (major diastereomer):** 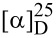 +74.0 (*c* 1.10, CHCl_3_); ^1^H NMR (400 MHz, CDCl_3_) δ 9.67 (s, 1H), 7.47 (m, 2H), 7.39 (m, 1H), 7.35–7.23 (m, 2H), 3.36 (m, 1H), 3.13 (m, 1H), 2.98 (dd, *J* = 18.5, 9.7 Hz, 1H), 2.51 (m, 1H), 1.30 (d, *J* = 7.6 Hz, 3H); ^13^C NMR (100 MHz, CDCl_3_) δ 201.4, 177.5, 175.0, 131.8, 129.1, 128.6, 126.4, 46.9, 39.4, 31.5, 9.7; IR (neat): 2971, 2940, 1774, 1701 cm^−1^; MS (ESI^+^) *m*/*e* 232.1 [M + H]^+^; HRMS (ESI^+^) calcd for C_13_H_14_NO_3_ [M + H]^+^: 232.0974; found: 232.0988.

**(*S*)-2-((*S*)-2,5-Dioxo-1-phenylpyrrolidin-3-yl)propanal (3{*1*,*1*}) (minor diastereomer):** 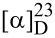 +43.3 (*c* 0.80, CHCl_3_); ^1^H NMR (400 MHz, CDCl_3_) δ 9.57 (s, 1H), 7.47 (m, 2H), 7.39 (m, 1H), 7.28 (m, 2H), 3.26–3.15 (m, *J* = 13.0, 6.5 Hz, 1H), 3.10–3.00 (m, 1H), 2.87 (dd, *J* = 18.1, 9.6 Hz, 1H), 2.54 (dd, *J* = 18.1, 5.6 Hz, 1H), 1.33 (d, *J* = 7.8 Hz, 3H); ^13^C NMR (100 MHz, CDCl_3_) δ 201.8, 177.6, 175.1, 132.0, 129.0, 128.5, 126.5, 46.8, 40.6, 31.5, 11.2; IR (neat): 2973, 2941, 1773, 1701 cm^−1^; MS (ESI^+^) *m*/*e* 232.1 [M + H]^+^; HRMS (ESI^+^) calcd for C_13_H_14_NO_3_ [M + H]^+^: 232.0974; found: 232.0985.

#### General procedure for the reductive amination/lactamization of **3{*1,1*}** with aniline (**4{*1*}**) (Table 2)

Aniline (**4{*1*}**) (38.4 μL, 0.422 mmol, 1.5 equiv) and sodium triacetoxyborohydride (119 mg, 0.562 mmol, 2.0 equiv) were added to a solution of succinimide **3{*1,1*}** (65.0 mg, 0.281 mmol, 1.0 equiv) in chloroform (3 mL) and the reaction mixture was stirred at the appropriate temperature for the appropriate length of time listed in Table 2. A 1 N aqueous solution of sodium hydroxide (3 mL) was added and the reaction mixture was extracted with methylene chloride (3 × 2 mL). The methylene chloride extracts were combined, dried over sodium sulfate and concentrated in vacuo to yield a yellow liquid. The crude liquid was purified by column chromatography (1/1 ethyl acetate-hexane) to yield **6{*1,1,1*}** as a clear colorless liquid composed of a mixture of two diastereomers and **7{*1,1,1*}** as a clear colorless liquid.

**2-((3*S*,4*R*)-4-Methyl-2-oxo-1-phenylpyrrolidin-3-yl)-*N*-phenylacetamide** (**6{*1,1,1*}**) **(major syn diastereomer): **^1^H NMR (400 MHz, CDCl_3_) δ 9.44 (s, 1H), 7.62–7.53 (m, 4H), 7.42–7.36 (m, *J* = 8.0 Hz, 2H), 7.32–7.24 (m, 2H), 7.22–7.16 (m, *J* = 9.2, 5.5 Hz, 1H), 7.10–7.03 (m, *J* = 7.4 Hz, 1H), 4.05 (dd, *J* = 9.6, 6.0 Hz, 1H), 3.43 (dd, *J* = 9.7, 0.9 Hz, 1H), 3.29–3.22 (m, *J* = 7.8, 5.4 Hz, 1H), 3.00–2.93 (m, 1H), 2.82–2.74 (m, 1H), 2.51 (dd, *J* = 15.3, 5.3 Hz, 1H), 1.09 (d, *J* = 3.1 Hz, 3H); ^13^C NMR (100 MHz, CDCl_3_) δ 175.4, 170.0, 139.2, 138.4, 129.0, 128.9, 125.2, 123.9, 120.3, 119.8, 54.9, 44.7, 35.1, 30.7, 14.9; IR (neat): 3465, 3263, 2932, 2855, 1660 cm^−1^; MS (ESI^+^) *m*/*e* 309.2 [M + H]^+^; HRMS (ESI^+^) calcd for C_19_H_21_N_2_O_2_ [M + H]^+^: 309.1603; found: 309.1606.

**2-((3*S*,4*S*)-4-Methyl-2-oxo-1-phenylpyrrolidin-3-yl)-*N*-phenylacetamide** (**6{*1,1,1*}**) **(minor anti diastereomer): **^1^H NMR (400 MHz, CDCl_3_) δ 9.58 (s, 1H), 7.62–7.51 (m, 4H), 7.41–7.33 (m, 2H), 7.30–7.22 (m, *J* = 10.3, 5.5 Hz, 2H), 7.22–7.12 (m, *J* = 7.4 Hz, 1H), 7.10–6.98 (m, *J* = 7.4 Hz, 1H), 3.79 (dd, *J* = 9.4, 8.0 Hz, 1H), 3.42 (t, *J* = 9.5 Hz, 1H), 2.85 (dd, *J* = 14.7, 7.7 Hz, 1H), 2.72–2.61 (m, *J* = 12.6, 11.2, 8.8 Hz, 1H), 2.55 (dd, *J* = 14.7, 3.9 Hz, 1H), 2.33–2.14 (m, 1H), 1.22 (d, *J* = 6.6 Hz, 3H); ^13^C NMR (100 MHz, CDCl_3_) δ 175.7, 169.4, 138.7, 138.3, 128.8, 128.7, 125.0, 123.7, 120.1, 119.6, 54.1, 48.1, 37.7, 33.7, 16.6; IR (neat): 3460, 3265, 2932, 2855, 1657 cm^−1^; MS (ESI^+^) *m*/*e* 309.2 [M + H]^+^; HRMS (ESI^+^) calcd for C_19_H_21_N_2_O_2_ [M + H]^+^: 309.1603; found: 309.1590.

**(*S*)-1-Phenyl-3-((*R*)-1-(phenylamino)propan-2-yl)pyrrolidine-2,5-dione** (**7{*1,11*}**): ^1^H NMR (400 MHz, CDCl_3_) δ 7.50–7.33 (m, 3H), 7.24–7.08 (m, 4H), 6.79–6.67 (m, *J* = 7.3 Hz, 1H), 6.67–6.52 (m, *J* = 7.5 Hz, 2H), 3.82 (s, 1H), 3.42–3.26 (m, 1H), 3.26–3.09 (m, 2H), 2.92 (dd, *J* = 18.5, 9.6 Hz, 1H), 2.63 (dd, *J* = 18.5, 5.0 Hz, 1H), 2.63–2.50 (m, *J* = 5.0 Hz, 1H), 1.09 (d, *J* = 7.0 Hz, 3H); ^13^C NMR (100 MHz, CDCl_3_) δ 178.5, 175.6, 147.8, 131.8, 129.3, 129.1, 128.6, 126.6, 117.9, 112.9, 46.9, 42.4, 34.3, 31.3, 15.0; IR (neat): 3380, 2974, 2950, 1775 cm^−1^; MS (ESI^+^) *m*/*e* 309.2 [M + H]^+^; HRMS (ESI^+^) calcd for C_19_H_21_N_2_O_2_ [M + H]^+^: 309.1603; found: 309.1595.

#### General procedure for the combination of the Michael addition, the reductive amination/lactamization, and the epimerization step in a single-pot process to produce γ-lactam **6{*1,1,1*}** (Scheme 4)

A 0.400 M solution of propionaldehyde (**2{*1*}**) in chloroform (1.50 mL, 0.600 mmol, 1.5 equiv) was added to a 0.400 M solution of *N*-phenylmaleimide (**1{*1*}**) in chloroform (1.00 mL, 0.400 mmol, 1.0 equiv) at 0 °C, followed by a 0.100 M solution of (*S*)-(−)-α,α-diphenyl-2-pyrrolidinemethanol trimethylsilyl ether (0.40 mL, 0.040 mmol, 0.10 equiv), and the reaction mixture was stirred at 0 °C for 36 h. A 1.00 M solution of aniline (**4{*1*}**) (0.80 mL, 0.800 mmol, 2.0 equiv) was added followed by sodium triacetoxyborohydride (212 mg, 1.00 mmol, 2.5 equiv) and the reaction mixture was stirred at room temperature for 6 h. The chloroform solvent was removed in vacuo, methanol (10 mL) was added followed by potassium *tert*-butoxide (449 mg, 4.00 mmol, 10.0 equiv), and the reaction mixture was stirred at room temperature for 12 h. The methanol solvent was removed in vacuo, a 4.0 N aqueous solution of hydrochloric acid (5.0 mL, 20 mmol, 50.0 equiv) was added, and the reaction mixture was extracted with methylene chloride (3 × 2 mL). The methylene chloride extracts were combined, dried over sodium sulfate and concentrated in vacuo to yield a yellow liquid. The crude liquid was purified by column chromatography (1/1 ethyl acetate-hexane) to yield 81.4 mg (66%) of a single diastereomer of **6{*1,1,1*}** as a clear colorless liquid. The ee of the product was determined by chiral HPLC analysis to be 80% (Chiralpak AD column, 80/20 hexane/isopropanol).

#### General procedure for the single-pot parallel synthesis of γ-lactams **6** by using an automated synthesizer (Scheme 5)

Using a Chemspeed Accelerator SLT-100 automated synthesizer with 64 individual 13 mL reactor vessels, 0.400 M solutions of the maleimides **1** in chloroform (1.00 mL, 0.400 mmol, 1.0 equiv) were added to the appropriate reactor vessels, and the reactors were cooled to 0 °C. Solutions (0.400 M) of the aldehydes **2** in chloroform (1.50 mL, 0.600 mmol, 1.5 equiv) were added to the appropriate reactor vessels followed by a 0.100 M solution of (*S*)-(−)-α,α-diphenyl-2-pyrrolidinemethanol trimethylsilyl ether (0.40 mL, 0.040 mmol, 0.10 equiv), and the reaction mixtures were subjected to vortex mixing at 0 °C for 36 h. 1.00 M solutions of the amines **4** (0.80 mL, 0.800 mmol, 2.0 equiv) were then added to the appropriate reactor vessels followed by sodium triacetoxyborohydride (212 mg, 1.00 mmol, 2.5 equiv) and the reaction mixtures were subjected to vortex mixing at room temperature for 6 h. The reaction mixtures were concentrated in vacuo within the reactor vessels (30 °C, 6 mmHg), methanol (10 mL) was then added followed by potassium *tert*-butoxide (449 mg, 4.00 mmol, 10.0 equiv), and the reaction mixtures were subjected to vortex mixing at room temperature for 12 h. The reaction mixtures were concentrated in vacuo within the reactor vessels (40 °C, 6 mmHg), followed by the addition of 4.0 N aqueous solution of hydrochloric acid (5.0 mL, 20 mmol, 50.0 equiv) to the resulting residues. The mixtures were extracted with methylene chloride (3 × 2 mL) and each of the extracts were gravity filtered through a silica gel 500 mg SPE-Si cartridge followed by washing the SPE with ethyl acetate (2 × 2 mL). The resulting solutions were then further purified by using preparative HPLC.

## Supporting Information

File 1 Results for the four 4 × 2 × 8 libraries using an automated synthesizer, full characterization data for representative compounds, and copies of ^1^H and ^13^C NMR spectra for representative compounds.

## References

[1] Dandapani S, Marcaurelle L A (2010). Curr Opin Chem Biol.

[2] Shaw J T (2009). Nat Prod Rep.

[3] Spandl R J, Díaz-Gavilán M, O’Connell K M G, Thomas G L, Spring D R (2008). Chem Rec.

[4] Tan D S (2005). Nat Chem Biol.

[5] Schreiber S L (2000). Science.

[6] Reddy P A, Hsiang B C H, Latifi T N, Hill M W, Woodward K E, Rothman S M, Ferrendelli J A, Covey D F (1996). J Med Chem.

[7] Das Sarma K, Zhang J, Huang Y, Davidson J G (2006). Eur J Org Chem.

[8] Spaltenstein A, Almond M R, Bock W J, Cleary D G, Furfine E S, Hazen R J, Kazmierski W M, Salituro F G, Tung R D, Wright L L (2000). Bioorg Med Chem Lett.

[9] Kazmierski W M, Andrews W, Furfine E, Spaltenstein A, Wright L (2004). Bioorg Med Chem Lett.

[10] Barnes D M, Ji J, Fickes M G, Fitzgerald M A, King S A, Morton H E, Plagge F A, Preskill M, Wagaw S H, Wittenberger S J (2002). J Am Chem Soc.

[11] Tang K, Zhang J-T (2002). Neurol Res.

[12] Nöth J, Frankowski K J, Neuenswander B, Aubé J, Reiser O (2008). J Comb Chem.

[13] Zhao G-L, Xu Y, Sundén H, Eriksson L, Sayah M, Córdova A (2007). Chem Commun.

[R14] 14This should ultimately produce lactams with ~80% es.

[R15] 15We observed a decrease in the enantiomeric purities, reflective of the diastereomeric ratio of the succinimide, as we had anticipated (Scheme 3).

[R16] 16The standards for compound purity and quantity are based on those requested from the National Institutes of Health's Molecular Library Small Molecule Repository (http://mlsmr.glpg.com/MLSMR_HomePage/submitcompounds.html).

