# Supplementary material for: Automated three-component synthesis of a library of γ-lactams
Source: Beilstein J Org Chem. 2012 Oct 19;8:1804–13. doi: 10.3762/bjoc.8.206 (PMC3511015; doi:10.3762/bjoc.8.206)

Supporting Information

for

Automated three-component synthesis of a library of γ-lactams

Erik Fenster[1], David Hill[1], Oliver Reiser[2] and Jeffrey Aubé*[1]

Address: [1]Center of Excellence in Chemical Methodologies and Library Development, the University of Kansas, 2034 Becker Drive, Lawrence Kansas, 66047, USA and [2]Institut für Organische Chemie, Universität Regensburg, Universitätsstrasse 31, 93053, Regensburg, Germany

Email: Jeffrey Aubé* - jaube@ku.edu

* Corresponding author

Results for the four 4 × 2 × 8 libraries using an automated synthesizer, full characterization data for representative compounds, and copies of ^{1}H and ^{13}C NMR spectra for representative compounds.

Table of contents:

Results for the four 4 × 2 × 8 libraries using an automated synthesizer S2

Full characterization data for representative compounds S7

Copies of ^{1}H and ^{13}C NMR spectra for representative compounds S17

Results for the four 4 × 2 × 8 libraries using an automated synthesizer (Scheme 5)

Entry	Maleimide	Aldehyde	Amine	Product	Yield (%)[a]	Purity (%)[b]	HRMS, calcd for [M + 1]$^+$	HRMS, found for [M + 1]$^+$
1	**1{*1*}**	**2{*1*}**	**4{*1*}**	**6{*1,1,1*}**	66	100	309.1604	309.1599
2	**1{*1*}**	**2{*1*}**	**4{*2*}**	**6{*1,1,2*}**	73	100	323.1760	323.1756
3	**1{*1*}**	**2{*1*}**	**4{*3*}**	**6{*1,1,3*}**	68	100	337.1917	337.1912
4	**1{*1*}**	**2{*1*}**	**4{*4*}**	**6{*1,1,4*}**	70	100	339.1709	339.1706
5	**1{*1*}**	**2{*1*}**	**4{*5*}**	**6{*1,1,5*}**	33	100	387.0709	387.0699
6	**1{*1*}**	**2{*1*}**	**4{*6*}**	**6{*1,1,6*}**	51	100	315.2073	315.2071
7	**1{*1*}**	**2{*1*}**	**4{*7*}**	**6{*1,1,7*}**	60	100	289.1917	289.1913
8	**1{*1*}**	**2{*1*}**	**4{*8*}**	**6{*1,1,8*}**	0	–	324.1713	–
9	**1{*1*}**	**2{*2*}**	**4{*1*}**	**6{*1,2,1*}**	0	–	371.1760	–
10	**1{*1*}**	**2{*2*}**	**4{*2*}**	**6{*1,2,2*}**	0	–	385.1917	–
11	**1{*1*}**	**2{*2*}**	**4{*3*}**	**6{*1,2,3*}**	9	92	399.2073	399.2063
12	**1{*1*}**	**2{*2*}**	**4{*4*}**	**6{*1,2,4*}**	0	–	401.1866	–
13	**1{*1*}**	**2{*2*}**	**4{*5*}**	**6{*1,2,5*}**	0	–	449.0865	–
14	**1{*1*}**	**2{*2*}**	**4{*6*}**	**6{*1,2,6*}**	9	100	377.2230	377.2223
15	**1{*1*}**	**2{*2*}**	**4{*7*}**	**6{*1,2,7*}**	15	25	351.2073	351.2066
16	**1{*1*}**	**2{*2*}**	**4{*8*}**	**6{*1,2,8*}**	0	–	386.1869	–
17	**1{*2*}**	**2{*1*}**	**4{*1*}**	**6{*2,1,1*}**	6	100	247.1447	247.1441
18	**1{*2*}**	**2{*1*}**	**4{*2*}**	**6{*2,1,2*}**	53	100	261.1604	261.1601
19	**1{*2*}**	**2{*1*}**	**4{*3*}**	**6{*2,1,3*}**	32	100	275.1760	275.1756
20	**1{*2*}**	**2{*1*}**	**4{*4*}**	**6{*2,1,4*}**	18	100	277.1553	277.1550
21	**1{*2*}**	**2{*1*}**	**4{*5*}**	**6{*2,1,5*}**	8	100	325.0552	325.0542
22	**1{*2*}**	**2{*1*}**	**4{*6*}**	**6{*2,1,6*}**	52	100	253.1917	253.1914
23	**1{*2*}**	**2{*1*}**	**4{*7*}**	**6{*2,1,7*}**	51	100	227.1760	227.1757
24	**1{*2*}**	**2{*1*}**	**4{*8*}**	**6{*2,1,8*}**	0	–	262.1556	–
25	**1{*2*}**	**2{*2*}**	**4{*1*}**	**6{*2,2,1*}**	0	–	309.1604	–
26	**1{*2*}**	**2{*2*}**	**4{*2*}**	**6{*2,2,2*}**	4	100	323.1760	323.1751
27	**1{*2*}**	**2{*2*}**	**4{*3*}**	**6{*2,2,3*}**	5	92	337.1917	337.1908
28	**1{*2*}**	**2{*2*}**	**4{*4*}**	**6{*2,2,4*}**	0	–	339.1709	–
29	**1{*2*}**	**2{*2*}**	**4{*5*}**	**6{*2,2,5*}**	0	–	387.0709	–
30	**1{*2*}**	**2{*2*}**	**4{*6*}**	**6{*2,2,6*}**	148	54	315.2073	315.2067
31	**1{*2*}**	**2{*2*}**	**4{*7*}**	**6{*2,2,7*}**	11	92	289.1917	289.1909
32	**1{*2*}**	**2{*2*}**	**4{*8*}**	**6{*2,2,8*}**	0	–	324.1713	–
33	**1{*3*}**	**2{*1*}**	**4{*1*}**	**6{*3,1,1*}**	42	100	339.1709	339.1704
34	**1{*3*}**	**2{*1*}**	**4{*2*}**	**6{*3,1,2*}**	54	100	353.1866	353.1862
35	**1{*3*}**	**2{*1*}**	**4{*3*}**	**6{*3,1,3*}**	38	99	367.2022	367.2018
36	**1{*3*}**	**2{*1*}**	**4{*4*}**	**6{*3,1,4*}**	48	98	369.1815	369.1812
37	**1{*3*}**	**2{*1*}**	**4{*5*}**	**6{*3,1,5*}**	19	100	417.0815	419.0786
38	**1{*3*}**	**2{*1*}**	**4{*6*}**	**6{*3,1,6*}**	51	100	345.2179	345.2176
39	**1{*3*}**	**2{*1*}**	**4{*7*}**	**6{*3,1,7*}**	54	100	319.2022	319.2020
40	**1{*3*}**	**2{*1*}**	**4{*8*}**	**6{*3,1,8*}**	0	–	354.1818	–
41	**1{*3*}**	**2{*2*}**	**4{*1*}**	**6{*3,2,1*}**	0	–	401.1866	–
42	**1{*3*}**	**2{*2*}**	**4{*2*}**	**6{*3,2,2*}**	0	–	415.2022	–
43	**1{*3*}**	**2{*2*}**	**4{*3*}**	**6{*3,2,3*}**	0	–	429.2179	–
44	**1{*3*}**	**2{*2*}**	**4{*4*}**	**6{*3,2,4*}**	0	–	431.1972	–
45	**1{*3*}**	**2{*2*}**	**4{*5*}**	**6{*3,2,5*}**	0	–	479.0971	–
46	**1{*3*}**	**2{*2*}**	**4{*6*}**	**6{*3,2,6*}**	7	96	407.2335	407.2325
47	**1{*3*}**	**2{*2*}**	**4{*7*}**	**6{*3,2,7*}**	0	–	381.2179	–
48	**1{*3*}**	**2{*2*}**	**4{*8*}**	**6{*3,2,8*}**	0	–	416.1975	–

49	**1{*4*}**	**2{*1*}**	**4{*1*}**	**6{*4,1,1*}**	38	100	387.0709	387.0698
50	**1{*4*}**	**2{*1*}**	**4{*2*}**	**6{*4,1,2*}**	0	–	401.0865	–
51	**1{*4*}**	**2{*1*}**	**4{*3*}**	**6{*4,1,3*}**	41	99	415.1022	417.0995
52	**1{*4*}**	**2{*1*}**	**4{*4*}**	**6{*4,1,4*}**	41	100	417.0815	417.0805
53	**1{*4*}**	**2{*1*}**	**4{*5*}**	**6{*4,1,5*}**	41	100	464.9814	466.9782
54	**1{*4*}**	**2{*1*}**	**4{*6*}**	**6{*4,1,6*}**	22	100	393.1178	393.1170
55	**1{*4*}**	**2{*1*}**	**4{*7*}**	**6{*4,1,7*}**	39	75	367.1022	369.0993
56	**1{*4*}**	**2{*1*}**	**4{*8*}**	**6{*4,1,8*}**	0	–	402.0818	–
57	**1{*4*}**	**2{*2*}**	**4{*1*}**	**6{*4,2,1*}**	0	–	449.0865	–
58	**1{*4*}**	**2{*2*}**	**4{*2*}**	**6{*4,2,2*}**	0	–	463.1022	–
59	**1{*4*}**	**2{*2*}**	**4{*3*}**	**6{*4,2,3*}**	1	100	477.1178	479.1147
60	**1{*4*}**	**2{*2*}**	**4{*4*}**	**6{*4,2,4*}**	0	–	479.0971	–
61	**1{*4*}**	**2{*2*}**	**4{*5*}**	**6{*4,2,5*}**	0	–	526.9971	–
62	**1{*4*}**	**2{*2*}**	**4{*6*}**	**6{*4,2,6*}**	5	100	455.1335	457.1305
63	**1{*4*}**	**2{*2*}**	**4{*7*}**	**6{*4,2,7*}**	0	–	429.1178	–
64	**1{*4*}**	**2{*2*}**	**4{*8*}**	**6{*4,2,8*}**	0	–	464.0974	–
65	**1{*1*}**	**2{*3*}**	**4{*1*}**	**6{*1,3,1*}**	66	99	385.1917	385.1911
66	**1{*1*}**	**2{*3*}**	**4{*2*}**	**6{*1,3,2*}**	65	100	384.1838	384.1829
67	**1{*1*}**	**2{*3*}**	**4{*3*}**	**6{*1,3,3*}**	67	100	413.2230	413.2222
68	**1{*1*}**	**2{*3*}**	**4{*4*}**	**6{*1,3,4*}**	69	96	415.2022	415.2016
69	**1{*1*}**	**2{*3*}**	**4{*5*}**	**6{*1,3,5*}**	56	98	463.1022	465.0994
70	**1{*1*}**	**2{*3*}**	**4{*6*}**	**6{*1,3,6*}**	65	100	391.2386	391.2382
71	**1{*1*}**	**2{*3*}**	**4{*7*}**	**6{*1,3,7*}**	65	100	365.2230	365.2224
72	**1{*1*}**	**2{*3*}**	**4{*8*}**	**6{*1,3,8*}**	25	98	400.2026	400.2019
73	**1{*1*}**	**2{*4*}**	**4{*1*}**	**6{*1,4,1*}**	62	97	337.1917	337.1913
74	**1{*1*}**	**2{*4*}**	**4{*2*}**	**6{*1,4,2*}**	65	100	351.2073	351.2068
75	**1{*1*}**	**2{*4*}**	**4{*3*}**	**6{*1,4,3*}**	68	97	365.2230	365.2226
76	**1{*1*}**	**2{*4*}**	**4{*4*}**	**6{*1,4,4*}**	70	100	367.2022	367.2019
77	**1{*1*}**	**2{*4*}**	**4{*5*}**	**6{*1,4,5*}**	46	100	415.1022	417.0994
78	**1{*1*}**	**2{*4*}**	**4{*6*}**	**6{*1,4,6*}**	51	100	343.2386	343.2383
79	**1{*1*}**	**2{*4*}**	**4{*7*}**	**6{*1,4,7*}**	50	97	317.2230	317.2225
80	**1{*1*}**	**2{*4*}**	**4{*8*}**	**6{*1,4,8*}**	2	93	352.2026	352.2017
81	**1{*2*}**	**2{*3*}**	**4{*1*}**	**6{*2,3,1*}**	43	100	323.1760	323.1755
82	**1{*2*}**	**2{*3*}**	**4{*2*}**	**6{*2,3,2*}**	58	100	337.1917	337.1912
83	**1{*2*}**	**2{*3*}**	**4{*3*}**	**6{*2,3,3*}**	37	100	351.2073	351.2067
84	**1{*2*}**	**2{*3*}**	**4{*4*}**	**6{*2,3,4*}**	60	100	353.1866	353.1860
85	**1{*2*}**	**2{*3*}**	**4{*5*}**	**6{*2,3,5*}**	33	100	401.0865	403.0836
86	**1{*2*}**	**2{*3*}**	**4{*6*}**	**6{*2,3,6*}**	58	100	329.2230	329.2225
87	**1{*2*}**	**2{*3*}**	**4{*7*}**	**6{*2,3,7*}**	50	100	303.2073	303.2070
88	**1{*2*}**	**2{*3*}**	**4{*8*}**	**6{*2,3,8*}**	0	–	338.1869	–
89	**1{*2*}**	**2{*4*}**	**4{*1*}**	**6{*2,4,1*}**	32	100	275.1760	275.1756
90	**1{*2*}**	**2{*4*}**	**4{*2*}**	**6{*2,4,2*}**	50	100	289.1917	289.1913
91	**1{*2*}**	**2{*4*}**	**4{*3*}**	**6{*2,4,3*}**	44	100	303.2073	303.2070
92	**1{*2*}**	**2{*4*}**	**4{*4*}**	**6{*2,4,4*}**	55	100	305.1866	305.1861
93	**1{*2*}**	**2{*4*}**	**4{*5*}**	**6{*2,4,5*}**	30	100	353.0865	353.0856
94	**1{*2*}**	**2{*4*}**	**4{*6*}**	**6{*2,4,6*}**	50	100	281.2230	281.2228
95	**1{*2*}**	**2{*4*}**	**4{*7*}**	**6{*2,4,7*}**	40	100	255.2073	255.2072
96	**1{*2*}**	**2{*4*}**	**4{*8*}**	**6{*2,4,8*}**	0	–	290.1869	–
97	**1{*3*}**	**2{*3*}**	**4{*1*}**	**6{*3,3,1*}**	11	100	415.2022	415.2014
98	**1{*3*}**	**2{*3*}**	**4{*2*}**	**6{*3,3,2*}**	62	99	429.2179	429.2172
99	**1{*3*}**	**2{*3*}**	**4{*3*}**	**6{*3,3,3*}**	61	100	443.2335	443.2328
100	**1{*3*}**	**2{*3*}**	**4{*4*}**	**6{*3,3,4*}**	60	100	445.2128	445.2121
101	**1{*3*}**	**2{*3*}**	**4{*5*}**	**6{*3,3,5*}**	20	98	493.1128	495.1096
102	**1{*3*}**	**2{*3*}**	**4{*6*}**	**6{*3,3,6*}**	66	98	314.1994	314.1988
103	**1{*3*}**	**2{*3*}**	**4{*7*}**	**6{*3,3,7*}**	51	97	395.2335	395.2330
104	**1{*3*}**	**2{*3*}**	**4{*8*}**	**6{*3,3,8*}**	20	100	430.2131	430.2123

105	**1{*3*}**	**2{*4*}**	**4{*1*}**	**6{*3,4,1*}**	56	100	367.2022	367.2018
106	**1{*3*}**	**2{*4*}**	**4{*2*}**	**6{*3,4,2*}**	58	98	381.2179	381.2172
107	**1{*3*}**	**2{*4*}**	**4{*3*}**	**6{*3,4,3*}**	47	98	395.2335	395.2332
108	**1{*3*}**	**2{*4*}**	**4{*4*}**	**6{*3,4,4*}**	24	95	397.2128	397.2124
109	**1{*3*}**	**2{*4*}**	**4{*5*}**	**6{*3,4,5*}**	26	95	445.1128	447.1100
110	**1{*3*}**	**2{*4*}**	**4{*6*}**	**6{*3,4,6*}**	51	100	373.2492	373.2488
111	**1{*3*}**	**2{*4*}**	**4{*7*}**	**6{*3,4,7*}**	48	96	347.2335	347.2333
112	**1{*3*}**	**2{*4*}**	**4{*8*}**	**6{*3,4,8*}**	1	83	382.2131	382.2123
113	**1{*4*}**	**2{*3*}**	**4{*1*}**	**6{*4,3,1*}**	41	100	463.1022	465.0991
114	**1{*4*}**	**2{*3*}**	**4{*2*}**	**6{*4,3,2*}**	39	97	477.1178	477.1158
115	**1{*4*}**	**2{*3*}**	**4{*3*}**	**6{*4,3,3*}**	42	91	491.1335	491.1313
116	**1{*4*}**	**2{*3*}**	**4{*4*}**	**6{*4,3,4*}**	43	93	493.1128	493.1120
117	**1{*4*}**	**2{*3*}**	**4{*5*}**	**6{*4,3,5*}**	19	100	541.0127	543.0095
118	**1{*4*}**	**2{*3*}**	**4{*6*}**	**6{*4,3,6*}**	35	96	469.1491	469.1476
119	**1{*4*}**	**2{*3*}**	**4{*7*}**	**6{*4,3,7*}**	41	100	443.1335	443.1317
120	**1{*4*}**	**2{*3*}**	**4{*8*}**	**6{*4,3,8*}**	25	97	478.1131	478.1111
121	**1{*4*}**	**2{*4*}**	**4{*1*}**	**6{*4,4,1*}**	45	92	415.1022	417.0991
122	**1{*4*}**	**2{*4*}**	**4{*2*}**	**6{*4,4,2*}**	33	97	429.1178	429.1154
123	**1{*4*}**	**2{*4*}**	**4{*3*}**	**6{*4,4,3*}**	48	98	443.1335	443.1314
124	**1{*4*}**	**2{*4*}**	**4{*4*}**	**6{*4,4,4*}**	35	97	445.1128	445.1121
125	**1{*4*}**	**2{*4*}**	**4{*5*}**	**6{*4,4,5*}**	16	68	493.0127	495.0095
126	**1{*4*}**	**2{*4*}**	**4{*6*}**	**6{*4,4,6*}**	20	96	421.1491	423.1463
127	**1{*4*}**	**2{*4*}**	**4{*7*}**	**6{*4,4,7*}**	32	100	395.1335	395.1315
128	**1{*4*}**	**2{*4*}**	**4{*8*}**	**6{*4,4,8*}**	26	94	430.1131	430.1116
129	**1{*1*}**	**2{*5*}**	**4{*1*}**	**6{*1,5,1*}**	40	100	355.1481	355.1475
130	**1{*1*}**	**2{*5*}**	**4{*2*}**	**6{*1,5,2*}**	48	100	369.1637	369.1632
131	**1{*1*}**	**2{*5*}**	**4{*3*}**	**6{*1,5,3*}**	36	99	383.1794	383.1790
132	**1{*1*}**	**2{*5*}**	**4{*4*}**	**6{*1,5,4*}**	38	100	385.1587	385.1582
133	**1{*1*}**	**2{*5*}**	**4{*5*}**	**6{*1,5,5*}**	25	100	433.0586	435.0555
134	**1{*1*}**	**2{*5*}**	**4{*6*}**	**6{*1,5,6*}**	29	100	361.1950	361.1945
135	**1{*1*}**	**2{*5*}**	**4{*7*}**	**6{*1,5,7*}**	0	–	335.1794	–
136	**1{*1*}**	**2{*5*}**	**4{*8*}**	**6{*1,5,8*}**	32	100	370.1590	370.1584
137	**1{*1*}**	**2{*6*}**	**4{*1*}**	**6{*1,6,1*}**	6	100	401.1866	401.1859
138	**1{*1*}**	**2{*6*}**	**4{*2*}**	**6{*1,6,2*}**	6	100	415.2022	415.2014
139	**1{*1*}**	**2{*6*}**	**4{*3*}**	**6{*1,6,3*}**	9	98	429.2179	429.2173
140	**1{*1*}**	**2{*6*}**	**4{*4*}**	**6{*1,6,4*}**	9	90	431.1972	431.1960
141	**1{*1*}**	**2{*6*}**	**4{*5*}**	**6{*1,6,5*}**	7	93	479.0971	481.0944
142	**1{*1*}**	**2{*6*}**	**4{*6*}**	**6{*1,6,6*}**	5	88	407.2335	407.2328
143	**1{*1*}**	**2{*6*}**	**4{*7*}**	**6{*1,6,7*}**	3	100	381.2179	381.2171
144	**1{*1*}**	**2{*6*}**	**4{*8*}**	**6{*1,6,8*}**	0	–	416.1975	–
145	**1{*2*}**	**2{*5*}**	**4{*1*}**	**6{*2,5,1*}**	14	100	293.1324	293.1316
146	**1{*2*}**	**2{*5*}**	**4{*2*}**	**6{*2,5,2*}**	31	100	307.1481	307.1477
147	**1{*2*}**	**2{*5*}**	**4{*3*}**	**6{*2,5,3*}**	36	100	321.1637	321.1634
148	**1{*2*}**	**2{*5*}**	**4{*4*}**	**6{*2,5,4*}**	32	100	323.1430	323.1425
149	**1{*2*}**	**2{*5*}**	**4{*5*}**	**6{*2,5,5*}**	29	100	371.0430	373.0401
150	**1{*2*}**	**2{*5*}**	**4{*6*}**	**6{*2,5,6*}**	29	100	299.1794	299.1790
151	**1{*2*}**	**2{*5*}**	**4{*7*}**	**6{*2,5,7*}**	5	100	273.1637	273.1636
152	**1{*2*}**	**2{*5*}**	**4{*8*}**	**6{*2,5,8*}**	0	–	308.1433	–
153	**1{*2*}**	**2{*6*}**	**4{*1*}**	**6{*2,6,1*}**	0	–	339.1709	–
154	**1{*2*}**	**2{*6*}**	**4{*2*}**	**6{*2,6,2*}**	3	100	353.1866	–
155	**1{*2*}**	**2{*6*}**	**4{*3*}**	**6{*2,6,3*}**	2	100	367.2022	367.2017
156	**1{*2*}**	**2{*6*}**	**4{*4*}**	**6{*2,6,4*}**	0	–	369.1815	–
157	**1{*2*}**	**2{*6*}**	**4{*5*}**	**6{*2,6,5*}**	43	100	417.0815	417.0813
158	**1{*2*}**	**2{*6*}**	**4{*6*}**	**6{*2,6,6*}**	68	100	345.2179	345.2171
159	**1{*2*}**	**2{*6*}**	**4{*7*}**	**6{*2,6,7*}**	70	100	319.2022	319.2016
160	**1{*2*}**	**2{*6*}**	**4{*8*}**	**6{*2,6,8*}**	51	97	354.1818	354.1820

161	**1{*3*}**	**2{*5*}**	**4{*1*}**	**6{*3,5,1*}**	36	100	385.1587	385.1580
162	**1{*3*}**	**2{*5*}**	**4{*2*}**	**6{*3,5,2*}**	46	100	399.1743	399.1736
163	**1{*3*}**	**2{*5*}**	**4{*3*}**	**6{*3,5,3*}**	39	100	413.1900	413.1893
164	**1{*3*}**	**2{*5*}**	**4{*4*}**	**6{*3,5,4*}**	2	100	415.1692	415.1686
165	**1{*3*}**	**2{*5*}**	**4{*5*}**	**6{*3,5,5*}**	4	89	463.0692	465.0662
166	**1{*3*}**	**2{*5*}**	**4{*6*}**	**6{*3,5,6*}**	5	90	391.2056	391.2052
167	**1{*3*}**	**2{*5*}**	**4{*7*}**	**6{*3,5,7*}**	7	100	365.1900	365.1896
168	**1{*3*}**	**2{*5*}**	**4{*8*}**	**6{*3,5,8*}**	2	100	400.1696	400.1688
169	**1{*3*}**	**2{*6*}**	**4{*1*}**	**6{*3,6,1*}**	4	89	431.1972	431.1963
170	**1{*3*}**	**2{*6*}**	**4{*2*}**	**6{*3,6,2*}**	5	90	445.2128	445.2116
171	**1{*3*}**	**2{*6*}**	**4{*3*}**	**6{*3,6,3*}**	5	100	459.2285	459.2280
172	**1{*3*}**	**2{*6*}**	**4{*4*}**	**6{*3,6,4*}**	7	72	461.2077	461.2065
173	**1{*3*}**	**2{*6*}**	**4{*5*}**	**6{*3,6,5*}**	4	91	509.1077	511.1048
174	**1{*3*}**	**2{*6*}**	**4{*6*}**	**6{*3,6,6*}**	4	100	437.2441	437.2429
175	**1{*3*}**	**2{*6*}**	**4{*7*}**	**6{*3,6,7*}**	4	100	411.2285	411.2277
176	**1{*3*}**	**2{*6*}**	**4{*8*}**	**6{*3,6,8*}**	0	–	446.2081	–
177	**1{*4*}**	**2{*5*}**	**4{*1*}**	**6{*4,5,1*}**	46	90	433.0586	435.0557
178	**1{*4*}**	**2{*5*}**	**4{*2*}**	**6{*4,5,2*}**	44	100	447.0743	449.0713
179	**1{*4*}**	**2{*5*}**	**4{*3*}**	**6{*4,5,3*}**	45	97	461.0899	463.0867
180	**1{*4*}**	**2{*5*}**	**4{*4*}**	**6{*4,5,4*}**	50	100	463.0692	465.0664
181	**1{*4*}**	**2{*5*}**	**4{*5*}**	**6{*4,5,5*}**	53	100	510.9691	512.9663
182	**1{*4*}**	**2{*5*}**	**4{*6*}**	**6{*4,5,6*}**	40	94	439.1056	441.1025
183	**1{*4*}**	**2{*5*}**	**4{*7*}**	**6{*4,5,7*}**	24	100	413.0899	415.0872
184	**1{*4*}**	**2{*5*}**	**4{*8*}**	**6{*4,5,8*}**	6	95	448.0695	448.0673
185	**1{*4*}**	**2{*6*}**	**4{*1*}**	**6{*4,6,1*}**	13	82	479.0971	481.0941
186	**1{*4*}**	**2{*6*}**	**4{*2*}**	**6{*4,6,2*}**	10	84	493.1128	495.1099
187	**1{*4*}**	**2{*6*}**	**4{*3*}**	**6{*4,6,3*}**	10	98	507.1284	509.1255
188	**1{*4*}**	**2{*6*}**	**4{*4*}**	**6{*4,6,4*}**	12	97	509.1077	511.1050
189	**1{*4*}**	**2{*6*}**	**4{*5*}**	**6{*4,6,5*}**	13	93	557.0076	559.0045
190	**1{*4*}**	**2{*6*}**	**4{*6*}**	**6{*4,6,6*}**	7	100	485.1441	487.1412
191	**1{*4*}**	**2{*6*}**	**4{*7*}**	**6{*4,6,7*}**	9	100	459.1284	461.1257
192	**1{*4*}**	**2{*6*}**	**4{*8*}**	**6{*4,6,8*}**	2	83	494.1080	494.1069
193	**1{*1*}**	**2{*7*}**	**4{*1*}**	**6{*1,7,1*}**	0	–	449.0865	–
194	**1{*1*}**	**2{*7*}**	**4{*2*}**	**6{*1,7,2*}**	2	96	463.1022	465.0994
195	**1{*1*}**	**2{*7*}**	**4{*3*}**	**6{*1,7,3*}**	2	88	477.1178	479.1149
196	**1{*1*}**	**2{*7*}**	**4{*4*}**	**6{*1,7,4*}**	3	58	479.0971	481.0945
197	**1{*1*}**	**2{*7*}**	**4{*5*}**	**6{*1,7,5*}**	0	100	526.9971	528.9939
198	**1{*1*}**	**2{*7*}**	**4{*6*}**	**6{*1,7,6*}**	2	65	455.1335	457.1305
199	**1{*1*}**	**2{*7*}**	**4{*7*}**	**6{*1,7,7*}**	3	64	429.1178	431.1151
200	**1{*1*}**	**2{*7*}**	**4{*8*}**	**6{*1,7,8*}**	4	4	464.0974	464.0956
201	**1{*1*}**	**2{*8*}**	**4{*1*}**	**6{*1,8,1*}**	16	96	323.1760	323.1757
202	**1{*1*}**	**2{*8*}**	**4{*2*}**	**6{*1,8,2*}**	18	100	337.1917	337.1915
203	**1{*1*}**	**2{*8*}**	**4{*3*}**	**6{*1,8,3*}**	15	100	351.2073	351.2068
204	**1{*1*}**	**2{*8*}**	**4{*4*}**	**6{*1,8,4*}**	18	98	353.1866	353.1863
205	**1{*1*}**	**2{*8*}**	**4{*5*}**	**6{*1,8,5*}**	14	100	401.0865	403.0839
206	**1{*1*}**	**2{*8*}**	**4{*6*}**	**6{*1,8,6*}**	15	100	329.2230	329.2226
207	**1{*1*}**	**2{*8*}**	**4{*7*}**	**6{*1,8,7*}**	19	100	303.2073	303.2071
208	**1{*1*}**	**2{*8*}**	**4{*8*}**	**6{*1,8,8*}**	3	100	338.1869	338.1864
209	**1{*2*}**	**2{*7*}**	**4{*1*}**	**6{*2,7,1*}**	0	–	387.0709	–
210	**1{*2*}**	**2{*7*}**	**4{*2*}**	**6{*2,7,2*}**	2	51	401.0865	401.0855
211	**1{*2*}**	**2{*7*}**	**4{*3*}**	**6{*2,7,3*}**	1	75	415.1022	417.0996
212	**1{*2*}**	**2{*7*}**	**4{*4*}**	**6{*2,7,4*}**	1	64	417.0815	417.0814
213	**1{*2*}**	**2{*7*}**	**4{*5*}**	**6{*2,7,5*}**	0	–	464.9814	–
214	**1{*2*}**	**2{*7*}**	**4{*6*}**	**6{*2,7,6*}**	7	96	393.1178	395.1151
215	**1{*2*}**	**2{*7*}**	**4{*7*}**	**6{*2,7,7*}**	4	57	367.1022	367.1013
216	**1{*2*}**	**2{*7*}**	**4{*8*}**	**6{*2,7,8*}**	0	–	402.0818	–

217	**1{*2*}**	**2{*8*}**	**4{*1*}**	**6{*2,8,1*}**	4	100	261.1604	261.1599
218	**1{*2*}**	**2{*8*}**	**4{*2*}**	**6{*2,8,2*}**	6	100	275.1760	275.1755
219	**1{*2*}**	**2{*8*}**	**4{*3*}**	**6{*2,8,3*}**	7	100	289.1917	289.1912
220	**1{*2*}**	**2{*8*}**	**4{*4*}**	**6{*2,8,4*}**	6	100	291.1709	291.1704
221	**1{*2*}**	**2{*8*}**	**4{*5*}**	**6{*2,8,5*}**	4	100	339.0709	339.0699
222	**1{*2*}**	**2{*8*}**	**4{*6*}**	**6{*2,8,6*}**	9	100	267.2073	267.2071
223	**1{*2*}**	**2{*8*}**	**4{*7*}**	**6{*2,8,7*}**	6	100	241.1917	241.1912
224	**1{*2*}**	**2{*8*}**	**4{*8*}**	**6{*2,8,8*}**	2	44	276.1713	276.1706
225	**1{*3*}**	**2{*7*}**	**4{*1*}**	**6{*3,7,1*}**	0	–	479.0971	–
226	**1{*3*}**	**2{*7*}**	**4{*2*}**	**6{*3,7,2*}**	2	64	493.1128	495.1098
227	**1{*3*}**	**2{*7*}**	**4{*3*}**	**6{*3,7,3*}**	3	37	507.1284	509.1254
228	**1{*3*}**	**2{*7*}**	**4{*4*}**	**6{*3,7,4*}**	3	89	509.1077	511.1052
229	**1{*3*}**	**2{*7*}**	**4{*5*}**	**6{*3,7,5*}**	0	–	557.0076	–
230	**1{*3*}**	**2{*7*}**	**4{*6*}**	**6{*3,7,6*}**	4	38	485.1441	487.1413
231	**1{*3*}**	**2{*7*}**	**4{*7*}**	**6{*3,7,7*}**	2	70	459.1284	461.1255
232	**1{*3*}**	**2{*7*}**	**4{*8*}**	**6{*3,7,8*}**	0	–	494.1080	–
233	**1{*3*}**	**2{*8*}**	**4{*1*}**	**6{*3,8,1*}**	10	98	353.1866	353.1860
234	**1{*3*}**	**2{*8*}**	**4{*2*}**	**6{*3,8,2*}**	17	97	367.2022	367.2020
235	**1{*3*}**	**2{*8*}**	**4{*3*}**	**6{*3,8,3*}**	0	–	381.2179	–
236	**1{*3*}**	**2{*8*}**	**4{*4*}**	**6{*3,8,4*}**	10	89	383.1972	383.1964
237	**1{*3*}**	**2{*8*}**	**4{*5*}**	**6{*3,8,5*}**	7	47	431.0971	433.0944
238	**1{*3*}**	**2{*8*}**	**4{*6*}**	**6{*3,8,6*}**	1	–	359.2335	–
239	**1{*3*}**	**2{*8*}**	**4{*7*}**	**6{*3,8,7*}**	9	78	333.2179	333.2173
240	**1{*3*}**	**2{*8*}**	**4{*8*}**	**6{*3,8,8*}**	0	100	368.1975	368.1973
241	**1{*4*}**	**2{*7*}**	**4{*1*}**	**6{*4,7,1*}**	3	83	526.9971	528.9940
242	**1{*4*}**	**2{*7*}**	**4{*2*}**	**6{*4,7,2*}**	5	90	541.0127	543.0094
243	**1{*4*}**	**2{*7*}**	**4{*3*}**	**6{*4,7,3*}**	4	72	555.0284	557.0255
244	**1{*4*}**	**2{*7*}**	**4{*4*}**	**6{*4,7,4*}**	5	93	557.0076	559.0049
245	**1{*4*}**	**2{*7*}**	**4{*5*}**	**6{*4,7,5*}**	3	87	604.9076	608.9029
246	**1{*4*}**	**2{*7*}**	**4{*6*}**	**6{*4,7,6*}**	6	90	533.0440	535.0408
247	**1{*4*}**	**2{*7*}**	**4{*7*}**	**6{*4,7,7*}**	10	100	507.0284	509.0250
248	**1{*4*}**	**2{*7*}**	**4{*8*}**	**6{*4,7,8*}**	3	64	542.0080	544.0050
249	**1{*4*}**	**2{*8*}**	**4{*1*}**	**6{*4,8,1*}**	28	100	401.0865	401.0855
250	**1{*4*}**	**2{*8*}**	**4{*2*}**	**6{*4,8,2*}**	19	100	415.1022	415.1010
251	**1{*4*}**	**2{*8*}**	**4{*3*}**	**6{*4,8,3*}**	22	100	429.1178	431.1150
252	**1{*4*}**	**2{*8*}**	**4{*4*}**	**6{*4,8,4*}**	28	100	431.0971	431.0963
253	**1{*4*}**	**2{*8*}**	**4{*5*}**	**6{*4,8,5*}**	15	25	478.9971	480.9941
254	**1{*4*}**	**2{*8*}**	**4{*6*}**	**6{*4,8,6*}**	12	100	407.1335	407.1323
255	**1{*4*}**	**2{*8*}**	**4{*7*}**	**6{*4,8,7*}**	18	100	381.1178	381.1168
256	**1{*4*}**	**2{*8*}**	**4{*8*}**	**6{*4,8,8*}**	17	100	416.0974	416.0963

[a]Purified by an automated preparative reverse-phase HPLC (Waters 2767 Mass Directed Fractionation) detected by UV (270 nm, Waters Xterra MS C-18 column, 19 × 150 mm, 5 μm elution with the appropriate gradient of acetonitrile in pH 9.8 buffered aqueous ammonium formate at 18 mL·min^{-1} flow rate). [b]Purity was determined by reverse-phase HPLC (Waters Acquity system) with peak area (UV) at 214 nm. (Waters Acquity BEH C-18 column, 2.1 × 50 mm, 1.7 μm elution with a linear gradient of 5% acetonitrile in pH 9.8 buffered aqueous ammonium formate to 100% acetonitrile at 0.6 mL·min^{-1} flow rate)

Full characterization data for representative compounds

General information: Full compound characterizations for a representative set of 20 γ-lactams resulting from the four 4 × 2 × 8 libraries shown in Scheme 5 in the main manuscript are reported.

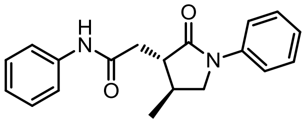

2-((3*S*,4*S*)-4-Methyl-2-oxo-1-phenylpyrrolidin-3-yl)-*N*-phenylacetamide (6{*1,1,1*}). White solid; mp 97–100 °C; yield 81.7 mg (66%); purity 100%; $[\alpha]_D^{26}$ −17.0 (*c* 0.44, $CHCl_3$); IR (neat): 3313, 3047, 2964, 2878, 1669 cm^{-1}; ^1H NMR (500 MHz, $CDCl_3$) δ 9.60 (s, 1H), 7.57 (m, 4H), 7.41–7.34 (m, 2H), 7.30–7.24 (m, 2H), 7.20–7.14 (m, 1H), 7.08–7.02 (m, 1H), 3.83 (dd, *J* = 9.5, 7.9 Hz, 1H), 3.46 (t, *J* = 9.5 Hz, 1H), 2.87 (dd, *J* = 14.7, 7.8 Hz, 1H), 2.71–2.64 (m, 1H), 2.60–2.55 (m, 1H), 2.32–2.21 (m, 1H), 1.25 (d, *J* = 6.6 Hz, 3H); ^{13}C NMR (125 MHz, $CDCl_3$) δ 175.9, 169.5, 138.8, 138.4, 129.0, 128.8, 125.1, 123.9, 120.2, 119.8, 54.3, 48.2, 37.9, 33.8, 16.8; MS (ESI) *m*/*e* 309.2 $[M + 1]^+$; HRMS calcd for $C_{19}H_{20}N_2O_2$ $[M + 1]^+$: 309.1604; found: 309.1599.

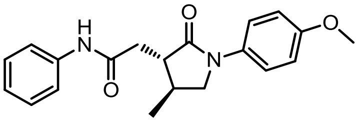

2-((3*S*,4*S*)-1-(4-Methoxyphenyl)-4-methyl-2-oxopyrrolidin-3-yl)-*N*-phenylacetamide (6{*1,1,4*}). White solid; mp 133–134 °C; yield 94.7 mg (70%); purity 100%; $[\alpha]_D^{24}$ −7.4 (*c* 0.49, $CHCl_3$); IR (neat): 3460, 3311, 2960, 2836, 1664 cm^{-1}; ^1H NMR (500 MHz, $CDCl_3$) δ 9.77 (s, 1H), 7.63–7.53 (m, 2H), 7.49–7.45 (m, 2H), 7.30–7.27 (m, 2H), 7.08–7.03 (m, 1H), 6.94–6.89 (m, 2H), 3.83–3.77 (m, 4H), 3.47 (m, 1H), 2.92–2.82 (m, 1H), 2.67 (ddd, *J* = 11.2, 8.5, 3.1 Hz, 1H), 2.61–2.56 (m, 1H), 2.33–2.22 (m, 1H), 1.27 (d, *J* = 6.6 Hz, 3H); ^{13}C NMR (125 MHz, $CDCl_3$) δ 175.7, 169.5, 157.1, 138.5, 131.8, 128.8, 123.8, 122.2, 119.7, 114.2, 55.5, 54.8, 48.1, 38.2, 34.1, 16.7; MS (ESI) *m*/*e* 339.2 $[M + 1]^+$; HRMS calcd for $C_{20}H_{22}N_2O_3$ $[M + 1]^+$: 339.1709; found: 339.1706.

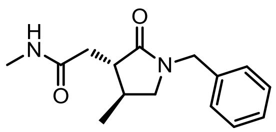

2-((3*S*,4*S*)-1-Benzyl-4-methyl-2-oxopyrrolidin-3-yl)-*N*-methylacetamide (6{*2,1,2*}). Clear colorless liquid; yield 55.4 mg (53%); purity 100%; $[\alpha]_D^{25}$ −3.1 (*c* 0.25, $CHCl_3$); IR (neat): 3313, 3091, 2964, 2928, 1656 cm^{-1}; ^1H NMR (500 MHz, $CDCl_3$) δ 7.41 (s, 1H), 7.36–7.27 (m, 3H), 7.23–7.18 (m, 2H), 4.55–4.34 (m, 2H), 3.29 (dd, *J* = 9.7, 8.0 Hz, 1H), 2.88–2.83 (m, 1H), 2.79 (d, *J* = 4.8 Hz, 3H), 2.58 (dd, *J* = 14.6, 8.0 Hz, 1H), 2.46 (dd, *J* = 14.6, 3.7 Hz, 1H), 2.44–2.37 (m, 1H), 2.15–2.06 (m, 1H), 1.12 (d, *J* = 6.6 Hz, 3H); ^{13}C NMR (125 MHz, $CDCl_3$) δ 176.3, 171.9, 136.0, 128.8, 126.0, 127.8, 52.3, 47.1, 46.7, 36.6, 34.0, 26.3, 17.0; MS (ESI) *m*/*e* 261.2 $[M + 1]^+$; HRMS calcd for $C_{15}H_{20}N_2O_2$ $[M + 1]^+$: 261.1604; found: 261.1601.

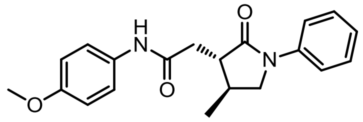

***N*-(4-Methoxyphenyl)-2-((3*S*,4*S*)-4-methyl-2-oxo-1-phenylpyrrolidin-3-yl)acetamide (6{*3,1,1*})**. White solid; mp 131–134 °C; yield 56.5 mg (42%); purity 100%; $[\alpha]_D^{24}$ −12.2 (*c* 0.40, $CHCl_3$); IR (neat): 3306, 3064, 2960, 2934, 1673 cm^{-1}; ^1H NMR (500 MHz, $CDCl_3$) δ 9.77 (s, 1H), 7.63–7.53 (m, 2H), 7.49–7.45 (m, 2H), 7.30–7.27 (m, 2H), 7.08–7.03 (m, 1H), 6.94–6.89 (m, 2H), 3.83–3.77 (m, 4H), 3.47 (m, 1H), 2.92–2.82 (m, 1H), 2.67 (ddd, *J* = 11.2, 8.5, 3.1 Hz, 1H), 2.61–2.56 (m, 1H), 2.33–2.22 (m, 1H), 1.27 (d, *J* = 6.6 Hz, 3H); ^{13}C NMR (125 MHz, $CDCl_3$) δ 175.7, 169.5, 157.1, 138.5, 131.8, 128.8, 123.8, 122.2, 119.7, 114.2, 55.5, 54.8, 48.1, 38.2, 34.1, 16.7; MS (ESI) *m*/*e* 339.2 $[M + 1]^+$; HRMS calcd for $C_{20}H_{22}N_2O_3$ $[M + 1]^+$: 339.1709; found: 339.1704.

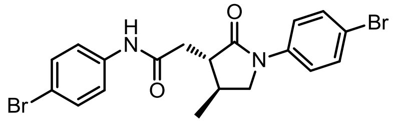

***N*-(4-Bromophenyl)-2-((3*S*,4*S*)-1-(4-bromophenyl)-4-methyl-2-oxopyrrolidin-3-yl)acetamide (6{*4,1,5*})**. White solid; mp 149–154 °C; yield 76.3 mg (41%); purity 100%; $[\alpha]_D^{25}$ 21.2 (*c* 0.26, $CHCl_3$); IR (neat): 3426, 3299, 2967, 2881, 1702, 1672 cm^{-1}; ^1H NMR (500 MHz, $CDCl_3$) δ 9.68 (s, 1H), 7.50 (s, 4H), 7.49–7.45 (m, 2H), 7.41–7.37 (m, 2H), 3.83 (dt, *J* = 13.0, 6.5 Hz, 1H), 3.47 (t, *J* = 9.5 Hz, 1H), 2.85 (dd, *J* = 14.8, 8.5 Hz, 1H), 2.71–2.65 (m, 1H), 2.60–

2.55 (m, 1H), 2.33–2.22 (m, 1H), 1.29 (t, *J* = 6.9 Hz, 3H); ^{13}C NMR (125 MHz, $CDCl_3$) δ 176.1, 169.3, 137.7, 137.5, 132.0, 131.8, 121.6, 121.2, 118.1, 116.3, 54.2, 48.1, 41.0, 37.8, 33.8, 16.6; MS (ESI) *m*/*e* 467.0 $[M + 1]^+$; HRMS calcd for $C_{19}H_{18}Br_2N_2O_2$ $[M + 1]^+$: 466.9788; found: 466.9782.

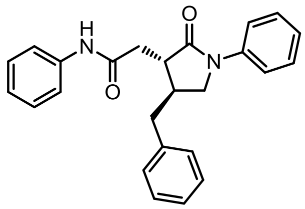

2-((3*S*,4*S*)-4-Benzyl-2-oxo-1-phenylpyrrolidin-3-yl)-*N*-phenylacetamide (6{*1,3,2*}). Clear colorless liquid; yield 103.6 mg (65%); purity 100%; $[\alpha]_D^{26}$ −3.5 (*c* 0.60, $CHCl_3$); IR (neat): 3313, 3273, 3031, 2924, 2861, 1666 cm^{-1}; ^{1}H NMR (500 MHz, $CDCl_3$) δ 9.67 (s, 1H), 7.58 (dd, *J* = 8.5, 1.0 Hz, 2H), 7.32–7.23 (m, 7H), 7.21–7.13 (m, 3H), 7.11–7.04 (m, 3H), 4.45 (m, 2H), 3.13 (dd, *J* = 10.0, 8.0 Hz, 1H), 3.04 (dd, *J* = 13.7, 4.8 Hz, 1H), 3.01–2.97 (m, 1H), 2.78–2.68 (m, 2H), 2.67–2.62 (m, 1H), 2.59–2.55 (m, 1H), 2.49–2.36 (m, 1H); ^{13}C NMR (125 MHz, $CDCl_3$) δ 176.1, 169.3, 138.5, 138.4, 135.7, 128.8, 128.8, 128.7, 128.7, 127.9, 127.8, 126.6, 123.8, 119.7, 50.5, 46.8, 45.3, 40.3, 38.7, 38.1; MS (ESI) *m*/*e* 399.2 $[M + 1]^+$; HRMS calcd for $C_{25}H_{24}N_2O_2$ $[M + 1]^+$: 384.1838; found: 384.1829.

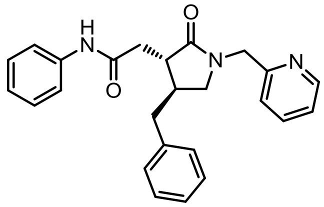

2-((3*S*,4*S*)-4-Benzyl-2-oxo-1-(pyridin-2-ylmethyl)pyrrolidin-3-yl)-*N*-phenylacetamide (6{*1,3,8*}). Colorless liquid; yield 40.4 mg (25%); purity 98%; $[\alpha]_D^{31}$ 4.8 (*c* 0.24, $CHCl_3$); IR (neat): 3313, 3270, 3034, 2921, 2858, 1672 cm^{-1}; ^{1}H NMR (500 MHz, $CDCl_3$) δ 9.36 (s, 1H), 8.53 (dd, *J* = 4.8, 1.4 Hz, 1H), 8.46 (d, *J* = 1.7 Hz, 1H), 7.59–7.54 (m, 2H), 7.50 (dt, *J* = 7.8, 1.8 Hz, 1H), 7.34–7.27 (m, 4H), 7.24–7.15 (m, 2H), 7.10 (ddd, *J* = 7.4, 5.6, 1.2 Hz, 3H), 4.56–4.36 (m, 2H), 3.15 (dd, *J* = 9.8, 8.0 Hz, 1H), 3.06 (dd, *J* = 13.7, 4.9 Hz, 1H), 3.04–3.00 (m, 1H), 2.73–2.66 (m, 3H), 2.65–2.58 (m, 1H), 2.52–2.40 (m, 1H); ^{13}C NMR (125 MHz, $CDCl_3$) δ 176.3, 169.0, 149.4, 149.3, 138.3, 138.2, 135.7, 131.5, 128.9, 128.8, 128.7, 126.8, 124.0, 123.8, 119.6,

50.6, 45.1, 44.4, 40.3, 38.7, 38.0; MS (ESI) *m*/*e* 399.2 [M + 1]$^+$; HRMS calcd for $C_{25}H_{25}N_3O_2$ [M + 1]$^+$: 400.2026; found: 400.2019.

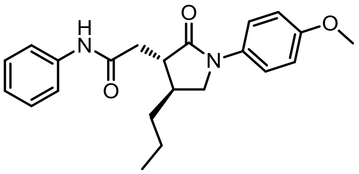

2-((3*S*,4*S*)-1-(4-Methoxyphenyl)-2-oxo-4-propylpyrrolidin-3-yl)-*N*-phenylacetamide (6{*1,4,4*}). Clear yellow liquid; yield 102.9 mg (70%); purity 100%; $[\alpha]_D^{31}$ 19.1 (*c* 0.53, $CHCl_3$); IR (neat): 3313, 3270, 3057, 2954, 2928, 2868, 1670 cm^{-1}; ^{1}H NMR (500 MHz, $CDCl_3$) δ 9.76 (s, 1H), 7.61–7.54 (m, 2H), 7.49–7.45 (m, 2H), 7.31–7.26 (m, 2H), 7.08–7.03 (m, 1H), 6.94–6.90 (m, 2H), 3.83 (dd, *J* = 9.6, 8.1 Hz, 1H), 3.81 (s, 3H), 3.49 (t, *J* = 9.4 Hz, 1H), 2.90–2.83 (m, 1H), 2.72 (ddd, *J* = 11.1, 8.5, 2.8 Hz, 1H), 2.63–2.59 (m, 1H), 2.27–2.18 (m, 1H), 1.86–1.75 (m, 1H), 1.50–1.34 (m, 3H), 0.97 (t, *J* = 7.2 Hz, 3H); ^{13}C NMR (125 MHz, $CDCl_3$) δ 175.8, 169.5, 157.1, 138.5, 131.9, 128.8, 123.8, 122.3, 119.7, 114.2, 55.5, 53.6, 46.8, 38.6, 38.6, 34.9, 20.5, 14.2; MS (ESI) *m*/*e* 367.2 [M + 1]$^+$; HRMS calcd for $C_{22}H_{26}N_2O_3$ [M + 1]$^+$: 367.2022; found: 367.2019.

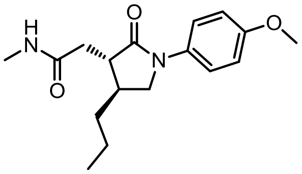

2-((3*S*,4*S*)-1-(4-Methoxyphenyl)-2-oxo-4-propylpyrrolidin-3-yl)-*N*-methylacetamide (6{*2,4,4*}). Clear yellow liquid; yield 67.5 mg (55%); purity 100%; $[\alpha]_D^{27}$ 7.2 (*c* 0.14, $CHCl_3$); IR (neat): 3310, 3094, 2958, 2934, 2874, 1652 cm^{-1}; ^{1}H NMR (500 MHz, $CDCl_3$) δ 7.46 (d, 2H), 7.25 (s, 1H), 6.91 (d, 2H), 3.85–3.80 (m, 1H), 3.80 (s, 3H), 3.45 (t, *J* = 9.2 Hz, 1H), 2.78 (d, *J* = 4.8 Hz, 3H), 2.69–2.58 (m, 2H), 2.53–2.47 (m, 1H), 2.25–2.14 (m, 1H), 1.80–1.71 (m, 1H), 1.49–1.30 (m, 3H), 0.96 (t, 3H); ^{13}C NMR (125 MHz, $CDCl_3$) δ 175.5, 171.9, 156.9, 132.1, 122.0, 114.1, 55.5, 53.4, 46.8, 38.3, 37.0, 35.0, 26.3, 20.4, 14.2; MS (ESI) *m*/*e* 305.2 [M + 1]$^+$; HRMS calcd for $C_{17}H_{24}N_2O_3$ [M + 1]$^+$: 305.1866; found: 305.1861.

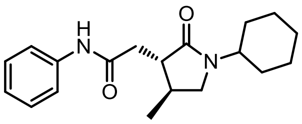

2-((3*S*,4*S*)-1-Cyclohexyl-4-methyl-2-oxopyrrolidin-3-yl)-*N*-phenylacetamide (6{*3,3,6*}). Yellow solid; yield 110.4 mg (66%); purity 98%; $[\alpha]_D^{26}$ −3.1 (*c* 0.22, $CHCl_3$); IR (neat): 3460, 3265, 2932, 2855, 1657 cm^{-1}; ^1H NMR (500 MHz, $CDCl_3$) δ 9.92 (s, 1H), 7.54–7.49 (m, 2H), 7.35–7.29 (m, 2H), 7.27–7.21 (m, 1H), 7.13 (d, *J* = 7.1 Hz, 2H), 6.87–6.82 (m, 2H), 4.01–3.89 (m, 1H), 3.78 (s, 3H), 3.18–3.10 (m, 2H), 3.04–2.97 (m, 1H), 2.94–2.83 (m, 2H), 2.81–2.71 (m, 1H), 2.59–2.52 (m, 1H), 2.27–2.19 (m, 1H), 1.86–1.63 (m, 5H), 1.43–1.28 (m, 4H), 1.11–1.01 (m, 1H); ^{13}C NMR (125 MHz, $CDCl_3$) δ 175.1, 169.9, 155.9, 139.2, 131.9, 129.0, 128.7, 128.7, 126.6, 121.4, 121.3, 114.0, 114.0, 55.5, 55.5, 50.9, 45.0, 43. 8, 40.9, 38.3, 35.0, 33.7, 30.3, 25.4, 25.3; MS (ESI) *m*/*e* 421.2 $[M + 1]^+$; HRMS calcd for $C_{19}H_{26}N_2O_2$ $[M + 1]^+$: 314.1994; found: 314.1988.

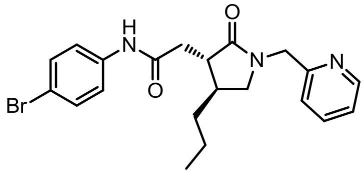

***N*-(4-Bromophenyl)-2-((3*S*,4*S*)-2-oxo-4-propyl-1-(pyridin-2-ylmethyl)pyrrolidin-3-yl)acetamide (6{*4,4,8*})**. Clear colorless liquid; yield 44.2 mg (26%); purity 94%; $[\alpha]_D^{27}$ 2.1 (*c* 0.50, $CHCl_3$); IR (neat): 3480, 3264, 2958, 2929, 1666 cm^{-1}; ^1H NMR (500 MHz, $CDCl_3$) δ 10.07 (s, 1H), 8.57 (dd, *J* = 4.8, 1.5 Hz, 1H), 8.52 (d, *J* = 1.9 Hz, 1H), 7.55 (dt, *J* = 7.8, 1.9 Hz, 1H), 7.51–7.47 (m, 2H), 7.43–7.38 (m, 2H), 7.25 (ddd, *J* = 7.8, 4.8, 0.6 Hz, 1H), 4.51 (m, 2H), 3.39–3.32 (m, 1H), 2.95 (t, *J* = 9.3 Hz, 1H), 2.79–2.73 (m, 1H), 2.61–2.53 (m, 2H), 2.13–2.03 (m, 1H), 1.74–1.64 (m, 1H), 1.39–1.22 (m, 3H), 0.94–0.88 (m, 3H); ^{13}C NMR (125 MHz, $CDCl_3$) δ 176.9, 169.4, 149.5, 149.4, 137.7, 135.7, 131.8, 131.4, 123.9, 121.1, 116.2, 51.2, 45.4, 44.5, 38.9, 38.6, 35.0, 20.4, 14.1; MS (ESI) *m*/*e* 430.1 $[M + 1]^+$; HRMS calcd for $C_{21}H_{24}BrN_3O_2$ $[M + 1]^+$: 430.1131; found: 430.1116.

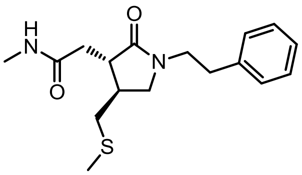

***N*-Methyl-2-((*3S,4S*)-4-((methylthio)methyl)-2-oxo-1-phenethylpyrrolidin-3-yl)acetamide (6{*2,5,3*})**. Clear colorless liquid; yield 45.8 mg (36%); purity 100%; $[\alpha]_D^{25}$ −15.3 (*c* 0.54, $CHCl_3$); IR (neat): 3310, 3091, 2918, 2868, 1656 cm^{-1}; ^1H NMR (500 MHz, $CDCl_3$) δ 7.34–7.28 (m, 2H), 7.25–7.18 (m, 3H), 7.07 (s, 1H), 3.53 (dd, *J* = 13.7, 6.1 Hz, 2H), 3.37 (dd, *J* = 9.9, 8.0 Hz, 1H), 3.01 (dd, *J* = 9.9, 7.9 Hz, 1H), 2.87–2.83 (m, 2H), 2.80–2.78 (m, 1H), 2.77 (d, *J* = 4.8 Hz, 3H), 2.58–2.48 (m, 2H), 2.46–2.37 (m, 2H), 2.32–2.24 (m, 1H), 2.09 (s, 3H); ^{13}C NMR (125 MHz, $CDCl_3$) δ 175.3, 171.6, 138.3, 128.7, 128.6, 126.7, 51.2, 44.9, 44.2, 38.1, 37.2, 37.0, 33.6, 26.3, 15.9; MS (ESI) *m/e* 321.2 $[M + 1]^+$; HRMS calcd for $C_{17}H_{24}N_2O_2S$ $[M + 1]^+$: 321.1637; found: 321.1634.

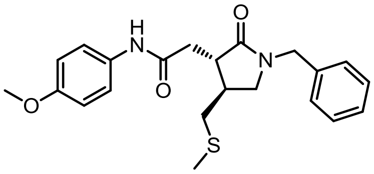

2-((3*S*,4*S*)-1-Benzyl-4-((methylthio)methyl)-2-oxopyrrolidin-3-yl)-*N*-(4-methoxyphenyl)acetamide (6{*3,5,2*}). Clear yellow liquid; yield 72.6 mg (46%); purity 100%; $[\alpha]_D^{27}$ 7.8 (*c* 0.24, $CHCl_3$); IR (neat): 3303, 3067, 2915, 2841, 1666 cm^{-1}; ^1H NMR (500 MHz, $CDCl_3$) δ 9.36 (s, 1H), 7.49–7.46 (m, 2H), 7.31–7.27 (m, 3H), 7.21–7.18 (m, *J* = 5.6, 2.1 Hz, 2H), 6.86–6.83 (m, 2H), 4.60–4.37 (m, 2H), 3.79 (s, 3H), 3.42 (dd, *J* = 10.1, 8.1 Hz, 1H), 3.04 (dd, *J* = 10.0, 8.4 Hz, 1H), 2.87–2.81 (m, 2H), 2.76–2.70 (m, 1H), 2.66–2.62 (m, 1H), 2.55–2.49 (m, 1H), 2.46–2.38 (m, 1H), 2.09 (s, 3H); ^{13}C NMR (125 MHz, $CDCl_3$) δ 175.6, 168.87, 156.1, 135.6, 131.6, 128.7, 128.0, 127.9, 121.4, 114.0, 55.5, 50.4, 46.9, 45.0, 38.1, 38.0, 37.1, 16.0; MS (ESI) *m/e* 399.2 $[M + 1]^+$; HRMS calcd for $C_{22}H_{26}N_2O_3S$ $[M + 1]^+$: 399.1743; found: 399.1736.

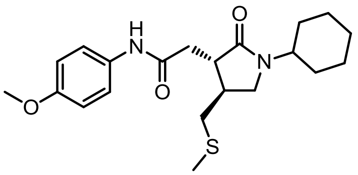

2-((3*S*,4*S*)-1-Cyclohexyl-4-((methylthio)methyl)-2-oxopyrrolidin-3-yl)-*N*-(4-methoxyphenyl)acetamide (6{*3,5,6*}). Pale yellow solid; mp 139–143 °C; yield 8.5 mg (5%); purity 90%; $[\alpha]_D^{28}$ 2.5 (*c* 0.40, $CHCl_3$); IR (neat): 3460, 3267, 2932, 2856, 1656 cm^{-1}; ^1H NMR (500 MHz, $CDCl_3$) δ 9.73 (s, 1H), 7.51–7.41 (m, 2H), 6.88–6.76 (m, 2H), 4.02–3.90 (m, 1H), 3.78 (s, 3H), 3.55 (dd, *J* = 10.0, 8.0 Hz, 1H), 3.06 (dd, *J* = 9.9, 8.5 Hz, 1H), 2.88 (dd, *J* = 13.0, 4.4 Hz, 1H), 2.82–2.76 (m, 1H), 2.70–2.64 (m, 1H), 2.56–2.51 (m, 2H), 2.36–2.27 (m, 1H), 2.13 (s, 3H), 1.93–1.62 (m, 5H), 1.45–1.32 (m, 4H), 1.15–1.06 (m, 1H); ^{13}C NMR (125 MHz, $CDCl_3$) δ 175.0, 169.1, 156.0, 131.8, 121.3, 114.0, 55. 5, 51.1, 46.9, 45.4, 38.6, 38.5, 37.1, 30.2, 25.4, 25.3, 25.3, 16.1; MS (ESI) *m*/*e* 391.2 $[M + 1]^+$; HRMS calcd for $C_{21}H_{30}N_2O_3S$ $[M + 1]^+$: 391.2056; found: 391.2052.

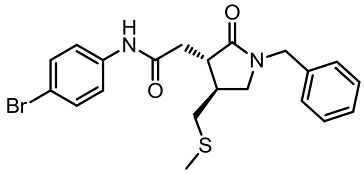

2-((3*S*,4*S*)-1-Benzyl-4-((methylthio)methyl)-2-oxopyrrolidin-3-yl)-*N*-(4-bromophenyl)acetamide (6{*4,5,2*}). Clear yellow liquid; yield 78.6 mg (44%); purity 100%; $[\alpha]_D^{25}$ −27.0 (c 0.33, $CHCl_3$); IR (neat): 3460, 3265, 3059, 2915, 1664 cm^{-1}; ^1H NMR (500 MHz, $CDCl_3$) δ 9.91 (s, 1H), 7.51–7.46 (m, 2H), 7.43–7.37 (m, 2H), 7.32–7.28 (m, 3H), 7.23–7.18 (m, 2H), 4.49 (m, 2H), 3.43 (dd, *J* = 10.1, 8.2 Hz, 1H), 3.06 (dd, *J* = 10.1, 8.6 Hz, 1H), 2.86–2.80 (m, 2H), 2.77–2.71 (m, 1H), 2.65–2.61 (m, 1H), 2.52 (dd, *J* = 13.0, 9.0 Hz, 1H), 2.43–2.34 (m, 1H), 2.09 (s, 3H); ^{13}C NMR (125 MHz, $CDCl_3$) δ 175.7, 169.2, 137.6, 135.4, 131.8, 128.9, 128.0, 128.0, 121.2, 116.3, 50.5, 47.0, 44.8, 38.5, 38.2, 36.9, 16.1; MS (ESI) *m*/*e* 447.1 $[M + 1]^+$; HRMS calcd for $C_{21}H_{23}BrN_2O_2S$ $[M + 1]^+$: 449.0713; found: 449.0721.

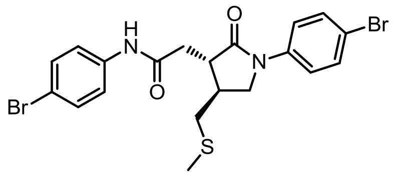

***N*-(4-Bromophenyl)-2-((3*S*,4*S*)-1-(4-bromophenyl)-4-((methylthio)methyl)-2-oxopyrrolidin-3-yl)acetamide (6{*4,5,5*})**. Pale yellow solid; mp 143–149 °C; yield 108.8 mg (53%); purity 100%; $[\alpha]_D^{26}$ −6.1 (*c* 0.44, $CHCl_3$); IR (neat): 3319, 3051, 2918, 1676 cm^{-1}; ^1H NMR (500 MHz, $CDCl_3$) δ 9.33 (s, 1H), 7.53–7.47 (m, 4H), 7.46–7.42 (m, 2H), 7.40–7.37 (m, 2H), 3.96 (dd, *J* = 9.8, 7.9 Hz, 1H), 3.61 (dd, *J* = 9.7, 8.7 Hz, 1H), 2.96–2.87 (m, 3H), 2.69–2.55 (m, 3H), 2.16 (s, 3H); ^{13}C NMR (125 MHz, $CDCl_3$) δ 175.1, 169.1, 137.7, 137.3, 132.0, 131.8, 121.6, 121.3, 118.2, 116.5, 52.2, 45.9, 37.8, 37.5, 36.8, 16.1; MS (ESI) *m*/*e* 512.0 $[M + 1]^+$; HRMS calcd for $C_{20}H_{20}Br_2N_2O_2S$ $[M + 1]^+$: 512.9671; found: 512.9663.

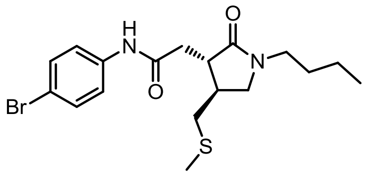

***N*-(4-Bromophenyl)-2-((3*S*,4*S*)-1-butyl-4-((methylthio)methyl)-2-oxopyrrolidin-3-yl)acetamide (6{*4,5,7*})**. Clear yellow liquid; yield 39.7 mg (24%); purity 100%; $[\alpha]_D^{26}$ −11.1 (*c* 0.42, $CHCl_3$); IR (neat): 3306, 2961, 2928, 2874, 1667 cm^{-1}; ^1H NMR (500 MHz, $CDCl_3$) δ 7.51–7.46 (m, 2H), 7.41–7.37 (m, 2H), 3.53 (dd, *J* = 10.1, 8.1 Hz, 1H), 3.32 (t, *J* = 7.4 Hz, 2H), 3.16 (dd, *J* = 10.0, 8.7 Hz, 1H), 2.88 (dd, *J* = 13.0, 4.5 Hz, 1H), 2.81–2.75 (m, 1H), 2.70–2.65 (m, 1H), 2.59–2.53 (m, 2H), 2.40–2.32 (m, *J* = 13.4, 4.4 Hz, 1H), 2.15 (s, 3H), 1.55–1.48 (m, 2H), 1.34–1.26 (m, 2H), 0.92 (dd, *J* = 9.6, 5.1 Hz, 3H); ^{13}C NMR (125 MHz, $CDCl_3$) δ 175.7, 169.3, 137.7, 131.7, 121.2, 116.2, 51.0, 44.9, 42.8, 38.8, 38.6, 36.9, 29.2, 20.0, 16.1, 13.7; MS (ESI) *m*/*e* 415.1 $[M + 1]^+$; HRMS calcd for $C_{18}H_{25}BrN_2O_2S$ $[M + 1]^+$: 415.0878; found: 415.0872.

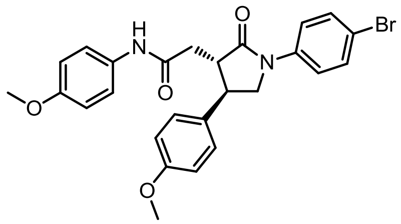

2-((3*S*,4*R*)-1-(4-Bromophenyl)-4-(4-methoxyphenyl)-2-oxopyrrolidin-3-yl)-*N*-(4-methoxyphenyl)acetamide (6{*4,6,4*}). Clear yellow liquid; yield 23.8 mg (12%); purity 97%; $[\alpha]_D^{25}$ −7.1 (c 0.11, $CHCl_3$); IR (neat): 3315, 2961, 2926, 2855, 1679 cm^{-1}; ^1H NMR (500 MHz, $CDCl_3$) δ 9.99 (s, 1H), 7.51–7.44 (m, 4H), 7.41–7.36 (m, 2H), 7.31–7.27 (m, 2H), 6.96–6.90 (m, 4H), 3.98 (dt, *J* = 11.8, 5.9 Hz, 1H), 3.91 (t, *J* = 9.9 Hz, 1H), 3.82 (s, 3H), 3.82 (s, 3H), 3.39–3.31 (m, 1H), 3.19 (ddd, *J* = 11.6, 9.0, 2.4 Hz, 1H), 2.82 (dd, *J* = 14.9, 9.0 Hz, 1H), 2.61–2.57 (m, 1H); ^{13}C NMR (125 MHz, $CDCl_3$) δ 174.8, 169.2, 159.3, 157.3, 137.6, 131.8, 131.5, 129.6, 128.8, 122.3, 121.2, 116.2, 114.6, 114.3, 55.5, 55.4, 54.9, 47.9, 44.5, 37.8; MS (ESI) *m*/*e* 511.1 $[M + 1]^+$; HRMS calcd for $C_{26}H_{25}BrN_2O_4$ $[M + 1]^+$: 511.1055; found: 511.1050.

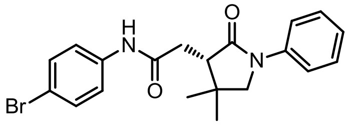

(*S*)-*N*-(4-Bromophenyl)-2-(4,4-dimethyl-2-oxo-1-phenylpyrrolidin-3-yl)acetamide (6{*4,8,1*}). Clear colorless liquid; yield 44.8 mg (28%); purity 100%; $[\alpha]_D^{27}$ −7.2 (*c* 0.22, $CHCl_3$); IR (neat): 3312, 3264, 3058, 2964, 2877, 1681 cm^{-1}; ^1H NMR (500 MHz, $CDCl_3$) δ 10.26 (s, 1H), 7.60–7.54 (m, 2H), 7.53–7.46 (m, 2H), 7.44–7.36 (m, 4H), 7.24–7.19 (m, 1H), 3.74 (d, *J* = 9.5 Hz, 1H), 3.48 (d, *J* = 9.5 Hz, 1H), 2.90–2.77 (m, 2H), 2.35 (dd, *J* = 14.0, 1.4 Hz, 1H), 1.30 (s, 3H), 1.06 (s, 3H); ^{13}C NMR (125 MHz, $CDCl_3$) δ 176.0, 170.1, 138.8, 137.7, 131.7, 129.1, 125.4, 121.2, 120.4, 116.1, 61.0, 51.2, 37.8, 34.7, 24.9, 21.8; MS (ESI) *m*/*e* 401.1 $[M + 1]^+$; HRMS calcd for $C_{20}H_{21}BrN_2O_2$ $[M + 1]^+$: 401.0864; found: 401.0855.

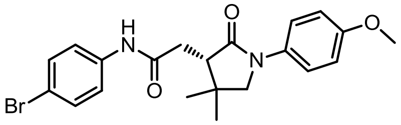

(*S*)-*N*-(4-Bromophenyl)-2-(1-(4-methoxyphenyl)-4,4-dimethyl-2-oxopyrrolidin-3-yl)acetamide (6{*4,8,4*}). White solid; mp 168–171 °C; yield 44.5 mg (28%); purity 100%; $[\alpha]_D^{26}$

−3.0 (c 0.22, $CHCl_3$); IR (neat): 3317, 2964, 2932, 2874, 1671 cm^{-1}; ^1H NMR (500 MHz, $CDCl_3$) δ 10.41 (s, 1H), 7.52–7.47 (m, 2H), 7.47–7.42 (m, 2H), 7.40–7.35 (m, 2H), 6.98–6.90 (m, 2H), 3.82 (s, 3H), 3.71 (d, *J* = 9.5 Hz, 1H), 3.41 (d, *J* = 9.5 Hz, 1H), 2.86–2.75 (m, 2H), 2.34 (d, *J* = 13.2 Hz, 1H), 1.28 (s, 3H), 1.06 (s, 3H); ^{13}C NMR (125 MHz, $CDCl_3$) δ 175.7, 170.2, 157.2, 137.8, 131.8, 131.7, 122.3, 121.2, 116.1, 114.3, 61.6, 55.5, 50.9, 38.0, 34.8, 24.9, 21.8; MS (ESI) *m*/*e* 431.1 $[M + 1]^+$; HRMS calcd for $C_{21}H_{23}BrN_2O_3$ $[M + 1]^+$: 431.0971; found: 431.0963.

Copies of ^{1}H and ^{13}C NMR spectra for representative compounds

(*R*)-2-((*S*)-2,5-Dioxo-1-phenylpyrrolidin-3-yl)propanal (3{*1,1*}) (major diastereomer)

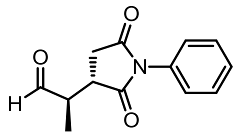

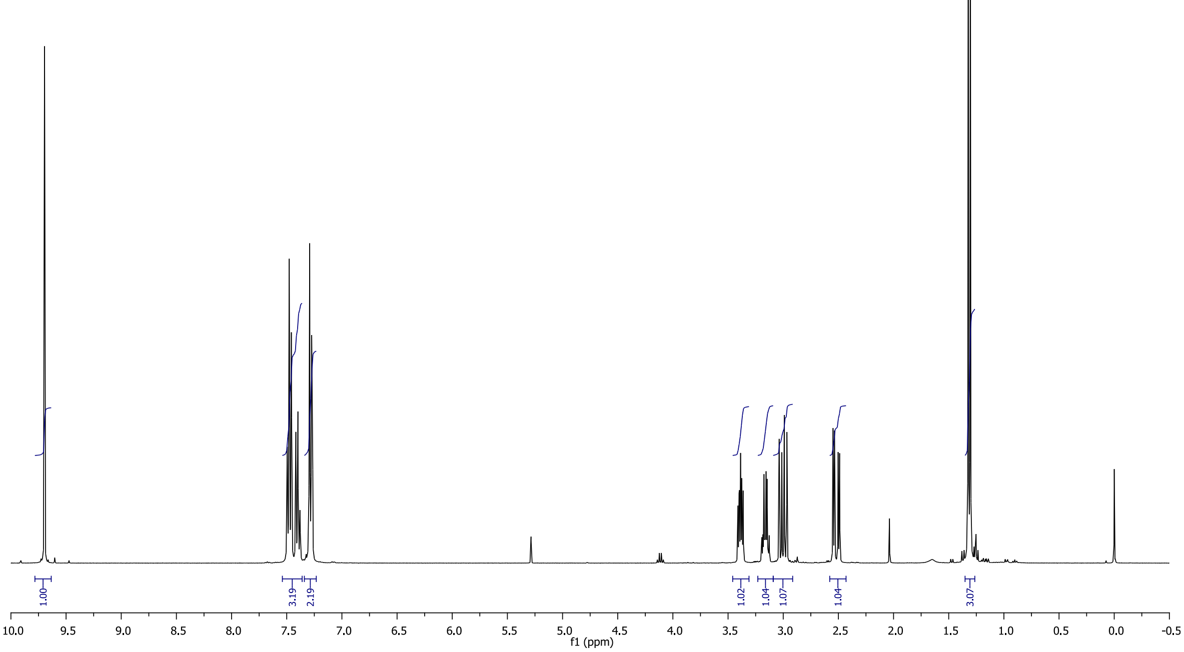

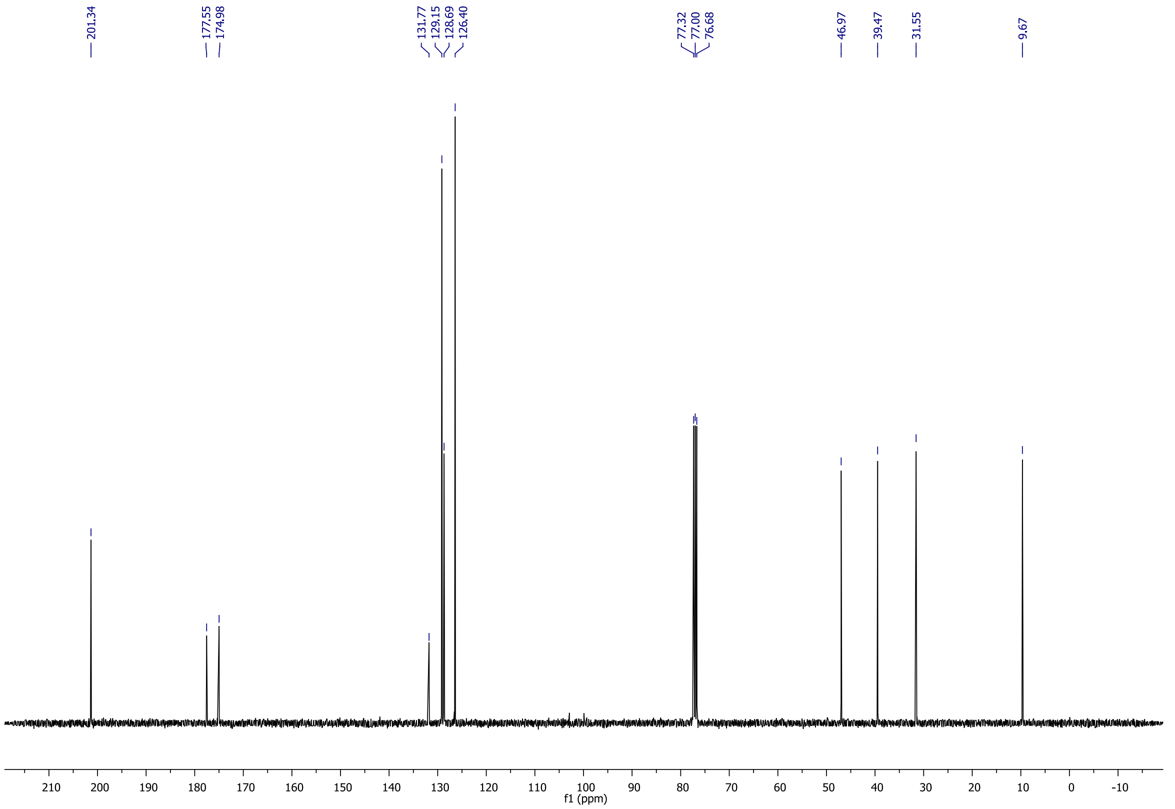

(*S*)-2-((*S*)-2,5-Dioxo-1-phenylpyrrolidin-3-yl)propanal (3{*1,1*}) (minor diastereomer)

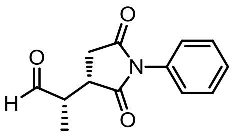

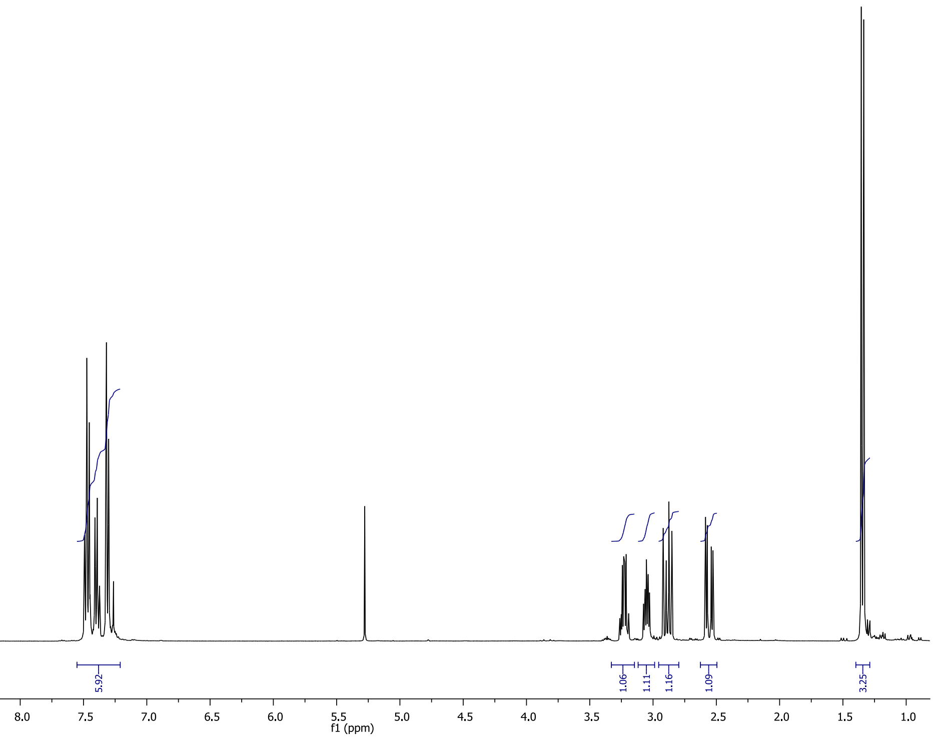

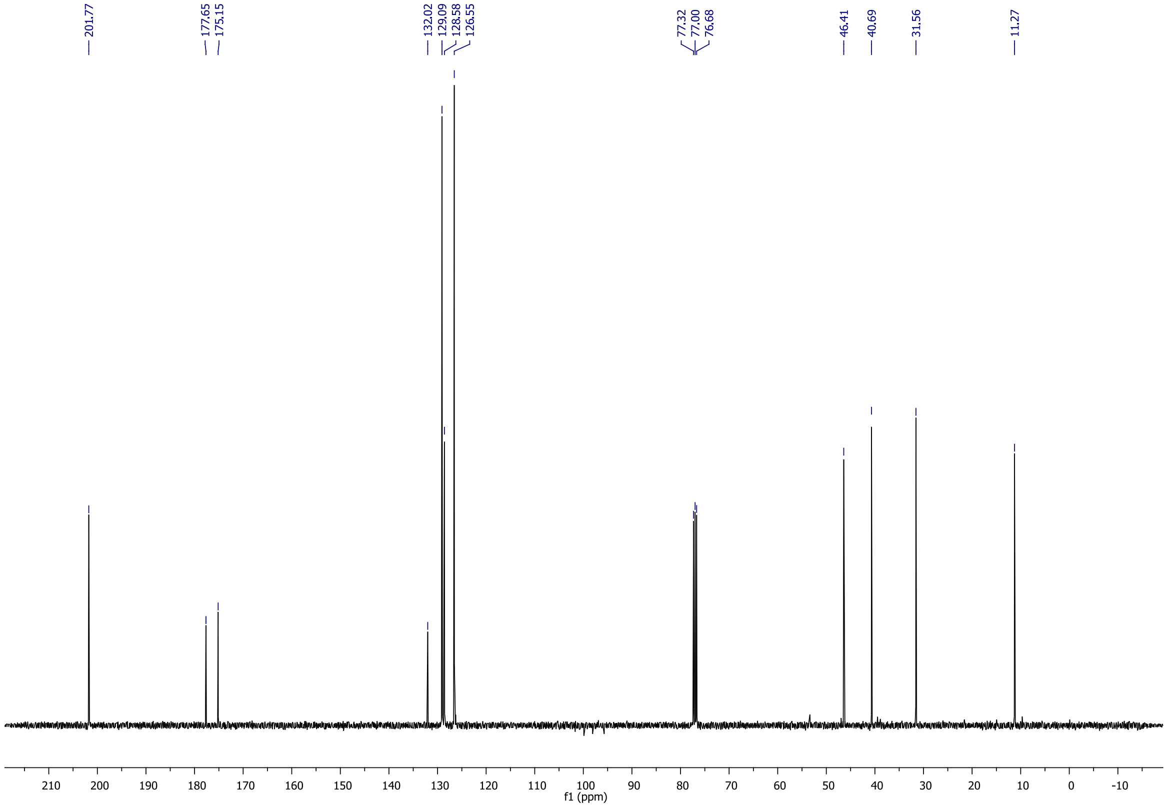

2-((3*S*,4*R*)-4-Methyl-2-oxo-1-phenylpyrrolidin-3-yl)-*N*-phenylacetamide (6{*1,1,1*}) (*syn*-diastereomer)

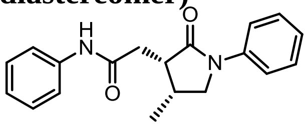

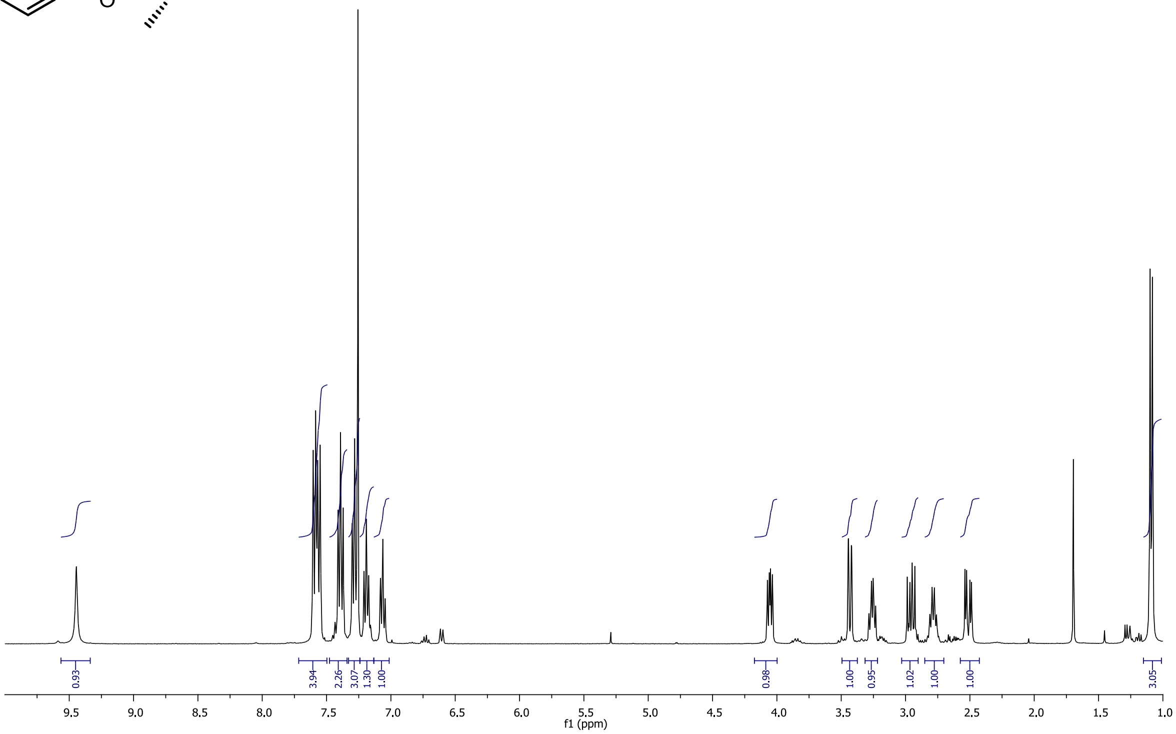

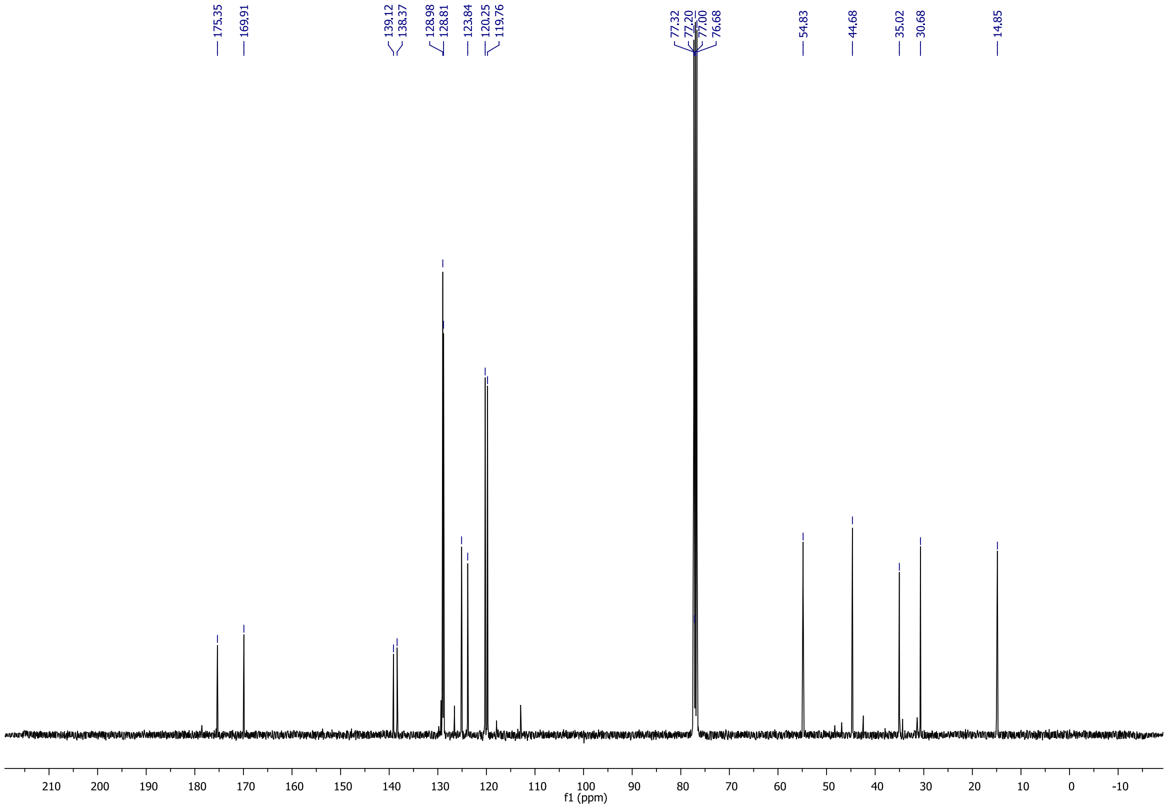

2-((3*S*,4*S*)-4-Methyl-2-oxo-1-phenylpyrrolidin-3-yl)-*N*-phenylacetamide (6{*1,1,1*}) (*trans*-diastereomer)

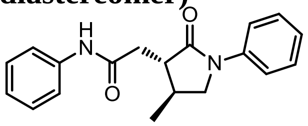

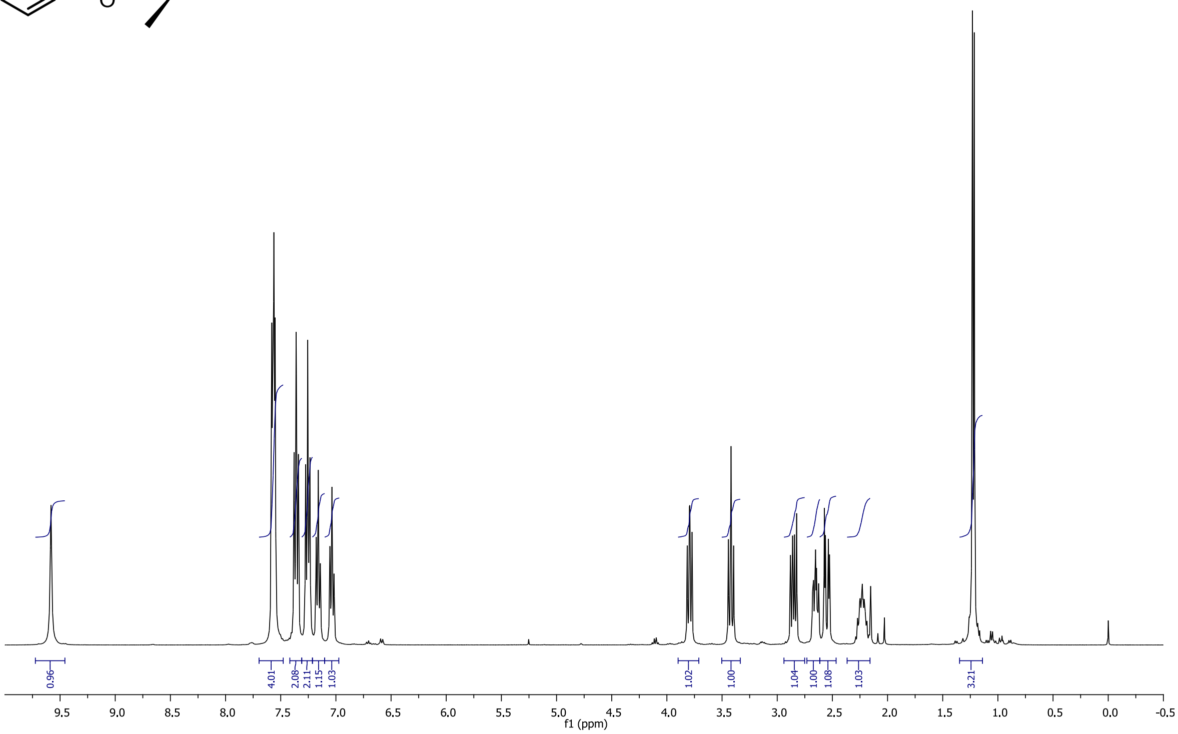

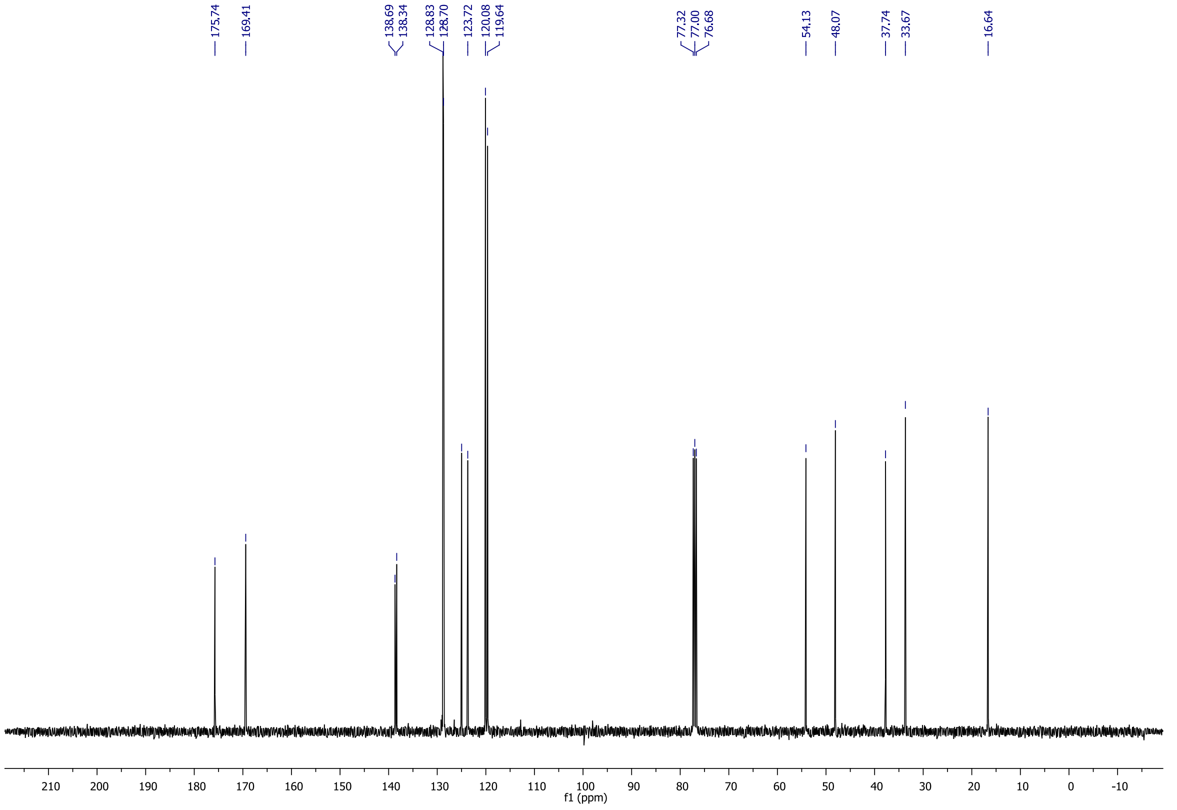

(*S*)-1-Phenyl-3-((*R*)-1-(phenylamino)propan-2-yl)pyrrolidine-2,5-dione (7{*1*,*1*,*1*})

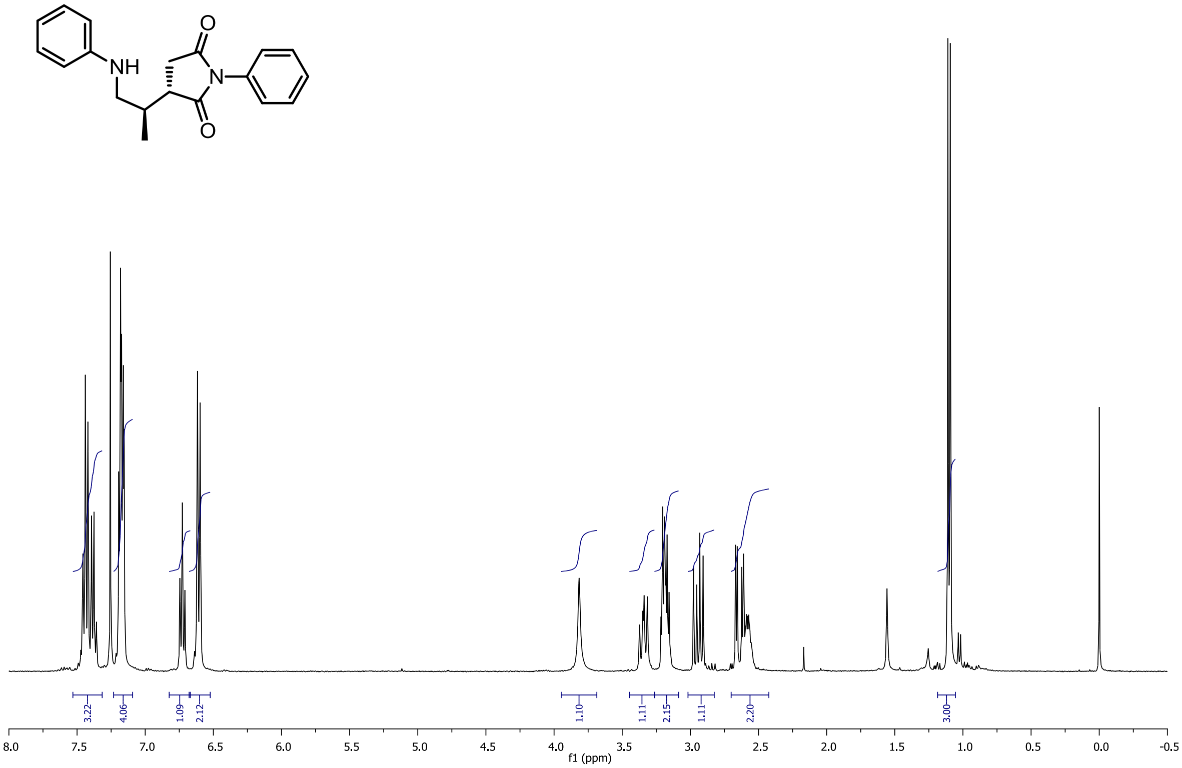

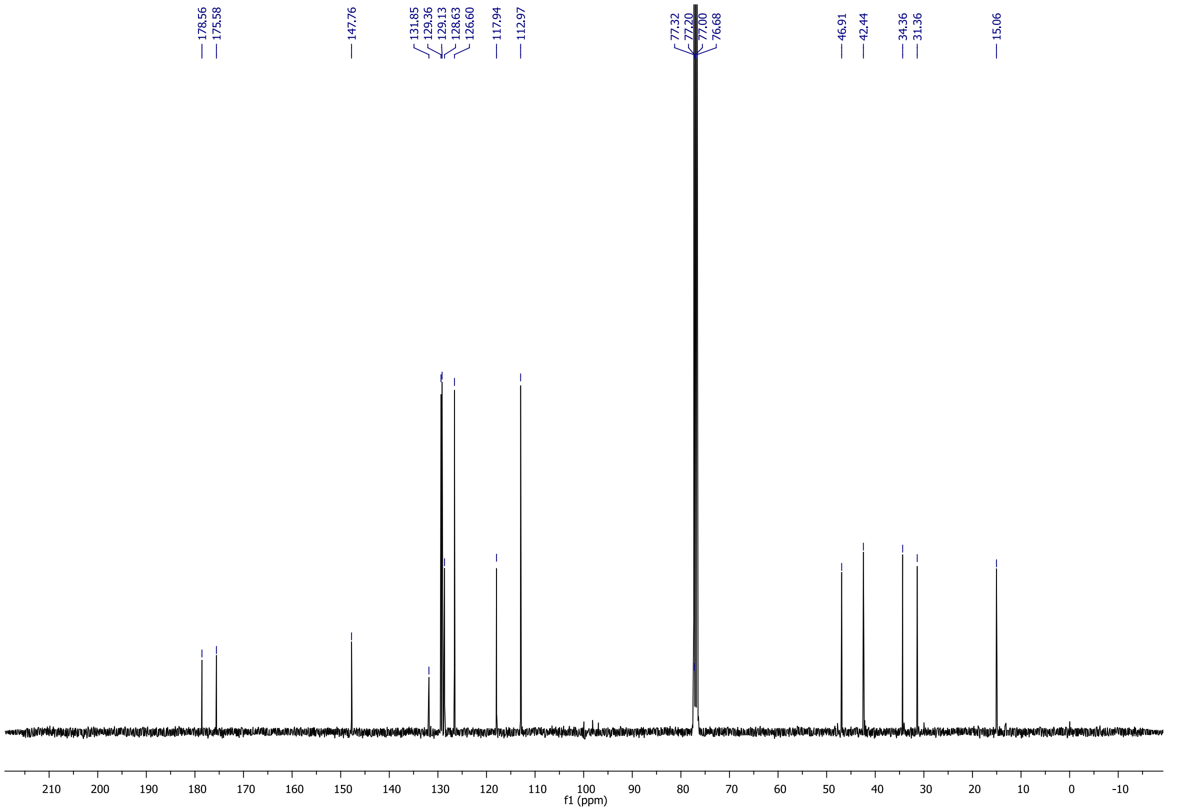

2-((3S,4S)-1-(4-Methoxyphenyl)-4-methyl-2-oxopyrrolidin-3-yl)-*N*-phenylacetamide (6{*1,1,4*})

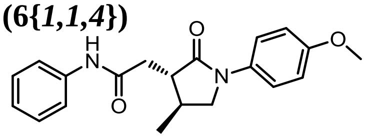

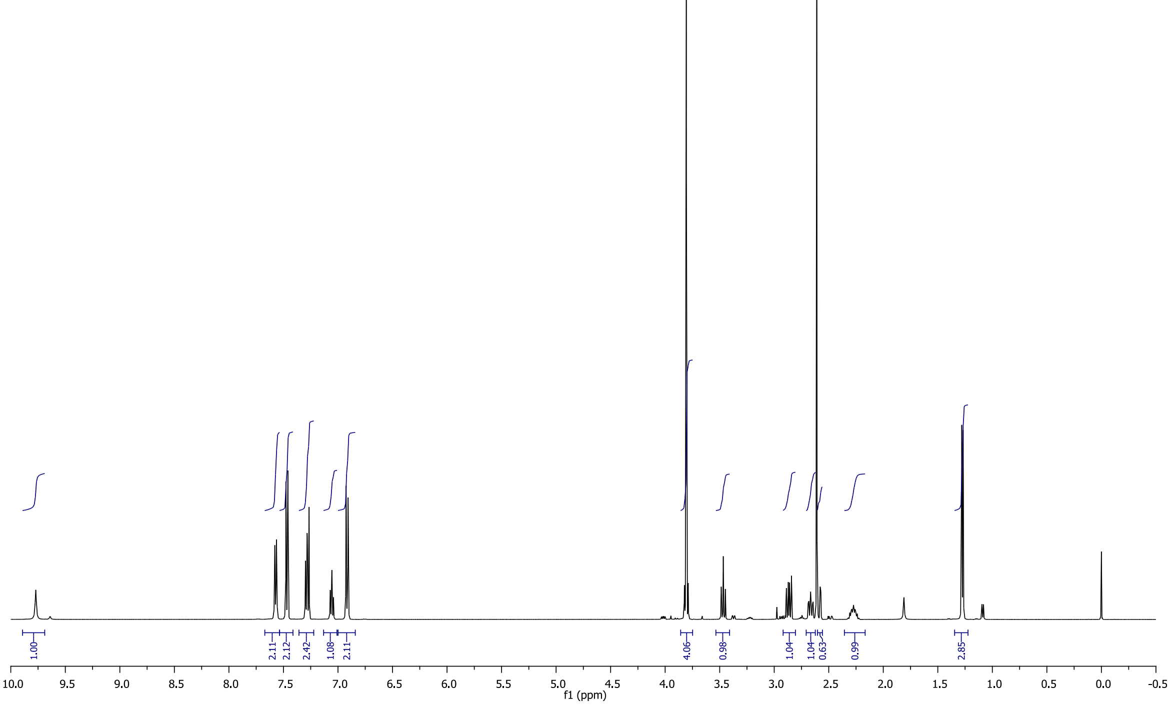

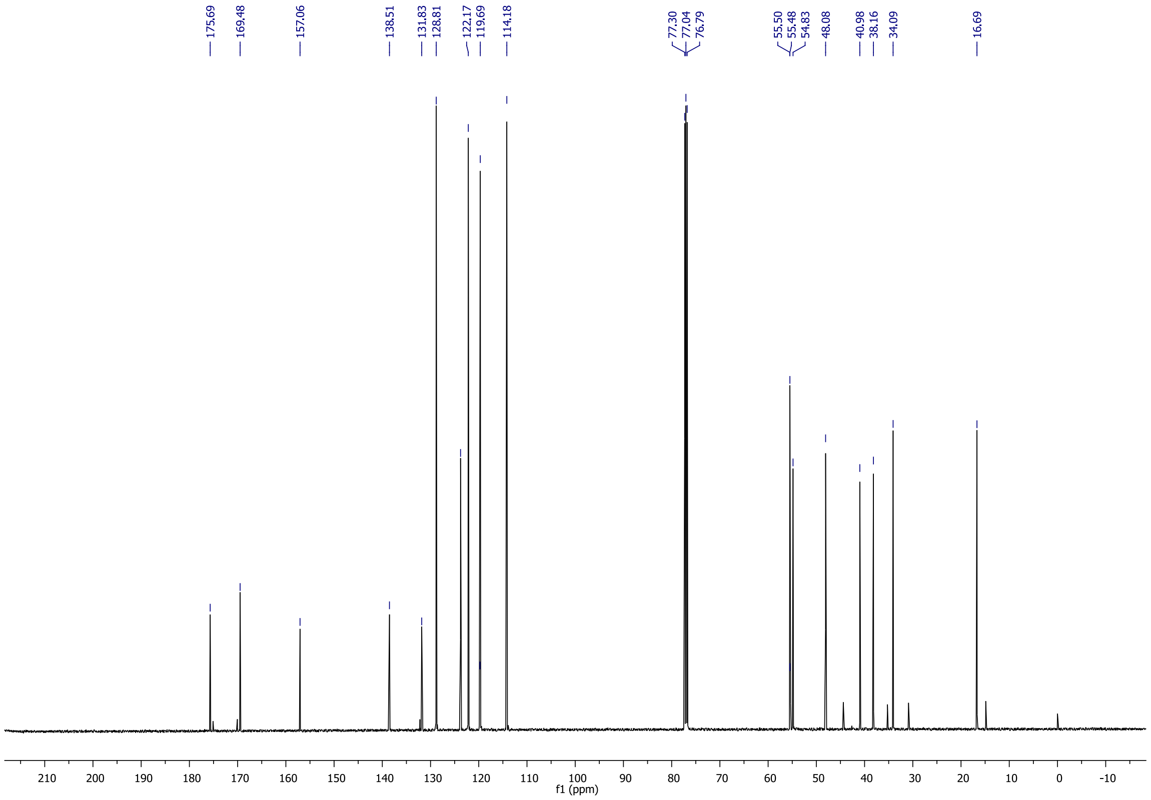

2-((3*S*,4*S*)-1-Benzyl-4-methyl-2-oxopyrrolidin-3-yl)-*N*-methylacetamide (6{*2*,*1*,*2*})

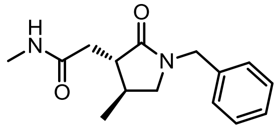

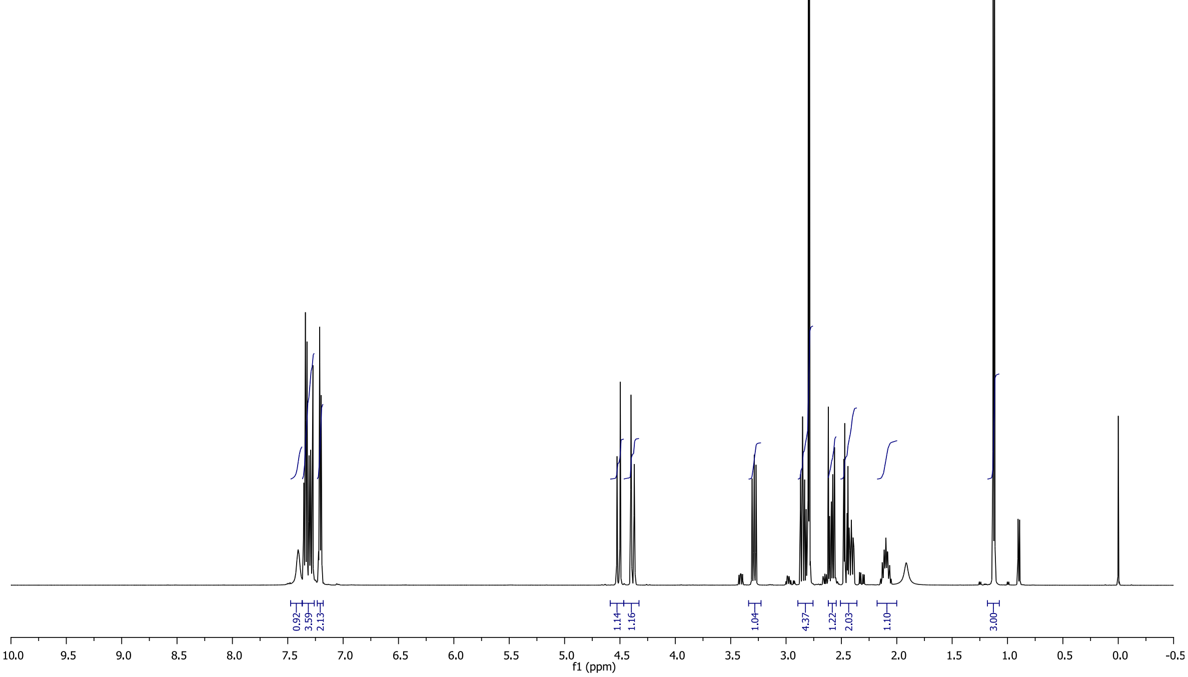

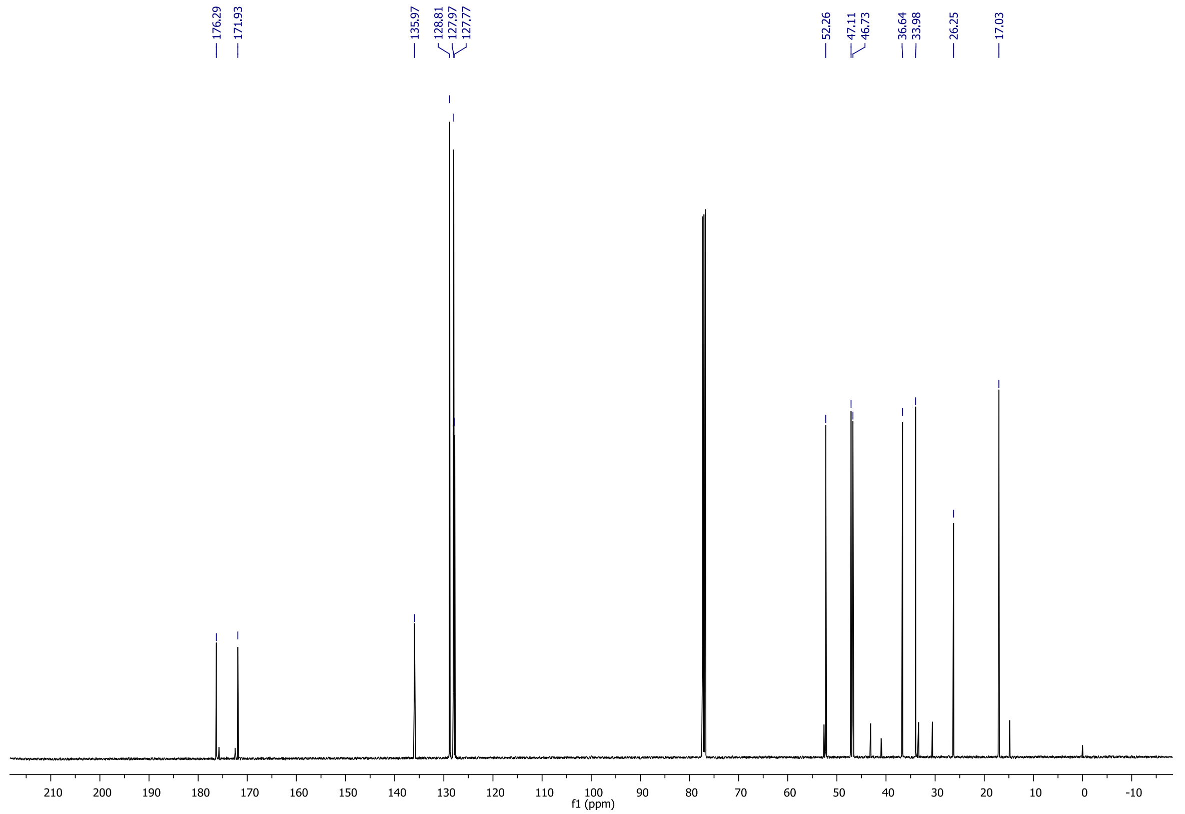

N-(4-Methoxyphenyl)-2-((3S,4S)-4-methyl-2-oxo-1-phenylpyrrolidin-3-yl)acetamide (6{3,1,1})

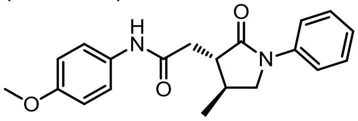

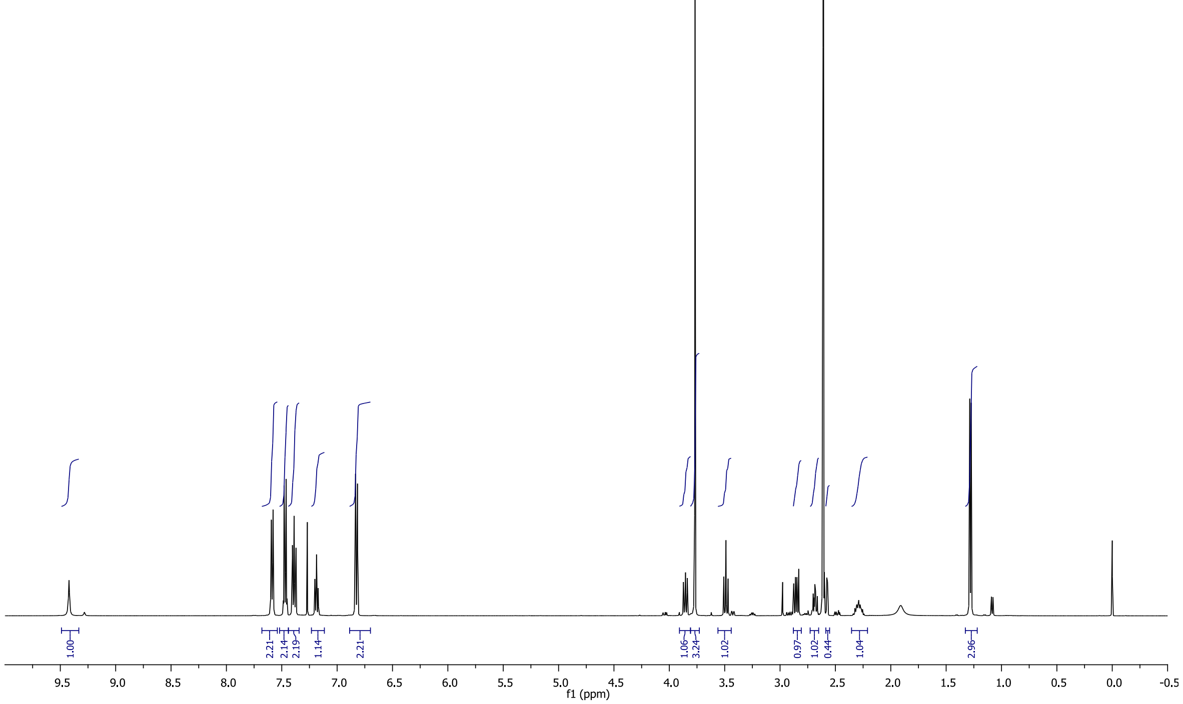

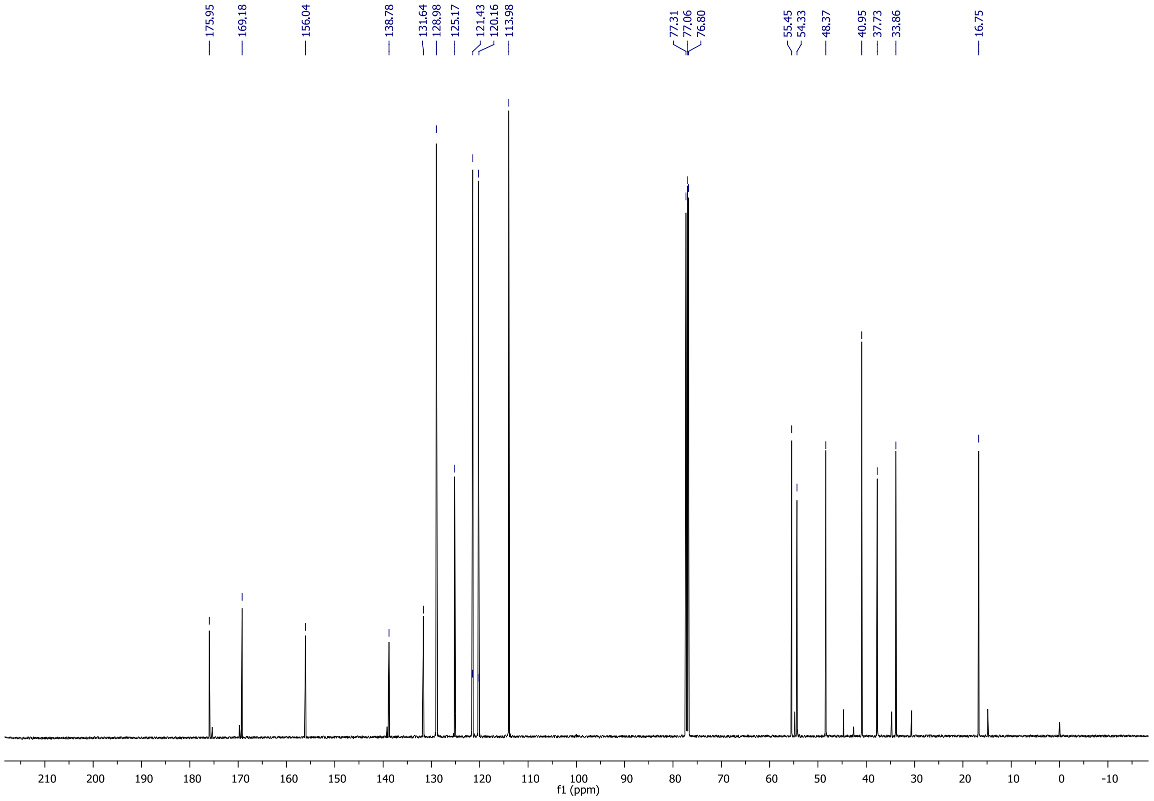

N-(4-Bromophenyl)-2-((3S,4S)-1-(4-bromophenyl)-4-methyl-2-oxopyrrolidin-3-yl)acetamide (6{4,1,5})

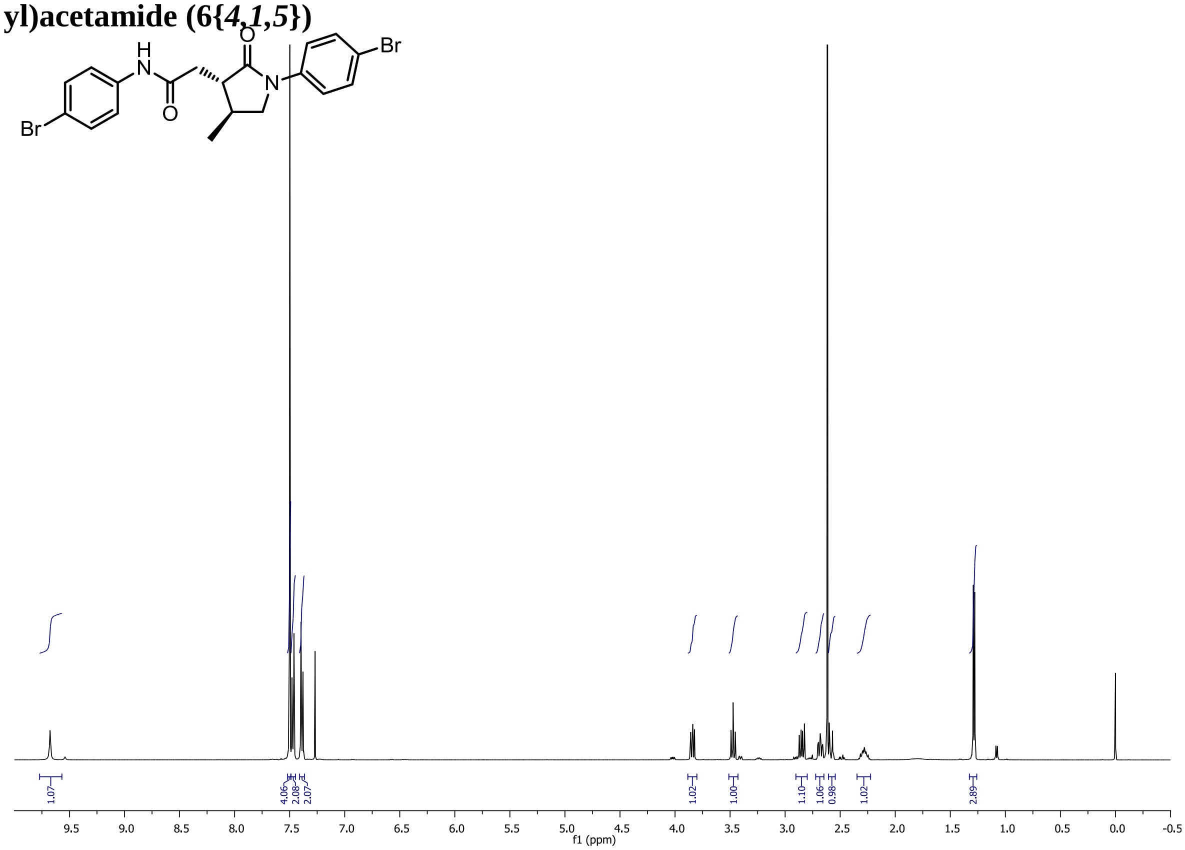

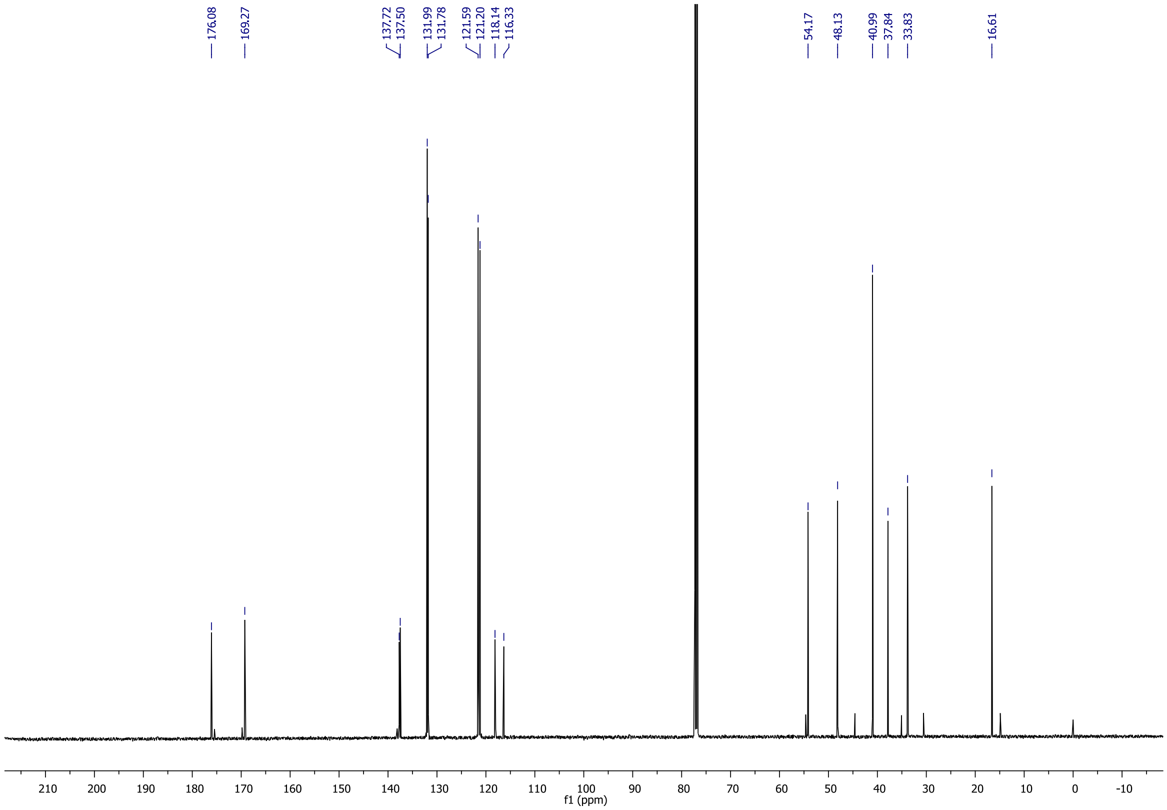

2-((3*S*,4*S*)-4-Benzyl-2-oxo-1-phenylpyrrolidin-3-yl)-*N*-phenylacetamide (6{*1,3,2*})

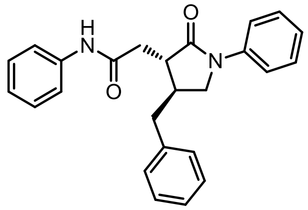

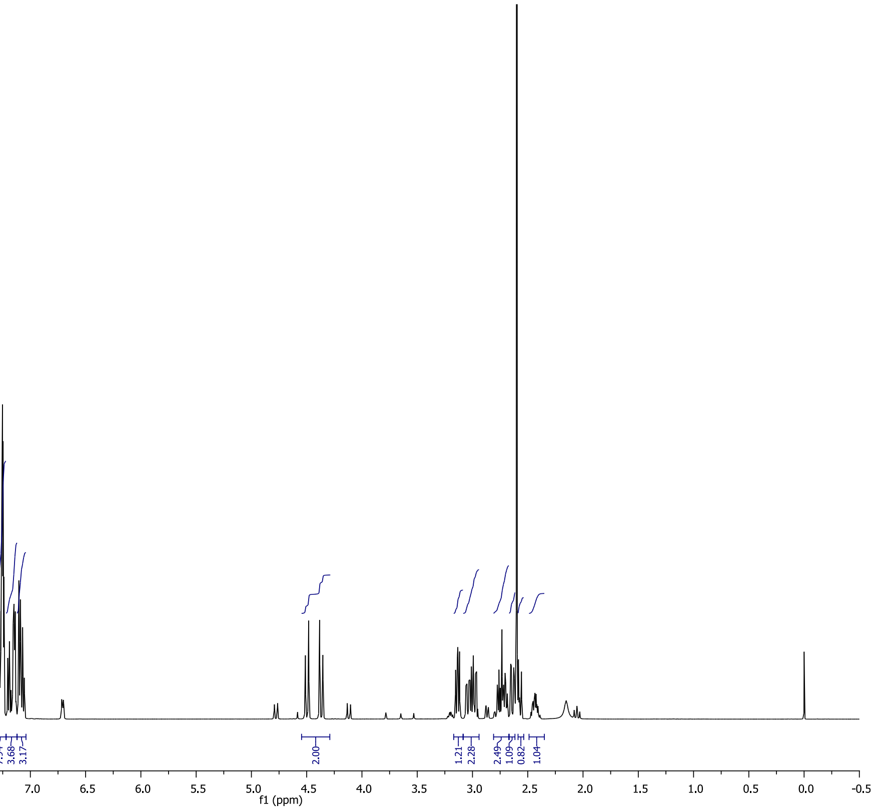

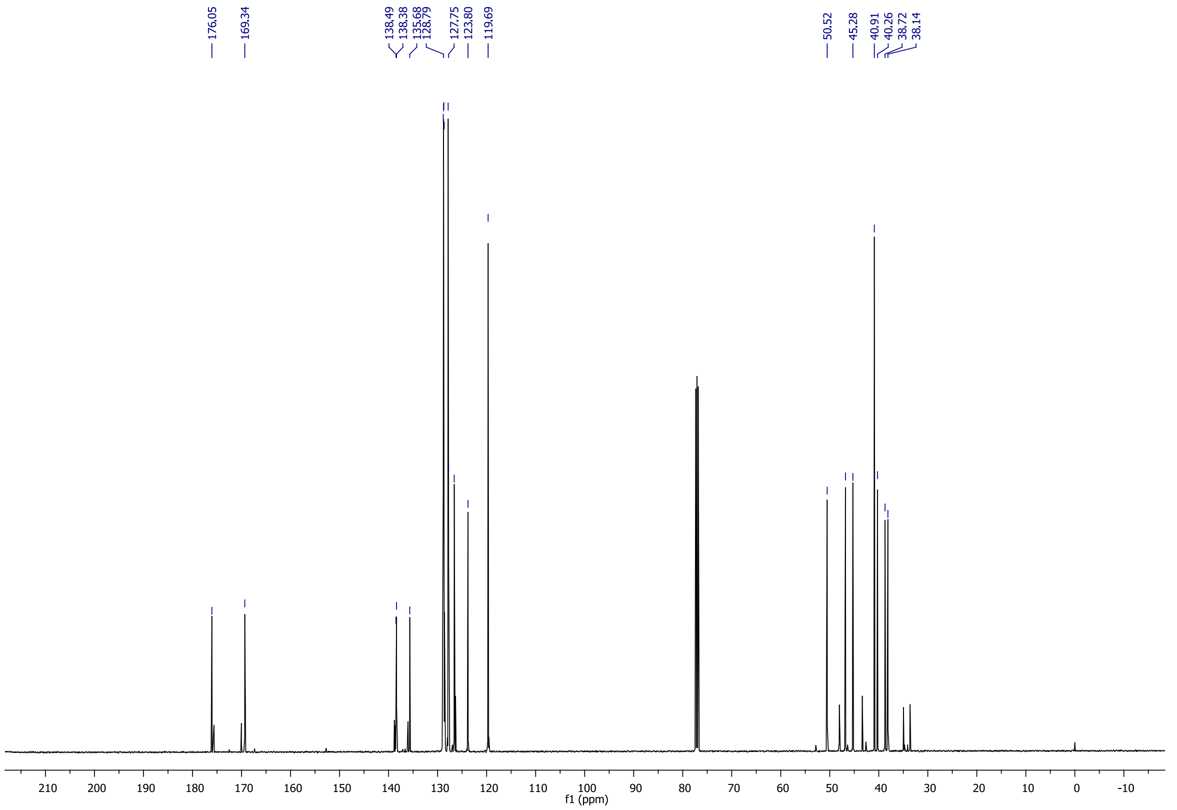

2-((3*S*,4*S*)-4-Benzyl-2-oxo-1-(pyridin-2-ylmethyl)pyrrolidin-3-yl)-*N*-phenylacetamide (6{*1,3,8*})

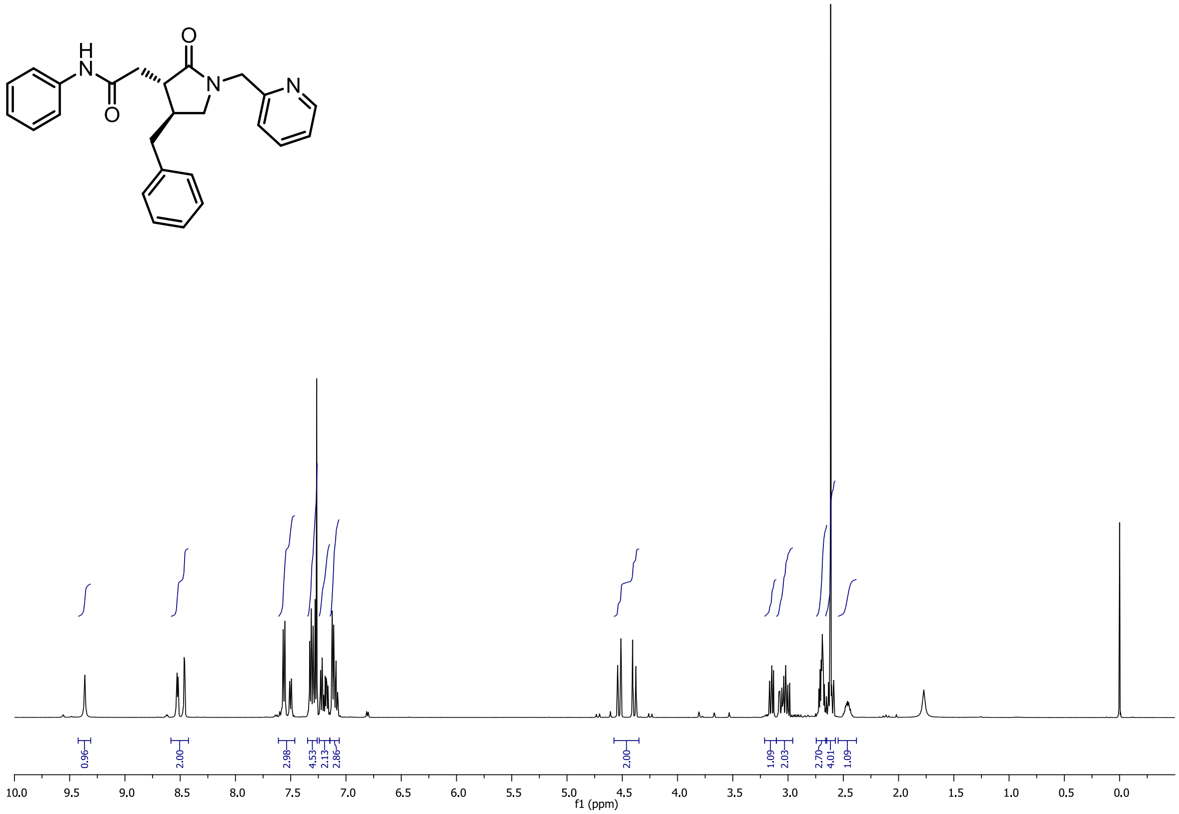

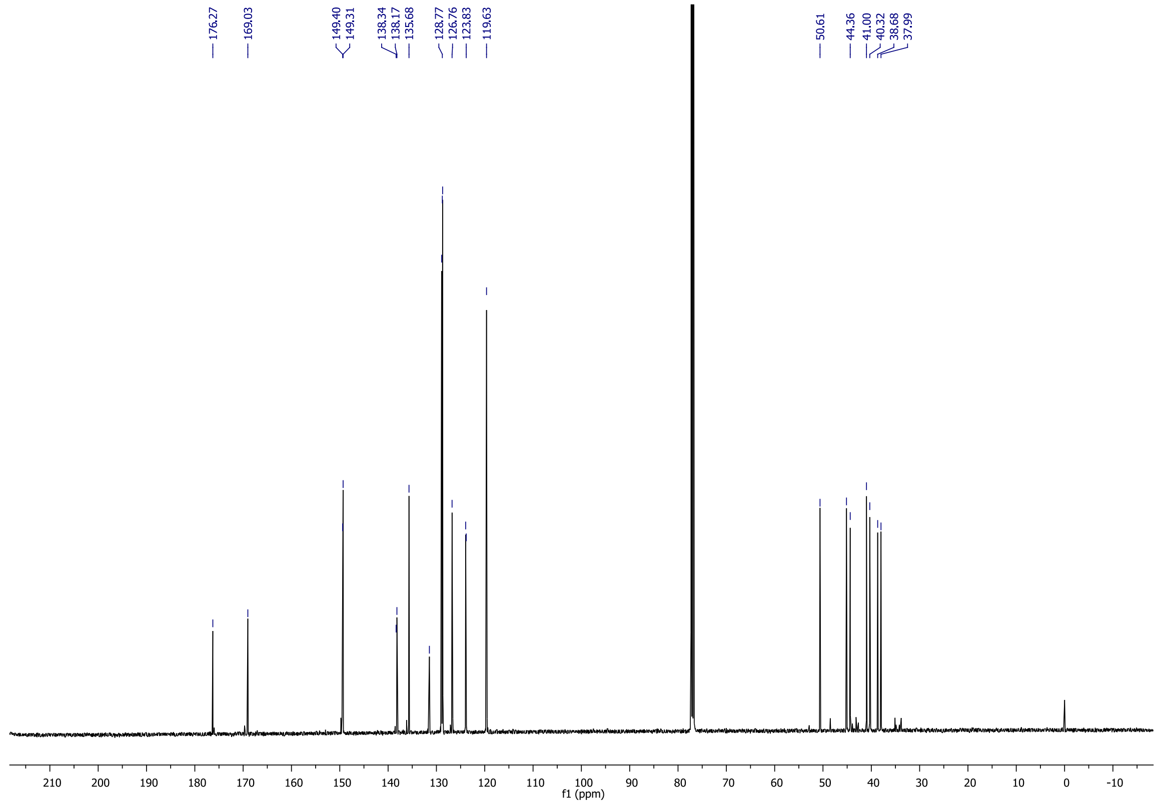

2-((3*S*,4*S*)-1-(4-Methoxyphenyl)-2-oxo-4-propylpyrrolidin-3-yl)-*N*-phenylacetamide (6{*1,4,4*})

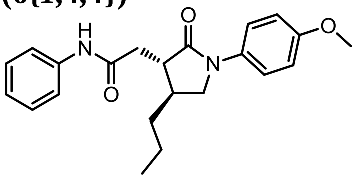

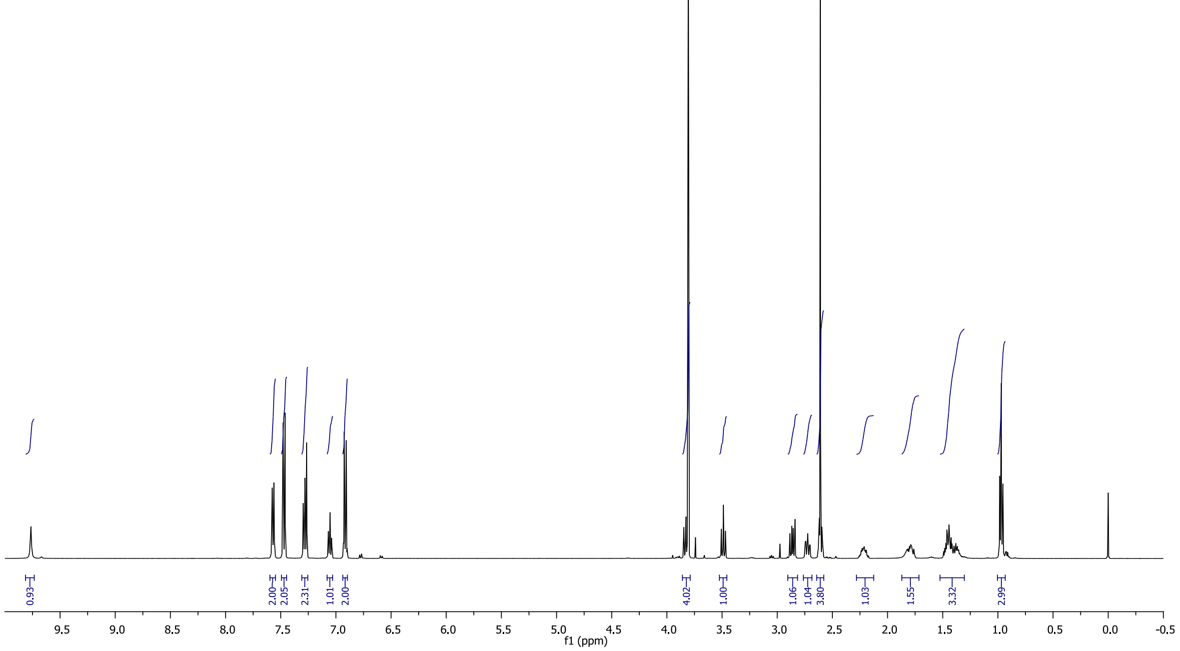

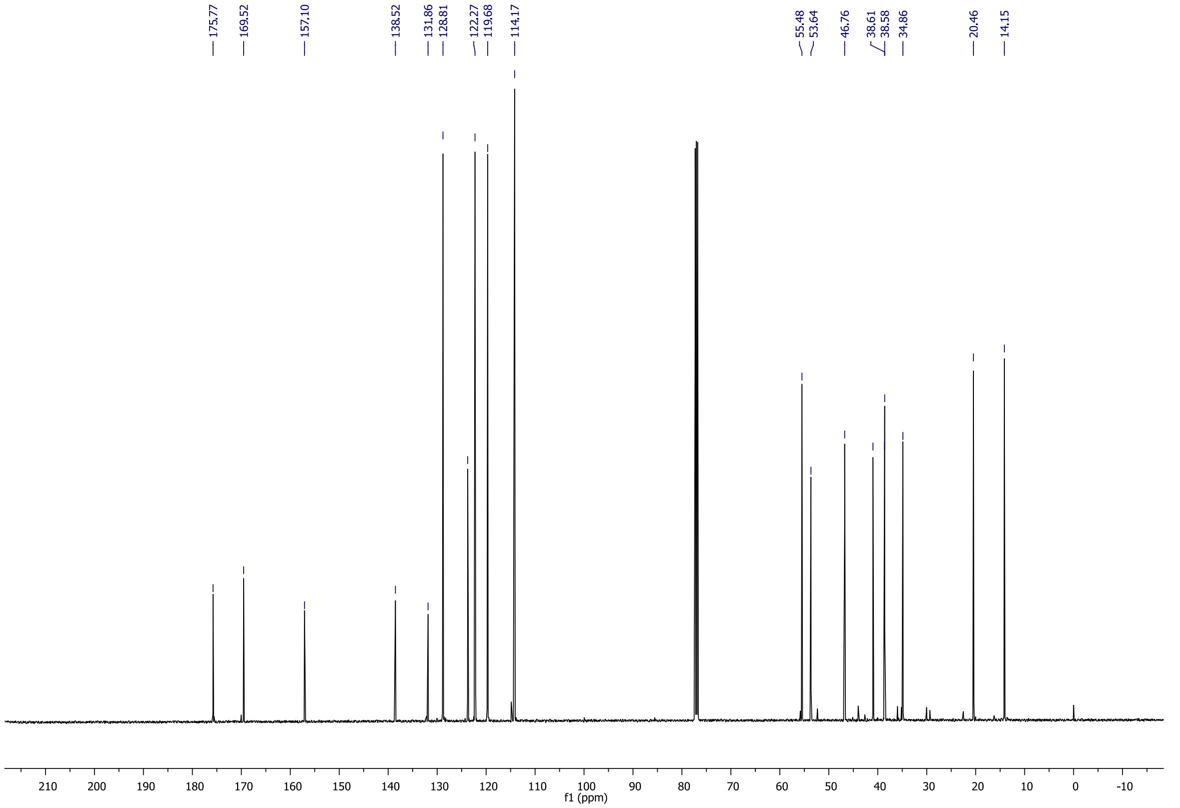

2-((3*S*,4*S*)-1-(4-Methoxyphenyl)-2-oxo-4-propylpyrrolidin-3-yl)-*N*-methylacetamide (6{*2,4,4*})

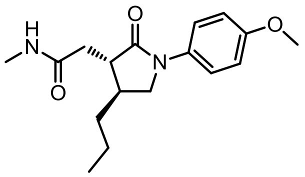

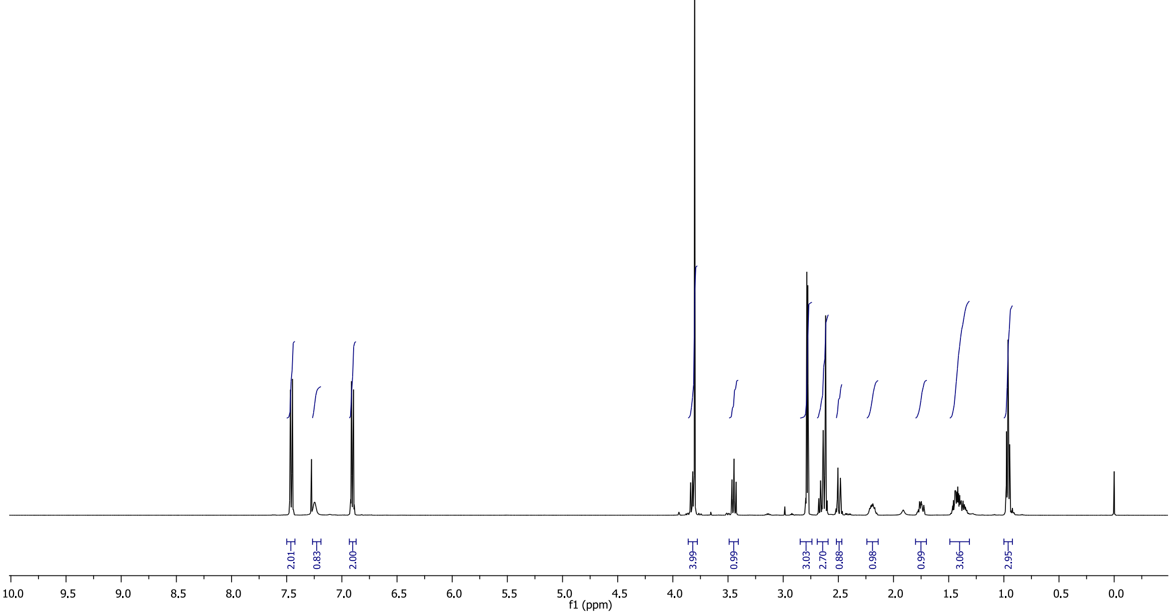

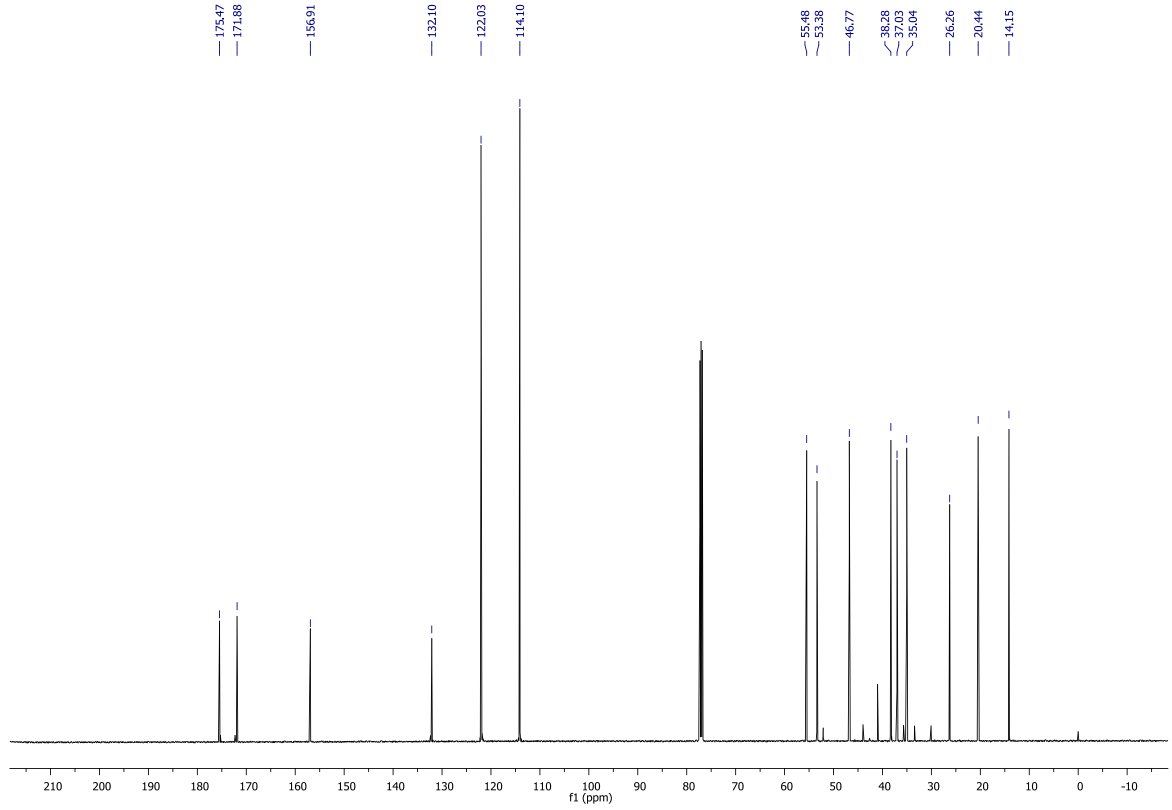

2-((3*S*,4*S*)-1-Cyclohexyl-4-methyl-2-oxopyrrolidin-3-yl)-*N*-phenylacetamide (6{*3,3,6*})

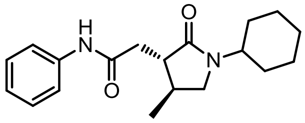

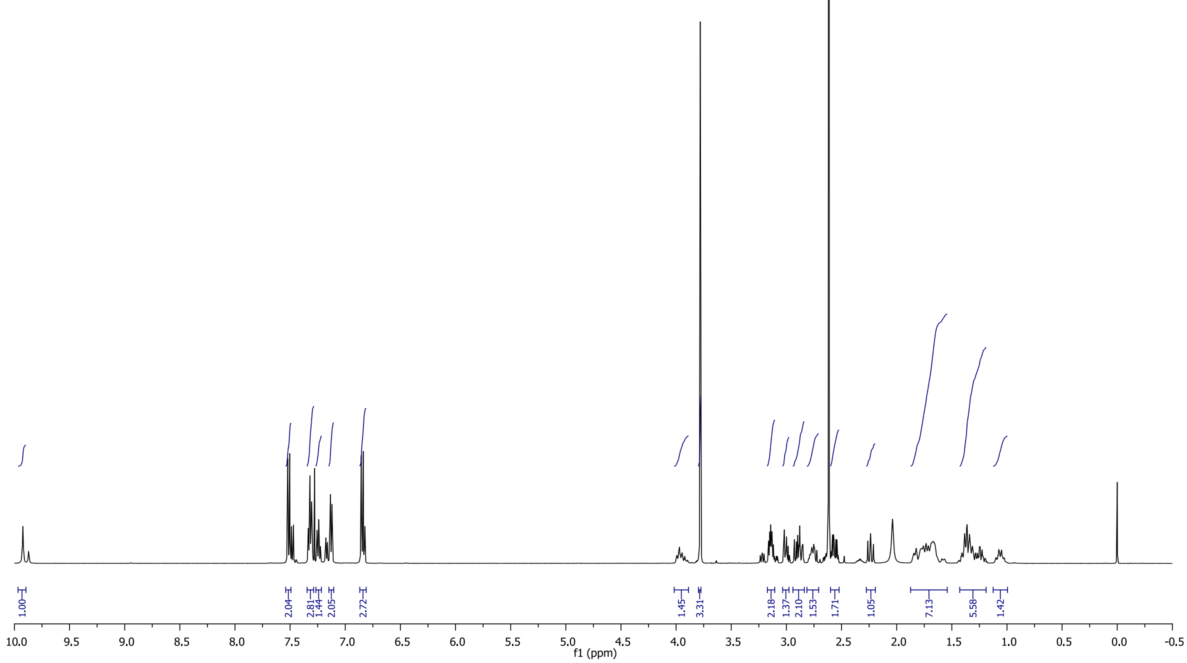

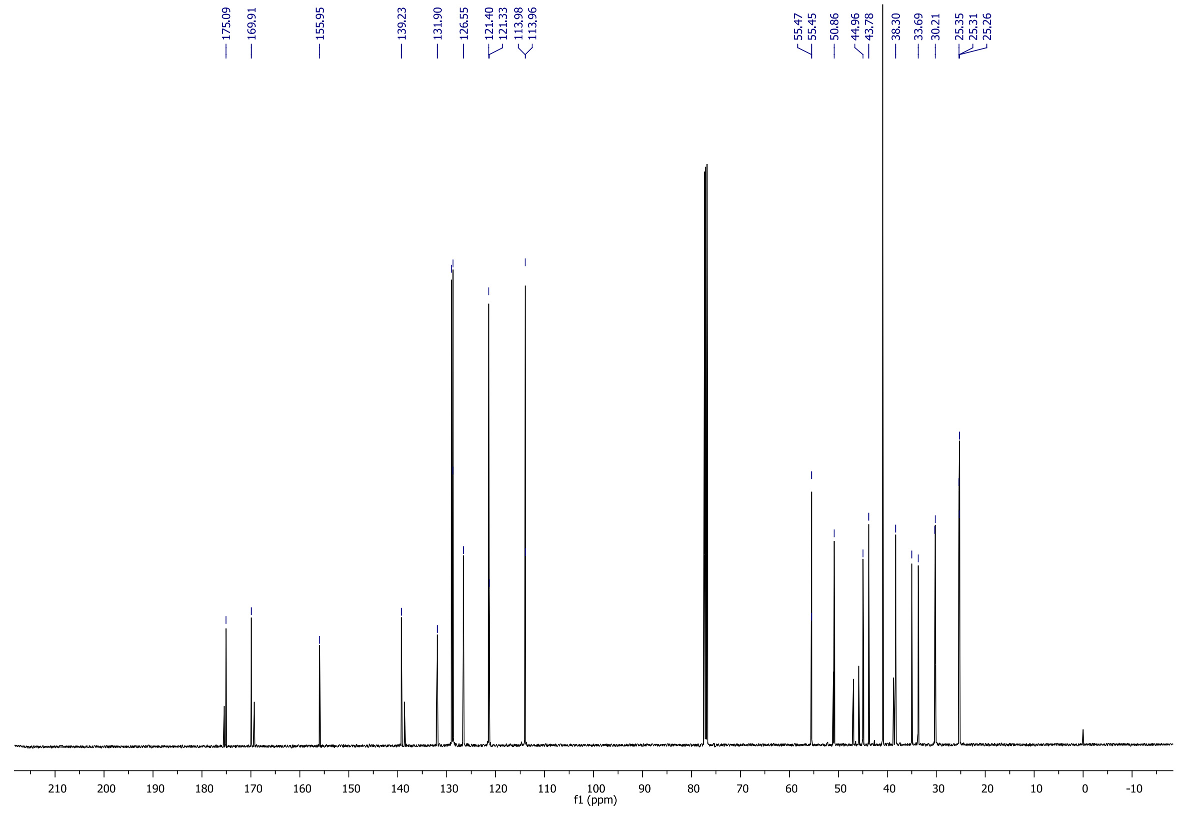

_N_-(4-Bromophenyl)-2-((3_S_,4_S_)-2-oxo-4-propyl-1-(pyridin-2-ylmethyl)pyrrolidin-3-yl)acetamide (6{_4_,_4_,_8_})

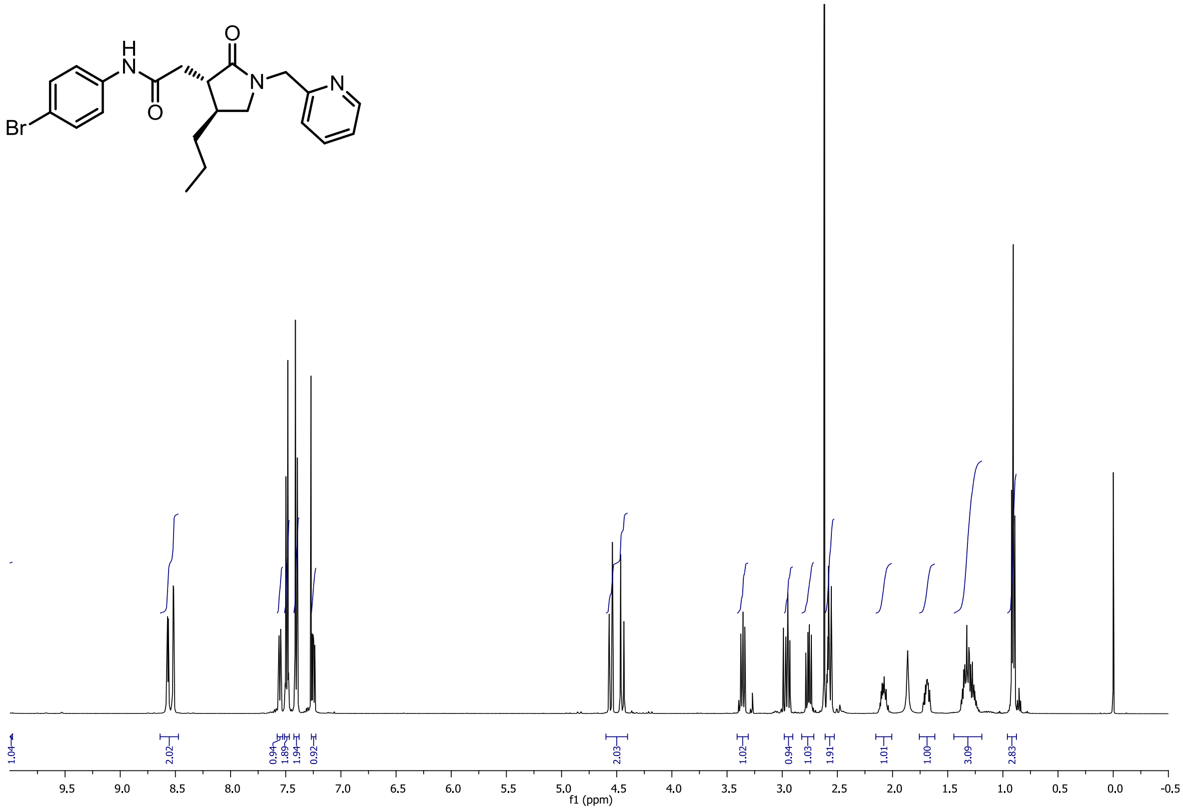

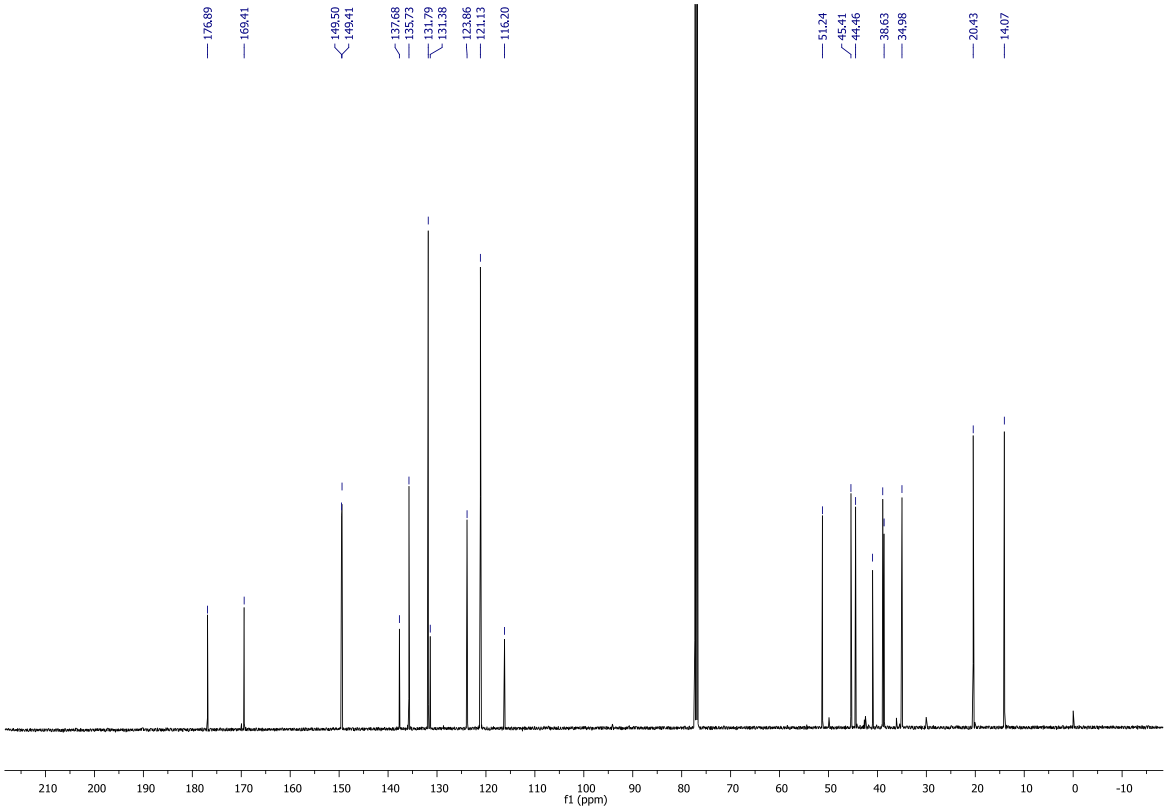

_N_-Methyl-2-((_3S,4S_)-4-((methylthio)methyl)-2-oxo-1-phenethylpyrrolidin-3-yl)acetamide (6{_2,5,3_})

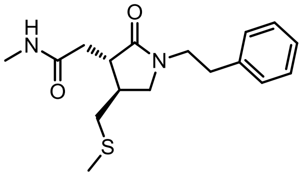

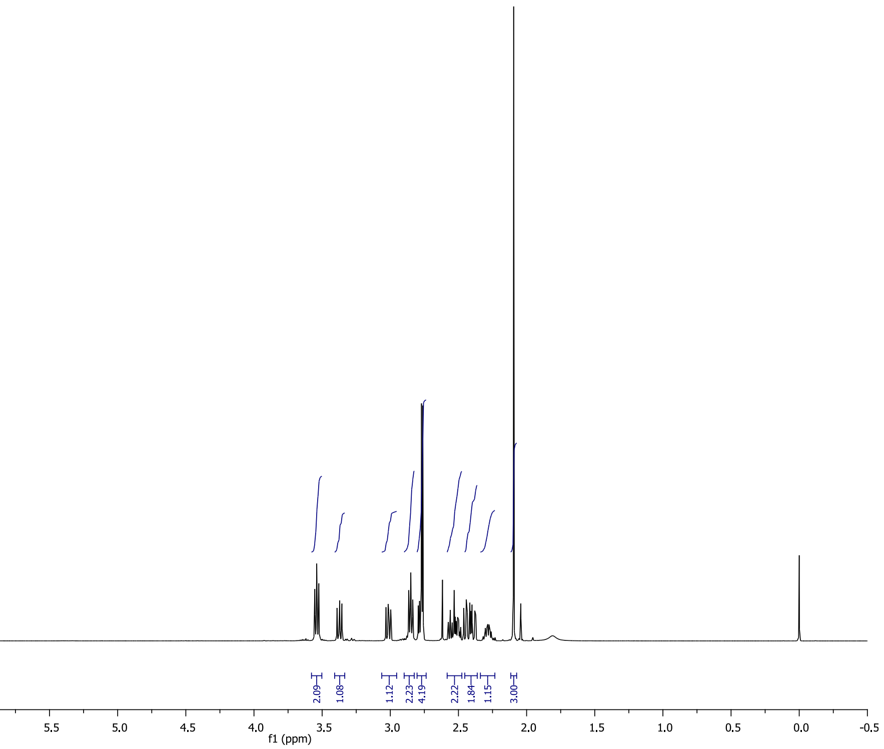

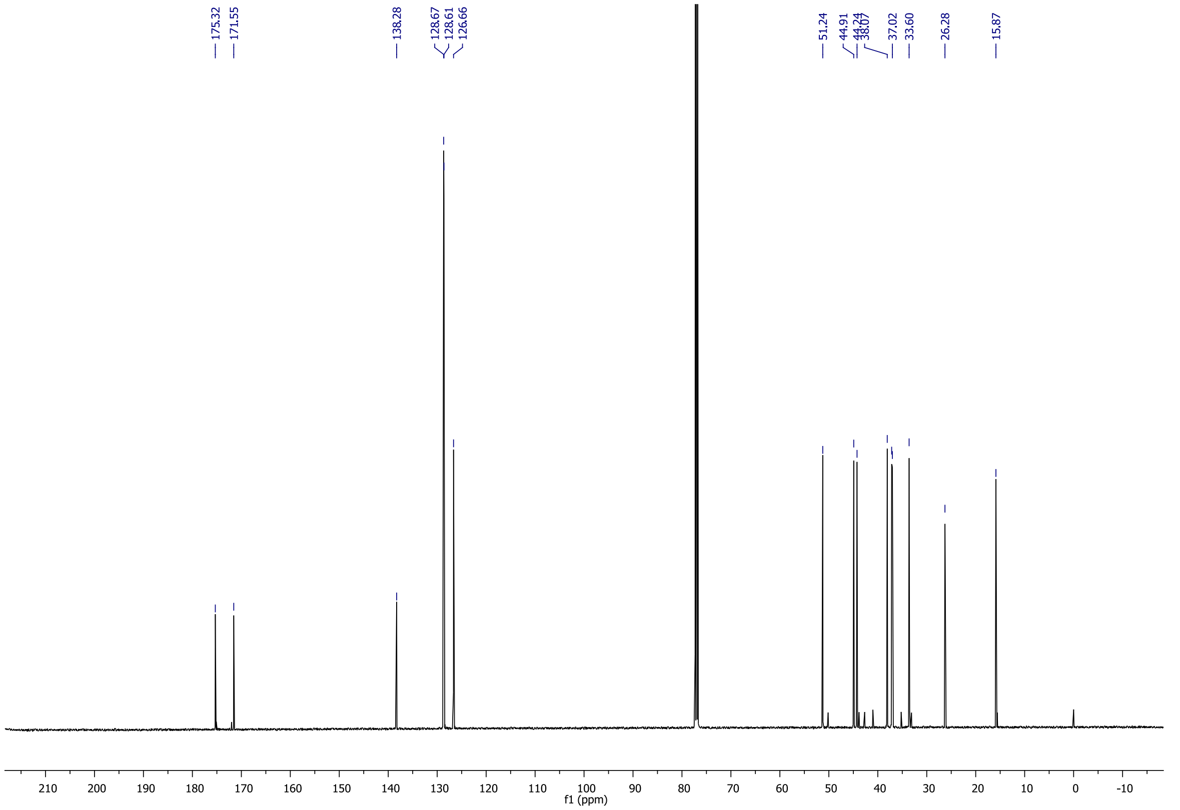

2-((3*S*,4*S*)-1-Benzyl-4-((methylthio)methyl)-2-oxopyrrolidin-3-yl)-*N*-(4-methoxyphenyl)acetamide (6{*3,5,2*})

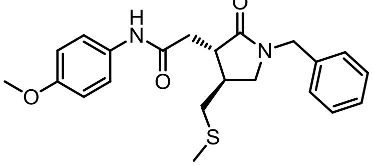

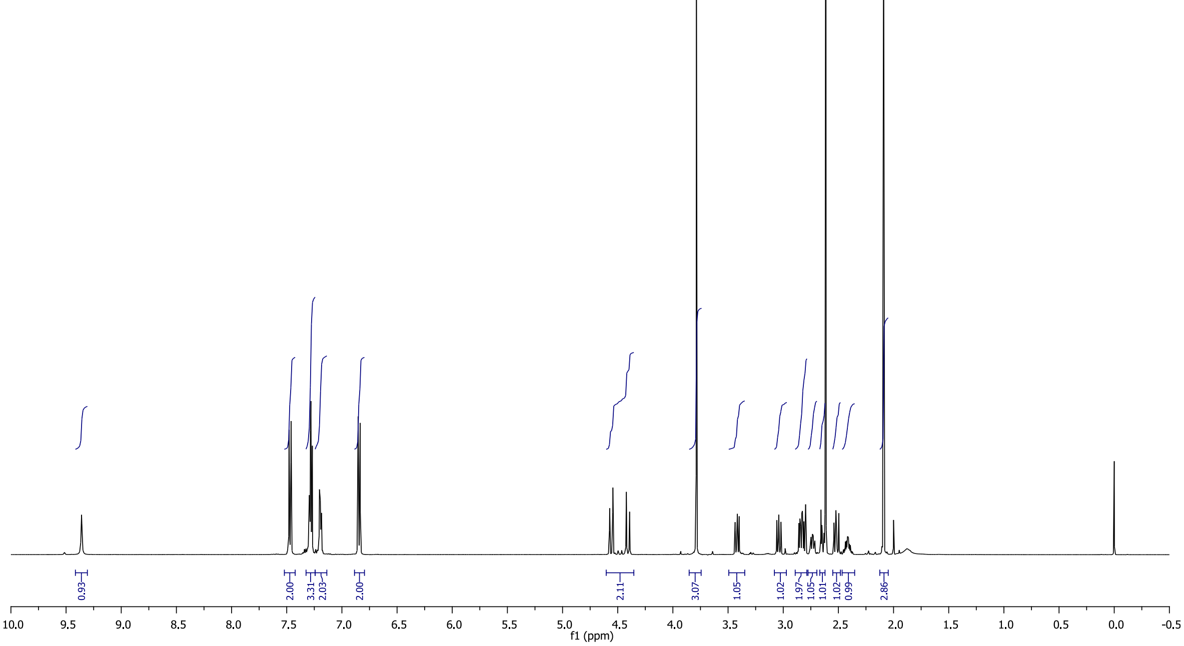

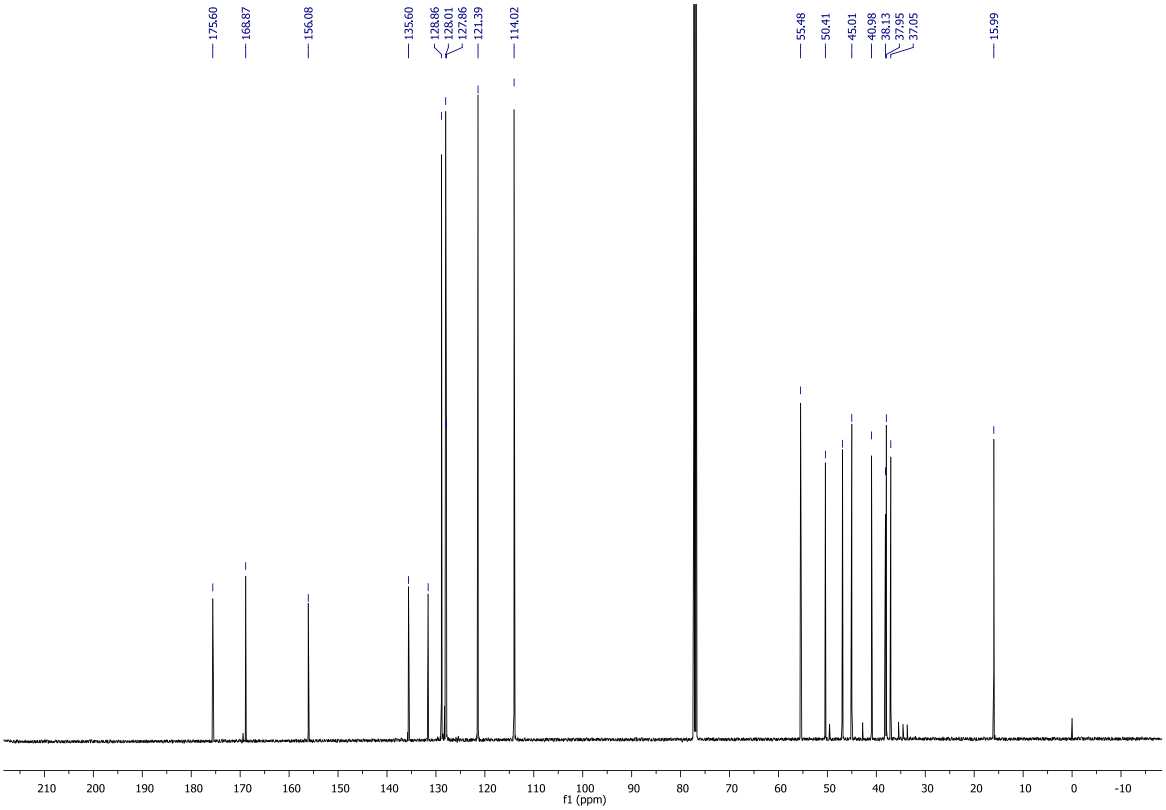

2-((3*S*,4*S*)-1-Cyclohexyl-4-((methylthio)methyl)-2-oxopyrrolidin-3-yl)-*N*-(4-methoxyphenyl)acetamide (6{*3,5,6*})

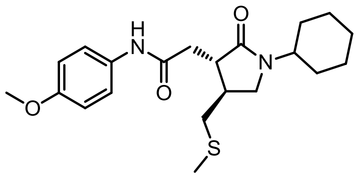

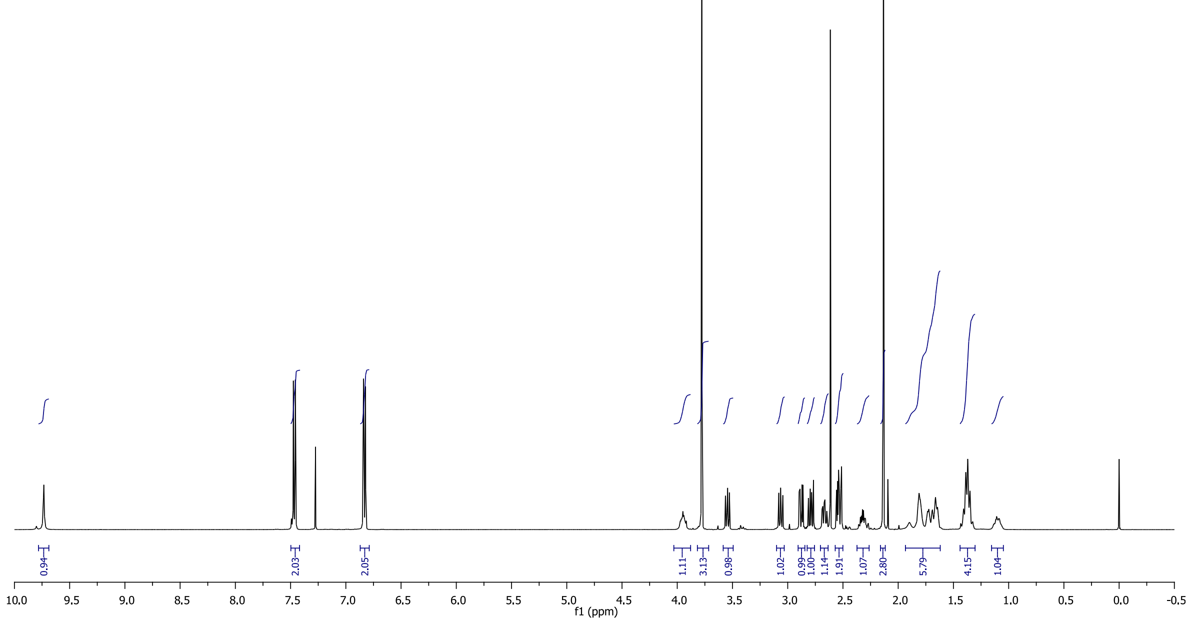

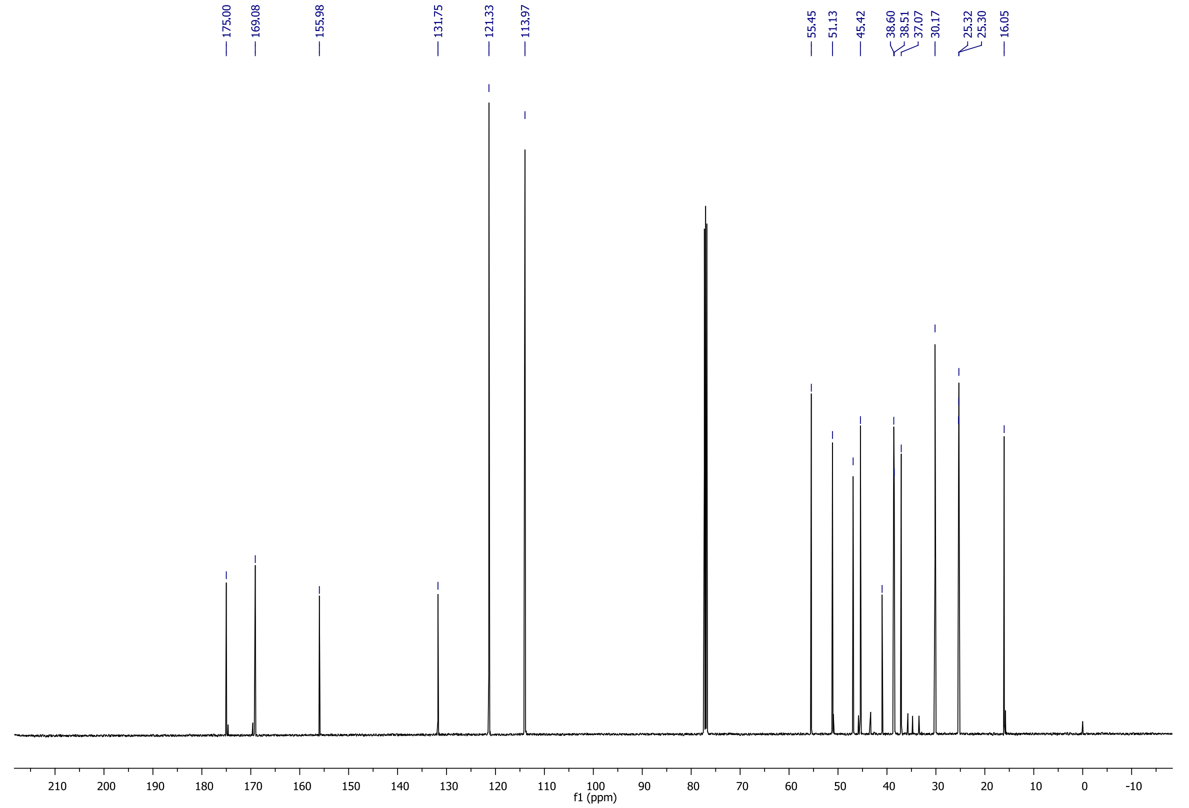

2-((3*S*,4*S*)-1-Benzyl-4-((methylthio)methyl)-2-oxopyrrolidin-3-yl)-*N*-(4-bromophenyl)acetamide (6{*4,5,2*})

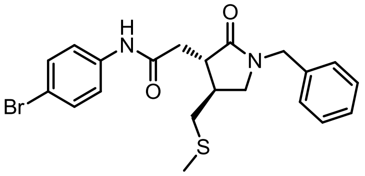

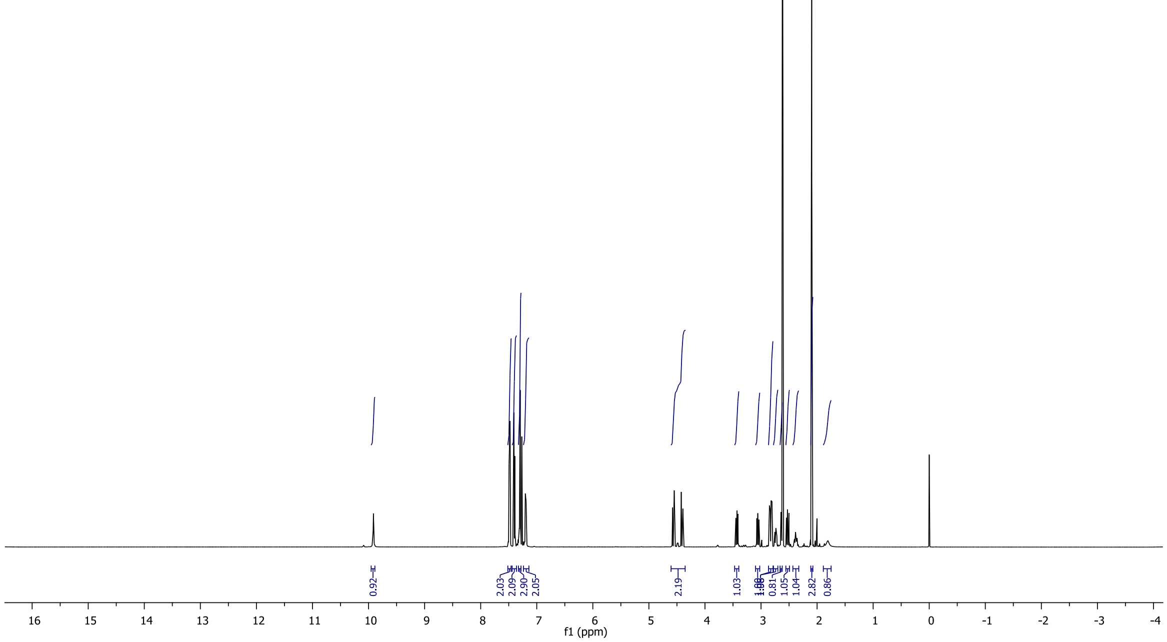

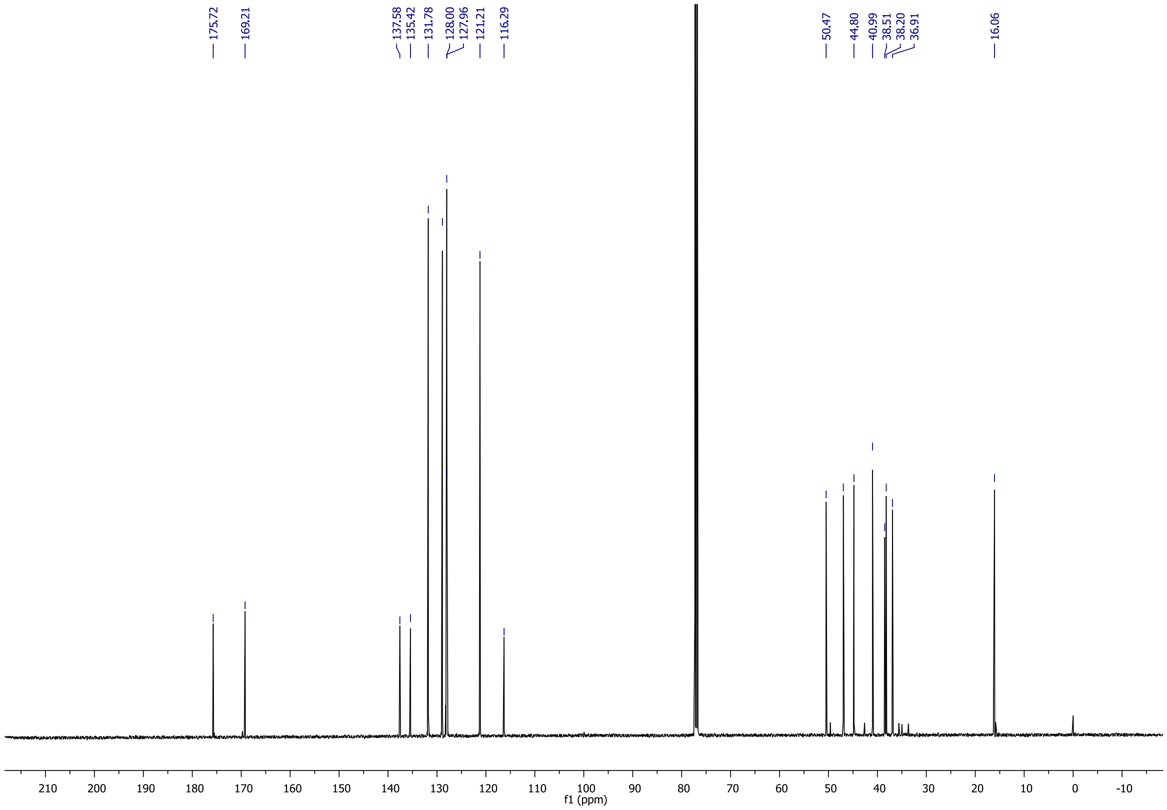

_N_-(4-Bromophenyl)-2-((3_S_,4_S_)-1-(4-bromophenyl)-4-((methylthio)methyl)-2-oxopyrrolidin-3-yl)acetamide (6{_4,5,5_})

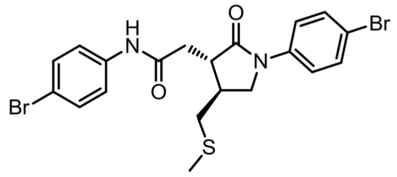

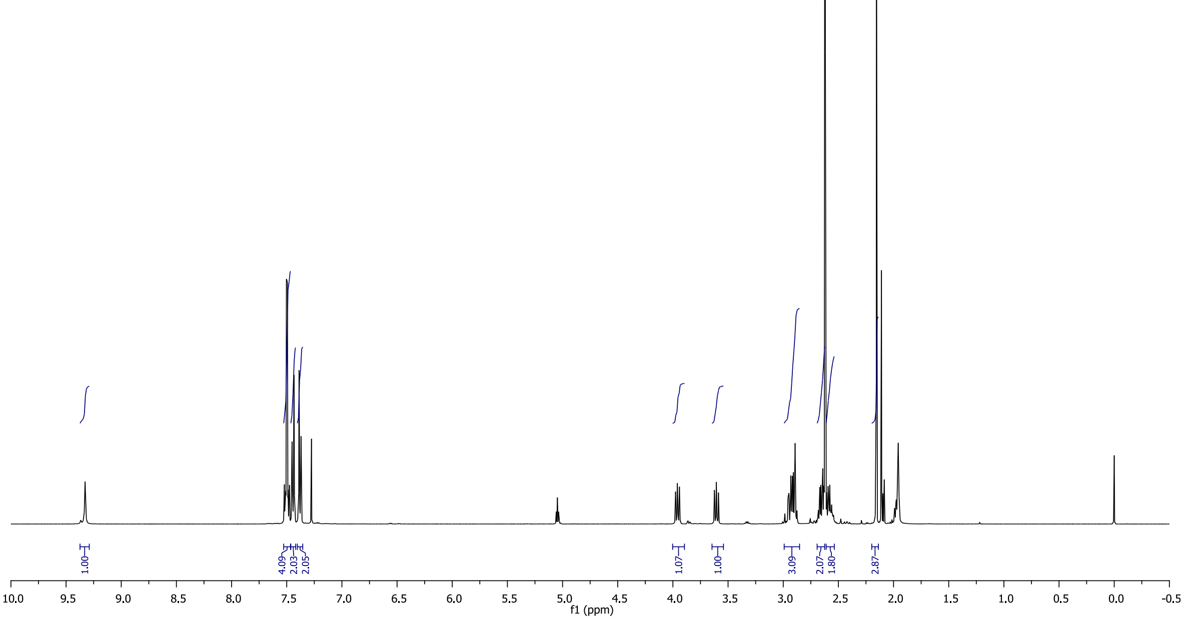

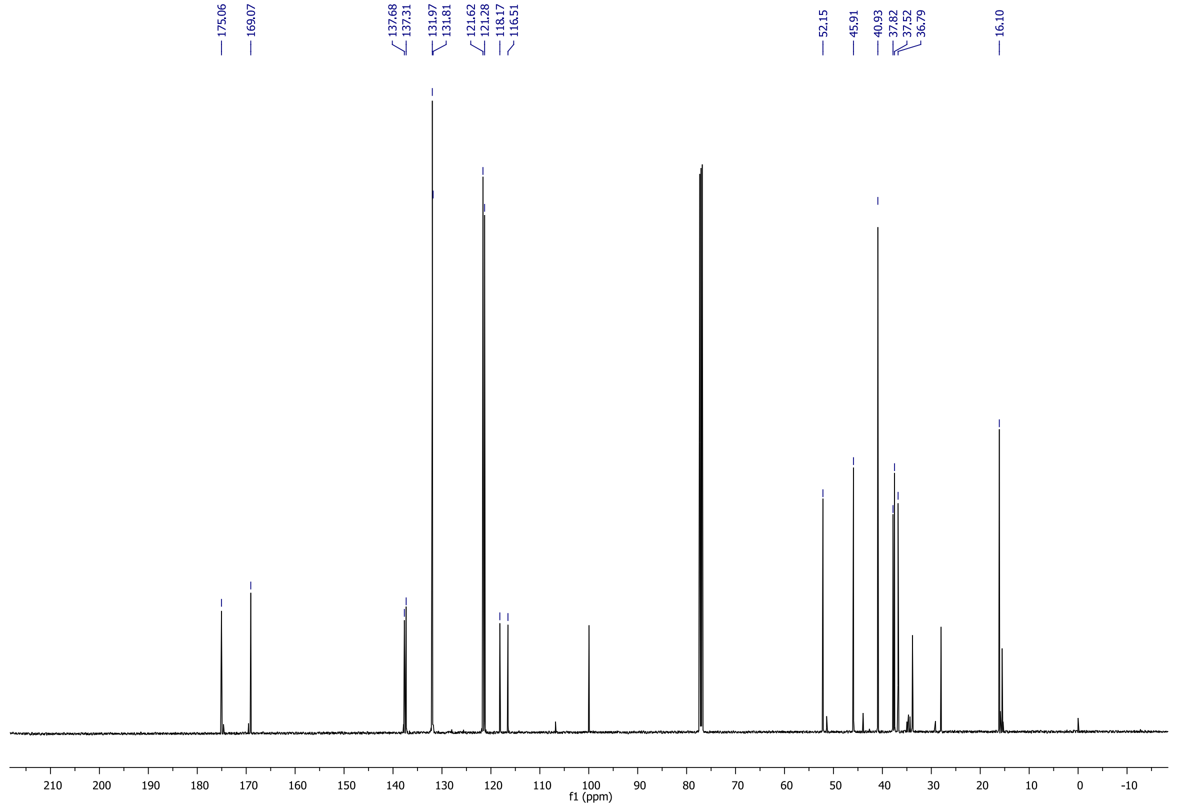

N-(4-Bromophenyl)-2-((3*S*,4*S*)-1-butyl-4-((methylthio)methyl)-2-oxopyrrolidin-3-yl)acetamide (6{*4*,*5*,*7*})

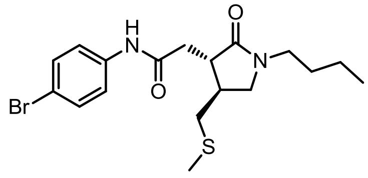

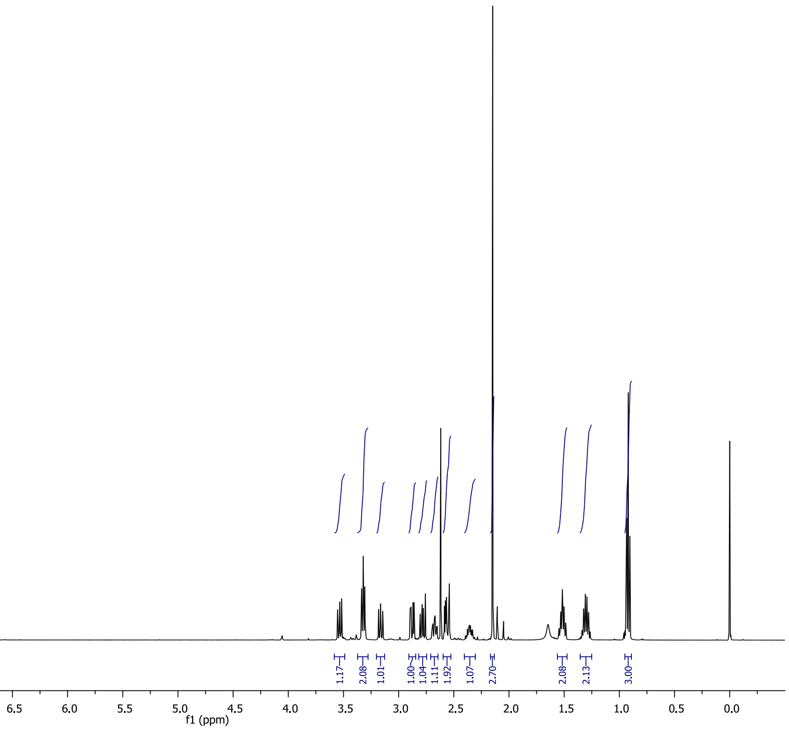

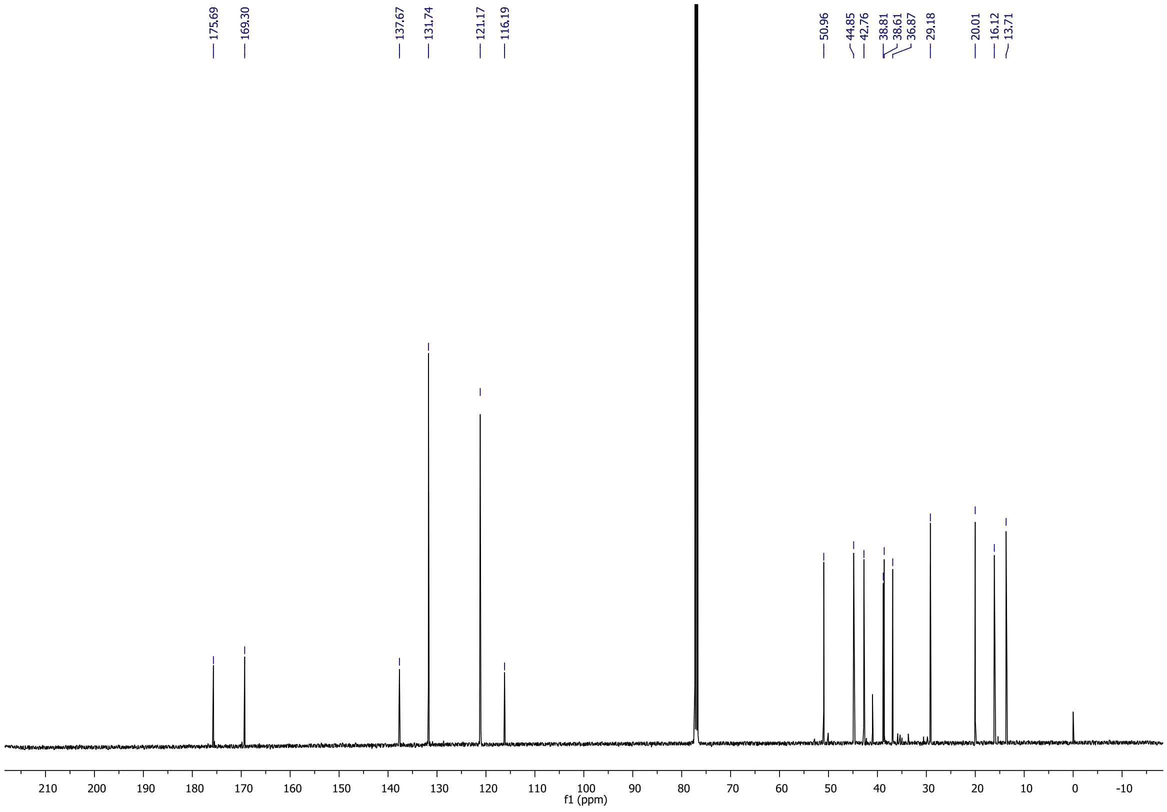

2-((3*S*,4*R*)-1-(4-Bromophenyl)-4-(4-methoxyphenyl)-2-oxopyrrolidin-3-yl)-*N*-(4-methoxyphenyl)acetamide (6{*4,6,4*})

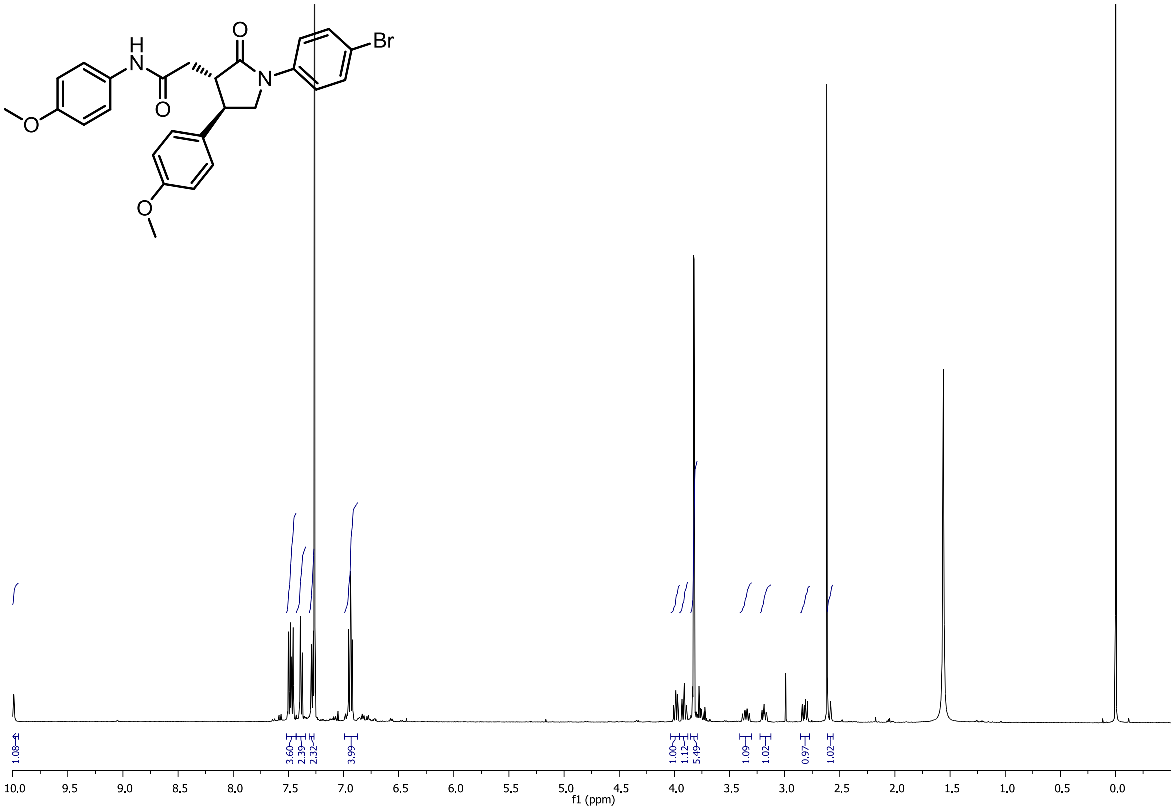

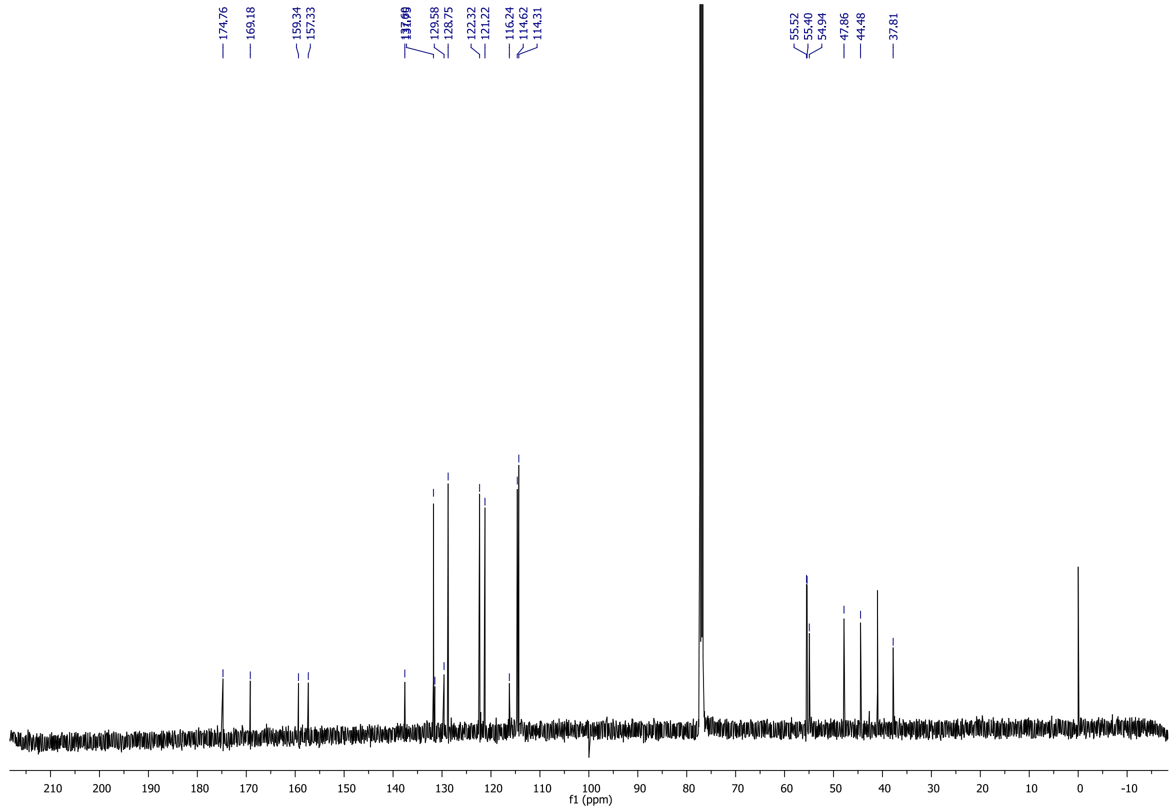

(S)-N-(4-bromophenyl)-2-(4,4-dimethyl-2-oxo-1-phenylpyrrolidin-3-yl)acetamide (6{*4,8,1*})

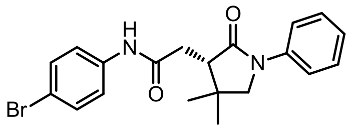

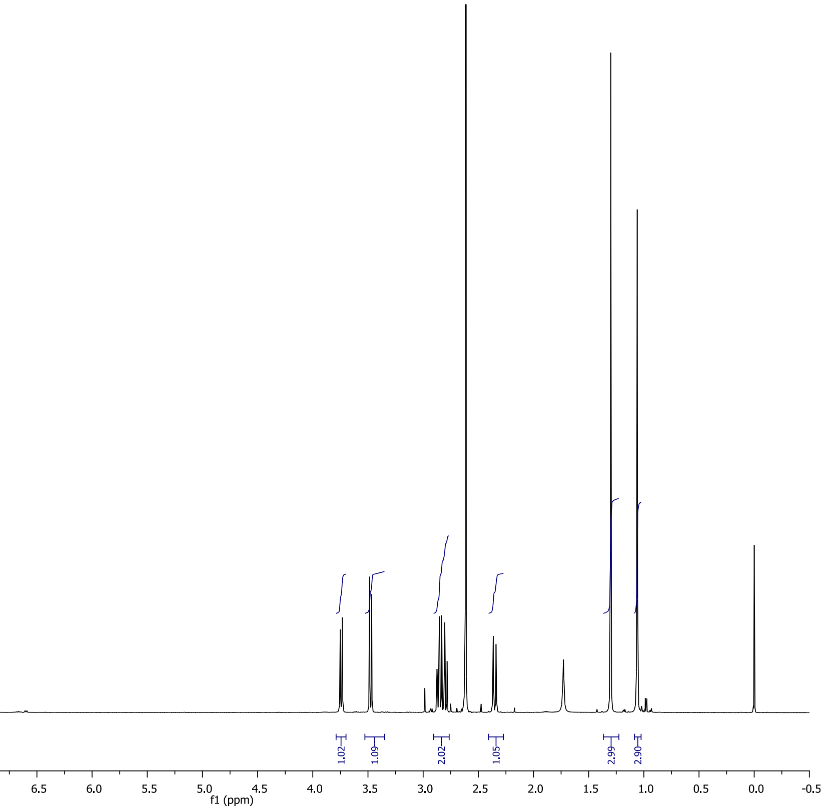

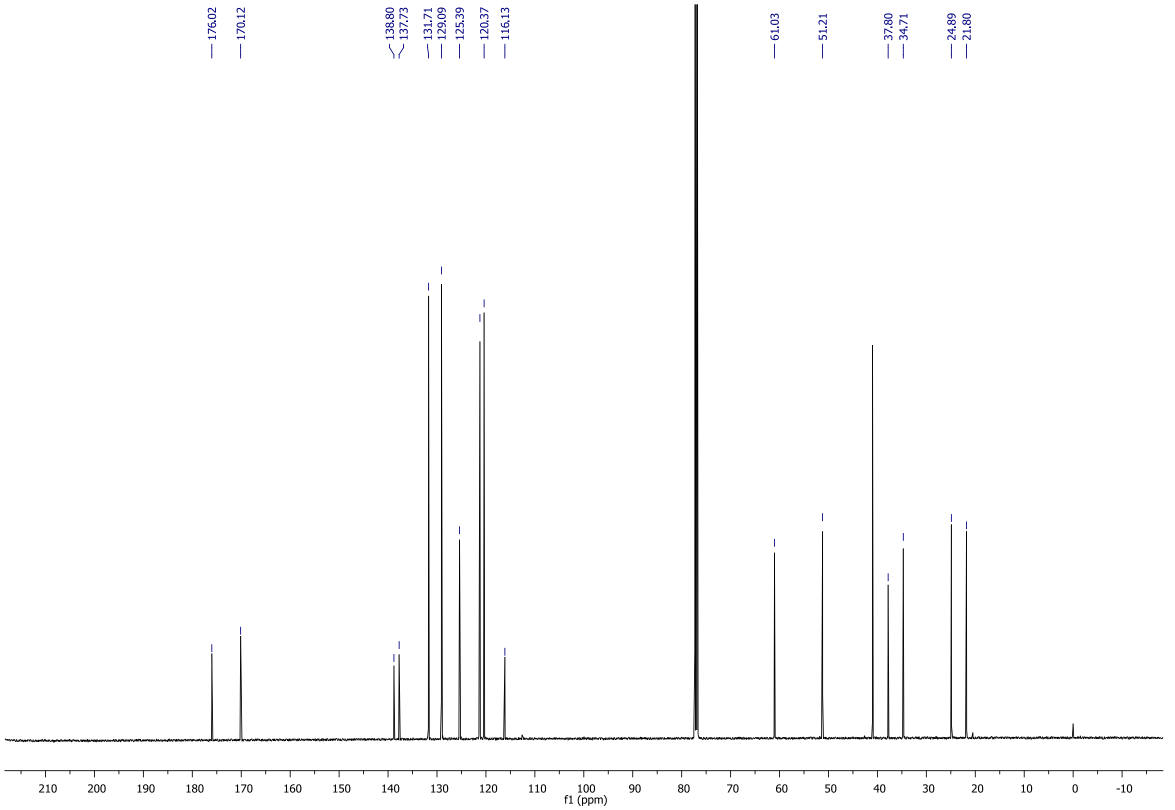

(S)-N-(4-bromophenyl)-2-(1-(4-methoxyphenyl)-4,4-dimethyl-2-oxopyrrolidin-3-yl)acetamide (6{*4,8,4*})

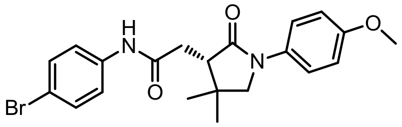

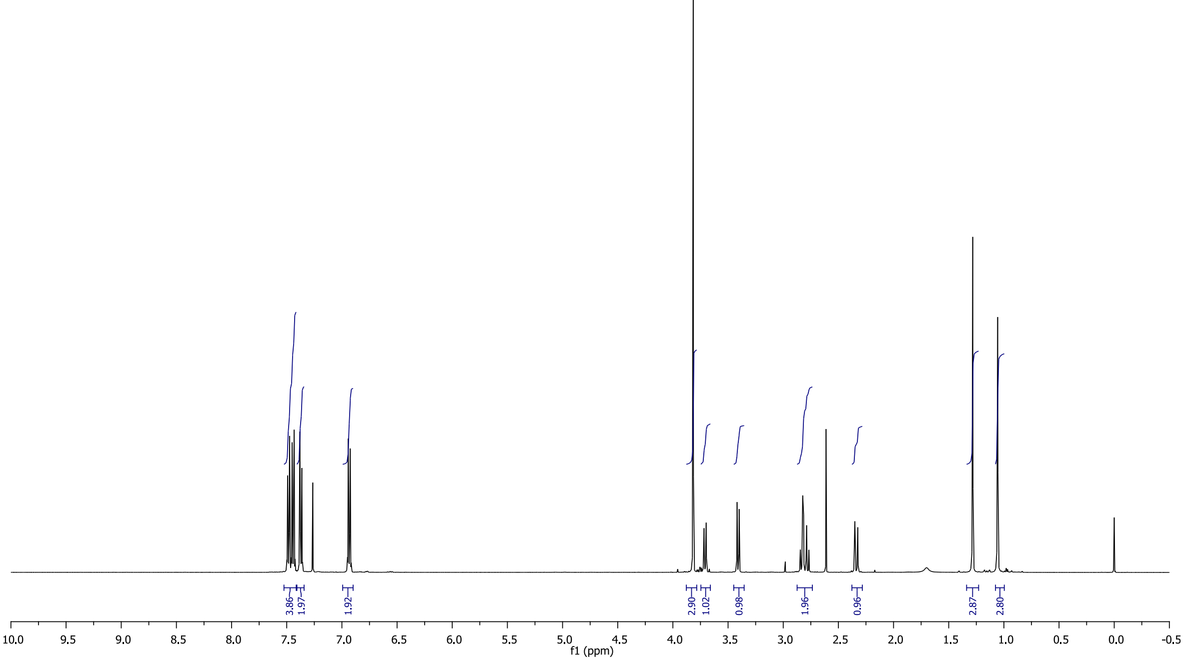

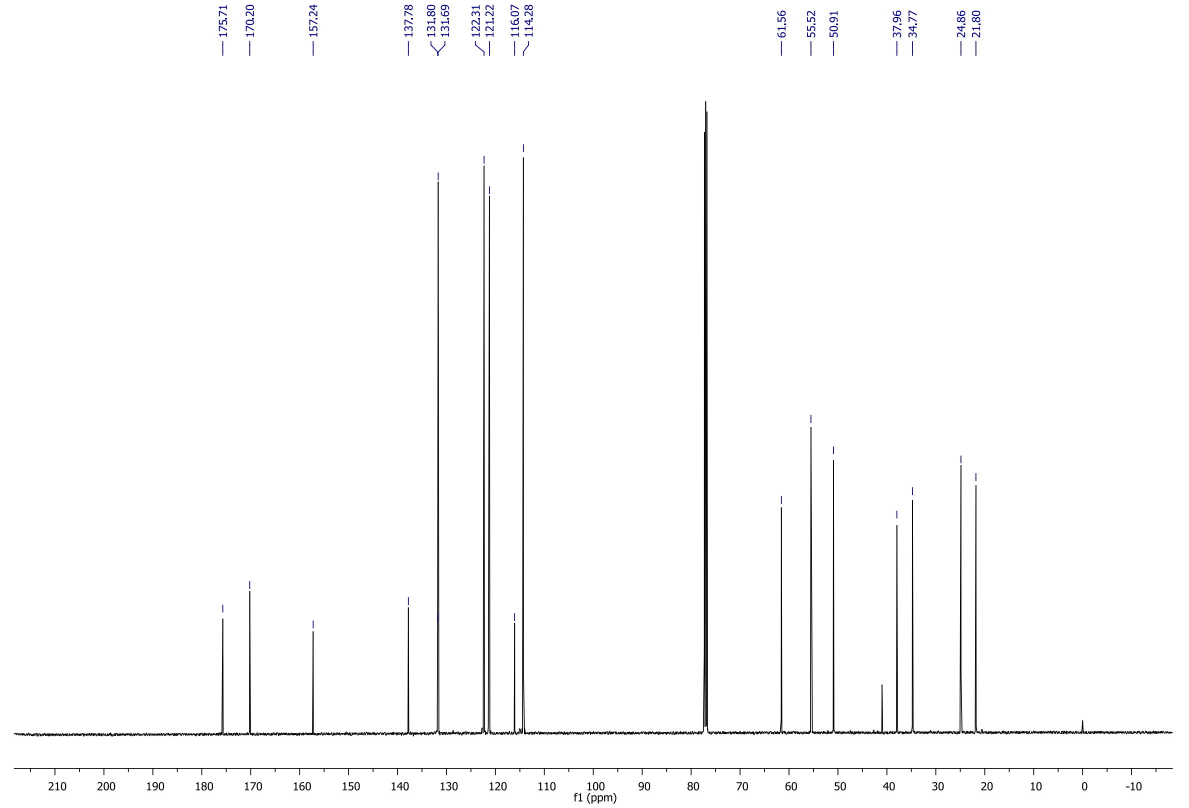

Supplement: File 1 — Results for the four 4 × 2 × 8 libraries using an automated synthesizer, full characterization data for representative compounds, and copies of 1H and 13C NMR spectra for representative compounds. [file Beilstein_J_Org_Chem-08-1804-s001.pdf]
